# Supplementary material for: Fiber deprivation and microbiome-borne curli shift gut bacterial populations and accelerate disease in a mouse model of Parkinson’s disease
Source: Cell Rep. 2023 Sep 8;42(9):113071. doi: 10.1016/j.celrep.2023.113071 (PMC10548091; doi:10.1016/j.celrep.2023.113071)
Supplement: Document S1. Figures S1–S9 and Table S2 [file mmc1.pdf]

Cell Reports, Volume *42*

Supplemental information

Fiber deprivation and microbiome-borne curli shift gut bacterial populations and accelerate disease in a mouse model of Parkinson's disease

Kristopher J. Schmit, Pierre Garcia, Alessia Sciortino, Velma T.E. Aho, Beatriz Pardo Rodriguez, Mélanie H. Thomas, Jean-Jacques Gérardy, Irati Bastero Acha, Rashi Halder, Camille Cialini, Tony Heurtaux, Irina Ostahi, Susheel B. Busi, Léa Grandmougin, Tuesday Lowndes, Yogesh Singh, Eric C. Martens, Michel Mittelbronn, Manuel Buttini, and Paul Wilmes

SUPPLEMENTAL MATERIAL

Supplementary Figures and Legends

Figure S1 | Alpha-synuclein is expressed in the nigro-striatal pathway of Thy1-Syn14 and wild-type mice

(A) Representative microphotographs for total αSyn (pan-αSyn) in the dorsal striatum (top; scale bar: 50µm) and the substantia nigra pars compacta (SNpc; bottom; scale bar: 200µm) between WT (left) and TG (right) mice in TH-positive regions.

(B) Representative western blot gels targeting total αSyn from the dorsal striatum (top) and midbrain (bottom) in 9 months-old TG and WT animals. The band for αSyn was detected at approximatively 14kDa. For subsequent normalization we used here as reference protein α-tubulin, which was detected at approximatively 50kDa. The gels were loaded randomly with TG and WT total protein extracts.

WT, wild-type littermates; TG, Thy1-Syn14

Figure S2 | Alpha and beta diversity assessment for the different challenges

(A) Boxplots for alpha diversity of the different variables/challenges. Genotype and Diet challenges were both altering alpha diversity. No significant changes were induced by gavage alone. P-values were determined using the Kruskal-Wallis test and were corrected for FDR. Data points: Left | WT = 187, TG = 197; Middle | FR = 172, FD = 212; Right | PBS = 44, ΔEC = 168, EC = 172.

(B) Non-metric multi-dimensional scaling (NMDS) plots for beta diversity of the different variables/challenges. Here diet and gavage led to changes in beta diversity However, one has to consider that the PBS gavaged mice were exclusively FD challenged. The dissimilarities were genotype independent. P-values were determined using the adonis test. Data points: Left | WT = 187, TG = 197; Middle | FR = 172, FD = 212; Right | PBS = 44, ΔEC = 168, EC = 172.

WT, wild-type littermates; TG, Thy1-Syn14; FD, fiber deprived; FR, fiber rich; PBS, phosphate buffered saline solution; ΔEC, curli-KO *E. coli*; EC, wild-type curli expressing *E. coli*

Figure S3 | Fiber-deprivation induced increased Firmicutes to Bacteroidetes ratios

Boxplots illustrating the Firmicutes to Bacteroidetes ratios for each tested time point between FR and FD challenged mice. Both genotypes show significant increases in ratios at 9 weeks. Thy1-Syn14 mice do already show significant differences at week 6. P-values were determined using the Kruskal-Wallis test and were corrected for FDR.

Sample sizes:

Week	WT					TG				
	FD PBS	*FR ΔEC*	*FR EC*	*FD ΔEC*	*FD EC*	*FD PBS*	*FR ΔEC*	*FR EC*	*FD ΔEC*	*FD EC*
**0**	4	8	7	7	8	4	8	9	8	8
**1**	3	6	7	6	6	3	8	8	8	8
**2**	4	8	6	8	7	3	7	8	7	8
**5**	4	8	7	5	7	4	6	7	8	8
**6**	4	8	7	6	7	4	6	7	8	8
**9**	4	8	7	5	5	3	5	6	6	6

WT, wild-type littermates; TG, Thy1-Syn14; FD, fiber deprived; FR, fiber rich; PBS, phosphate buffered saline solution; ΔEC, curli-KO E. coli; EC, wild-type curli expressing E. coli

Figure S4 | Differential taxa abundance over time is diet regulated

(A) Complex heatmap visualizing the effect of different challenges on the relative abundance changes over time. The taxa were subdivided into three different groups according to their relative abundances: High, at least for one time point, there was an average relative abundance of 10% or higher for at least one treatment group; Mid, at least for one time point, there was an average relative abundance between at least 1 and maximum 10% for at least one treatment group; Low, less than 1%. All data was scaled and centered for each row. Main effect is seen between FR and FD challenges. Analogous to what we had seen before, the FD diet challenge was the main driver of microbial abundance changes FD challenged mice had especially higher levels in *Intestinimonas, Odoribacter, Colidextribacter, Rikenellaceae RC9 gut group* and *Akkermansia,* and decreased levels in *Lachnospiraceae NK4A136 group* and *Lactobacillus* over time (see also Figure 2C). *Alistipes, Faecalibaculum, Bacteroides, Lachnoclostridium* and *Lachnospiraceae UCG_006* on the other hand saw fluctuations over time. With the exception of *Faecalibaculum*, they first increased before dropping back down to initial levels in FD challenged mice. Inversely, levels of *Faecalibaculum* first dropped significantly in FD diet challenged mice, before even

surpassing its measured levels in FR diet groups. For some taxa, we observed also difference between genotypes.

(B-D) Boxplots illustrating the quantitative real time PCR results specifically targeting *E. coli.* For clarification: values are inversely related to the amount of *E. coli* in fecal samples.

(B) The FD diet (Kruskal-Wallis, p = 4.55E-12) challenge as well as (C) PBS gavage (Mann Whitney U, FDR = 0.027) leads overall to significantly higher levels of *E. coli* in feces. Data points: Diet | FR = 172, FD = 212; Gavages | PBS = 44, ΔEC = 168, EC = 172.

(D) Boxplots illustrating the different challenge groups for WT (top row) and TG (bottom row) mice separately at different time points (week 0, week 1, week 2, week 5, week 6, week 9). In WT mice we saw significant group differences at Week 1 (Kruskal-Wallis, p = 0.0157) and Week 5 (Kruskal-Wallis, p = 0.0366), whereas in TG animals we saw significant differences between the groups at Week 2 (Kruskal-Wallis, p = 0.00793) and Week 5 (Kruskal-Wallis, p = 0.0267).

Sample sizes:

**Week**	**WT**					**TG**				
	FD PBS	*FR ΔEC*	*FR EC*	*FD ΔEC*	*FD EC*	*FD PBS*	*FR ΔEC*	*FR EC*	*FD ΔEC*	*FD EC*
**0**	4	8	7	7	8	4	8	9	8	8
**1**	3	6	7	6	6	3	8	8	8	8
**2**	4	8	6	8	7	3	7	8	7	8
**5**	4	8	7	5	7	4	6	7	8	8
**6**	4	8	7	6	7	4	6	7	8	8
**9**	4	8	7	5	5	3	5	6	6	6

WT, wild-type littermates; TG, Thy1-Syn14; FD, fiber deprived; FR, fiber rich; PBS, phosphate buffered saline solution; ΔEC, curli-KO *E. coli*; EC, wild-type curli expressing *E. coli*

Figure S5 | Invariant inner mucus thickness and outer mucus to microbial diversity associations

(A) Barplot illustrating the combined mucus thickness from the pilot study in WT and TG mice after 1 week or 3 weeks of FD diet challenge.

(B) Inner mucus thickness does not vary significantly between the different groups. To note is however, the ticker inner mucus layer after 1 week in TG mice on the FD diet compared to its WT equivalent. This could indicate an early and greater compensatory/rescue mechanism in TG

animals. This is not observed anymore after 3 weeks. Sample sizes: 1-Week | WT – FR: n = 3, FD: n = 3; TG – FR: n = 2, FD: n = 3; 3-Week | WT – FR: n = 1, FD: n = 3; TG – FR: n = 2, FD: n = 2.

(C) Outer mucus thickness is, independent of genotype, significantly thinner due to the FD diet challenge after 1 week (Kruskal-Wallis, p = 0.004) and 3 weeks (Kruskal-Wallis, p = 0.036), respectively. Sample sizes: 1-Week | WT – FR: n = 3, FD: n = 3; TG – FR: n = 2, FD: n = 3; 3-Week | WT – FR: n = 1, FD: n = 3; TG – FR: n = 2, FD: n = 2.

(D) Barplot illustrating the combined mucus thickness in all different treatment groups.

(E) Inner mucus thickness does not vary significantly between the different treatment groups in the separate genotypes. To note is however, the significant ticker inner mucus layer in the TG FD EC group compared to its WT equivalent. This inferred a greater compensatory/rescue mechanism in TG animals. Sample sizes: WT FD PBS, n = 3; WT FR ΔEC, n = 5; WT FR EC, n = 4; WT FD ΔEC, n = 3; WT FD EC, n = 7; TG FD PBS, n = 2; TG FR ΔEC, n = 4; TG FR EC, n = 6; TG FD ΔEC, n = 6; TG FD EC, n = 7.

(F - G) Scatterplots of Spearman rank tests comparing alpha diversity (y-axis) and mucus thickness (x-axis) (C) overall, (D) for the different diet groups, and (D) combining genotype and diet groups

WT, wild-type littermates; TG, Thy1-Syn14; FD, fiber deprived; FR, fiber rich; PBS, phosphate buffered saline solution; ΔEC, curli-KO *E. coli*; EC, wild-type curli expressing *E. coli*

Figure S6 | Gross motor and sensorimotor functions are transgene driven

(A - B) Longitudinal gross motor function monitoring visualized in line plots indicating the median value changes for the different treatment groups. Alpha-synuclein overexpression was the driver of motor deficits in transgenic animals. (A) Hindlimb clasping scores increased within all transgenic groups (dotted lines), independent of their treatment. Differences between WT and TG animals were significant from baseline to week 9. All p-values were determined using the Mann-Whitney U test and FDR to correct for multiple comparison. (B) Grip strength results confirm that there is only a difference between the genotype and there was no observable difference due to any kind of treatment. Differences between WT and TG animals were significant from baseline to

week 9. All p-values were determined using the Mann-Whitney U test and FDR to correct for multiple comparisons.

(C) Boxplots illustrating the latency for touch in the different treatment groups (x-axis) for WT (red) and TG (blue) animals after 9 weeks. The time of touch refers to the sensory ability of the mice. Here we saw a clear significance between genotypes (Kruskal-Wallis, corrected for FDR; $p < 0.0001$). More specifically there are significant differences in the FR ΔEC, FR EC, and FD EC between genotypes. Sample size: WT FD PBS, n = 4; WT FR ΔEC, n = 8; WT FR EC, n = 6; WT FD ΔEC, n = 5; WT FD EC, n = 6; TG FD PBS, n = 4; TG FR ΔEC, n = 5; TG FR EC, n = 7; TG FD ΔEC, n = 8; TG FD EC, n = 7.

All p-values were determined using the Mann-Whitney U test.

WT, wild-type littermates; TG, Thy1-Syn14; FD, fiber deprived; FR, fiber rich; PBS, phosphate buffered saline solution; ΔEC, curli-KO *E. coli*; EC, wild-type curli expressing *E. coli*

Figure S7 | Not all immunopositive pathological protein inclusions are Thio-S positive in different models of neurodegeneration.

Left column shows immunostaining of different protein inclusions. Right column shows Thio-S staining of the same location. The two top rows show pS129-αSyn immunostaining and Thio-S staining of the dorsal striatum (top row) and SNpc (ssyond row) of a TG mouse after FD and EC challenges, used in this study. The third row shows a mouse 3 month after intrastriatal injection of αSyn preformed fibrils, as described in one of our previous studies [86]. None of the αSyn inclusions are Thio-S positive, but neurodegeneration was detected in both. The fourth row shows AT8 immunostaining for hyperphosphorylated tau and Thio-S staining of tau inclusions in the hippocampal CA1 of a 17-month-old hTau transgenic mice [134]. The fifth row shows 82E1 immunostaining and Thio-S staining of amyloid plaques in the hippocampal dentate gyrus of a 20-month-old mutated hAPP transgenic mouse [135]. Note that numerous immunopositive structures, in both models, are not positive for Thio-S. Scale bar: 60 μm.

Figure S8 | Minimal or no central and peripheral inflammation in Thy1-Syn14 mice after diet and *E. coli* challenges

(A) Neuroinflammation was assessed using the microglia marker Iba1. Quantification was performed by measuring the area occupied by Iba1-positive microglia in TH-positive regions of the SNpc and the dorsal striatum. No significant changes were observed. Sample size: WT FD PBS, n = 4; WT FR ΔEC, n = 8; WT FR EC, n = 6; WT FD ΔEC, n = 5; WT FD EC, n = 7; TG FD PBS, n = 4; TG FR ΔEC, n = 5; TG FR EC, n = 7; TG FD ΔEC, n = 8; TG FD EC, n = 8.

(B) Boxplots illustrating changes in calprotectin between FR and FD diet challenged animals at different time points. Fecal calprotectin levels were subtle but significantly higher in WT mice at weeks 2 (Kruskal-Wallis, p = 0.025), 6 (Kruskal-Wallis, p = 0.017) and in fecal matter from the proximal colon collected at post-mortem (PM) (Kruskal-Wallis, p = 0.002) and in TG mice at weeks 1 (Kruskal-Wallis, p = 0.02), 2 (Kruskal-Wallis, p = 0.003), 6 (Kruskal-Wallis, p = 0.042) and in fecal matter from the proximal colon collected at PM (Kruskal-Wallis, p = 0.059) fed the FD diet.

Sample sizes:

Week	WT					TG				
	FD PBS	*FR ΔEC*	*FR EC*	*FD ΔEC*	*FD EC*	*FD PBS*	*FR ΔEC*	*FR EC*	*FD ΔEC*	*FD EC*
**1**	2	7	5	3	3	3	4	6	7	6
**2**	2	7	5	4	4	3	4	6	7	7
**6**	1	4	3	0	2	2	3	2	3	4
**9**	4	8	6	5	3	4	5	7	8	8
**PM**	3	7	6	5	5	3	2	6	7	4

(C) At the end of the treatments, plasma levels of many cytokines/chemokines (Ccl1 (Kruskal-Wallis, p = 0.0305), Ccl11 (Kruskal-Wallis, p = 0.0305), Ccl2 (Kruskal-Wallis, p = 0.0388), Ccl3 (Kruskal-Wallis, p = 0.032), Ccl4 (Kruskal-Wallis, p = 0.0184), Ccl5 (Kruskal-Wallis, p = 0.0107), Cxcl1 (Kruskal-Wallis, p = 0.025), Cxcl9 (Kruskal-Wallis, p = 0.0388), G-Csf (Kruskal-Wallis, p = 0.00604), Il2 p70 (Kruskal-Wallis, p = 0.0184), Il16 (Kruskal-Wallis, p = 0.032), Il17 (Kruskal-Wallis, p = 0.0101), Il1a (Kruskal-Wallis, p = 0.0407), Il1b (Kruskal-Wallis, p = 0.0166), Il1ra (Kruskal-Wallis, p = 0.032), Il23 (Kruskal-Wallis, p = 0.032), Il5 (Kruskal-Wallis, p = 0.00173), Il7 (Kruskal-Wallis, p = 0.0446), Trem-1 (Kruskal-Wallis, p = 0.0276)) had overall significantly lower levels in TG than in WT mice. When we looked further into how diet, the gavages or their combination might have impacted the chemo-/cytokine levels, we saw that in mice fed the FD diet protein levels for Ccl3, Ccl5, Cxcl2, Il1ra, Il27 and Il6 were lower as compared to FR diet fed mice. When we further looked into how the challenges affected plasma in the individual genotypes, we

saw that in TG mice fed the FD diet levels for Ccl5 ($p = 0.009$), Cxcl2 ($p = 0.013$) and Il1ra ($p = 0.044$) were significantly lower (not shown in detail). The lowest levels for these proteins, especially for Ccl5 ($p = 0.009$) and Cxcl2 ($p = 0.015$), were observed when TG mice were doubly FD EC challenged. This unexpected finding may be due to the overexpression of human αSyn in immune cells of TG mice, which may lead to their immune suppression. See main text for more details. Sample size: WT FD PBS, n = 3; WT FR ΔEC, n = 5; WT FR EC, n = 6; WT FD ΔEC, n = 5; WT FD EC, n = 6; TG FD PBS, n = 3; TG FR ΔEC, n = 5; TG FR EC, n = 6; TG FD ΔEC, n = 5; TG FD EC, n = 6.

WT, wild-type littermates; TG, Thy1-Syn14; FD, fiber deprived; FR, fiber rich; PBS, phosphate buffered saline solution; ΔEC, curli-KO *E. coli*; EC, wild-type curli expressing *E. coli*; PM, post-mortem collected fecal matter from the proximal colon

Figure S9 | Experimental design and in-life experiments

(A) Hierarchy graphs illustrating the experimental design. See details in Material and Methods. The white on black background numbers refer to the numbers of animals we were left with at the end of the in-life phase.

(B) Graph showing which treatments were given or tests were performed during the in-life phase. Gross motor functions were tested weekly, while the adhesive removal test was only performed twice, at start and end of the in-life phase. Feces were checked weekly and afterwards six time points were chosen for analysis. Mice were gavaged weekly starting at week 2 for a total 8 times and their food was changed every second week

WT, wild-type littermates; TG, Thy1-Syn14; FD, fiber deprived; FR, fiber rich; PBS, phosphate buffered saline solution; ΔEC, curli-KO *E. coli*; EC, wild-type curli expressing *E. coli*

Supplementary Tables and Legends

Table S1 | Comparison of the 16S rRNA sequencing results to known taxa altered in stool samples of patients with PD (adapted from Boertien et al., 2019)

Table S2 | Abbreviations for the main figures

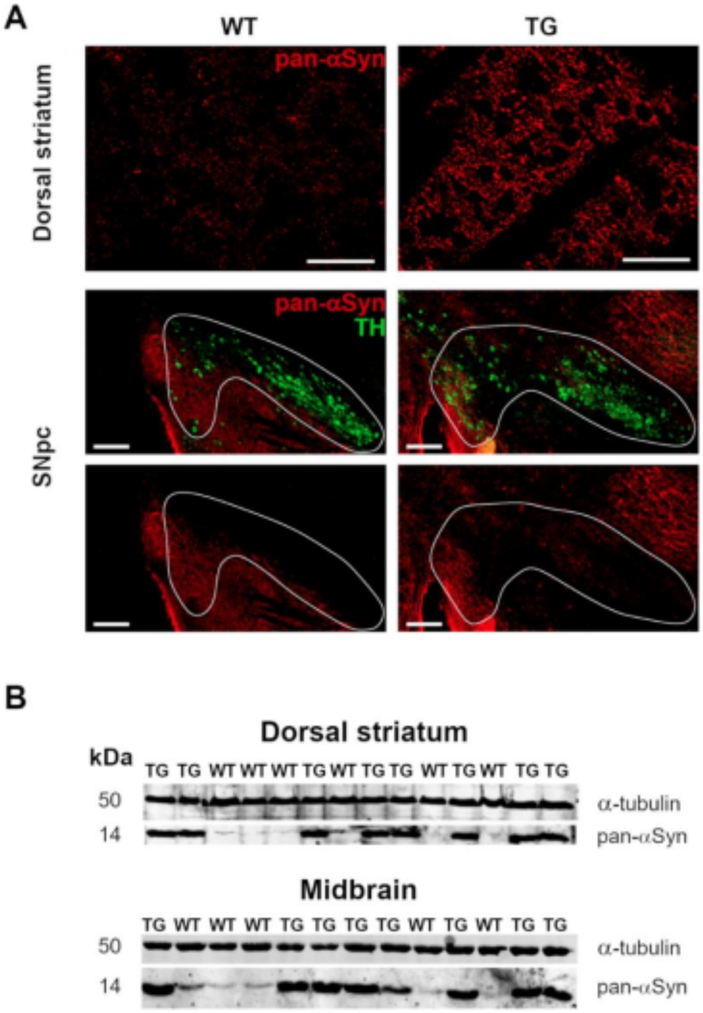

Figure S1 | Alpha-synuclein is expressed in the nigro-striatal pathway of Thy1-Syn14 and wild-type mice

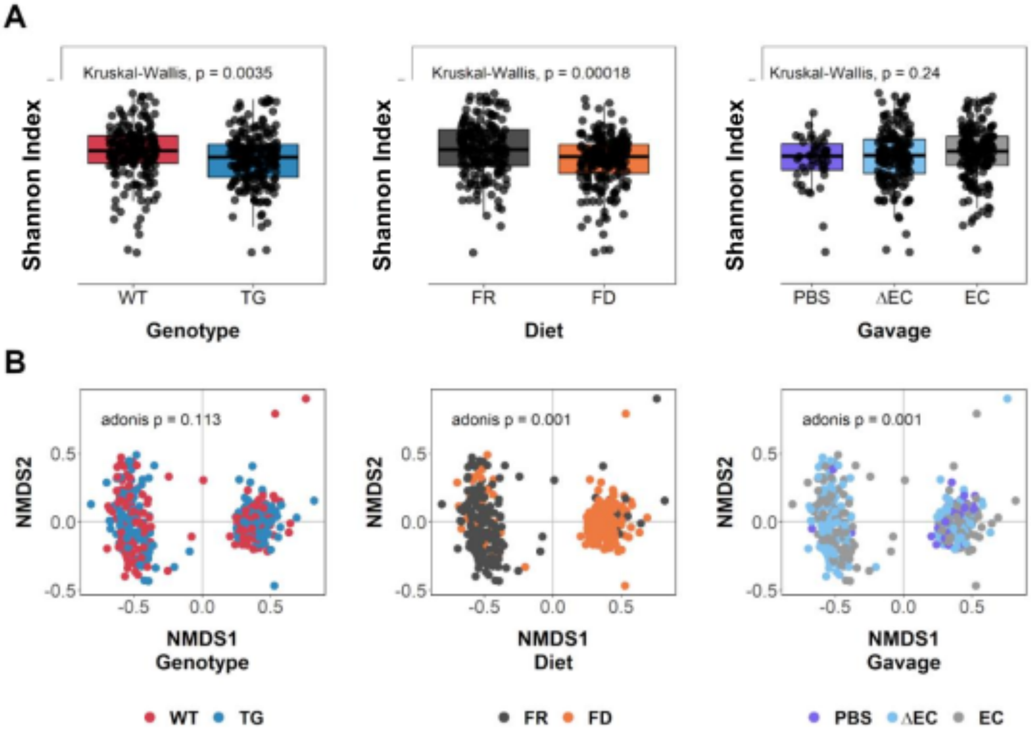

Figure S2 | Alpha and beta diversity assessment for the different challenges

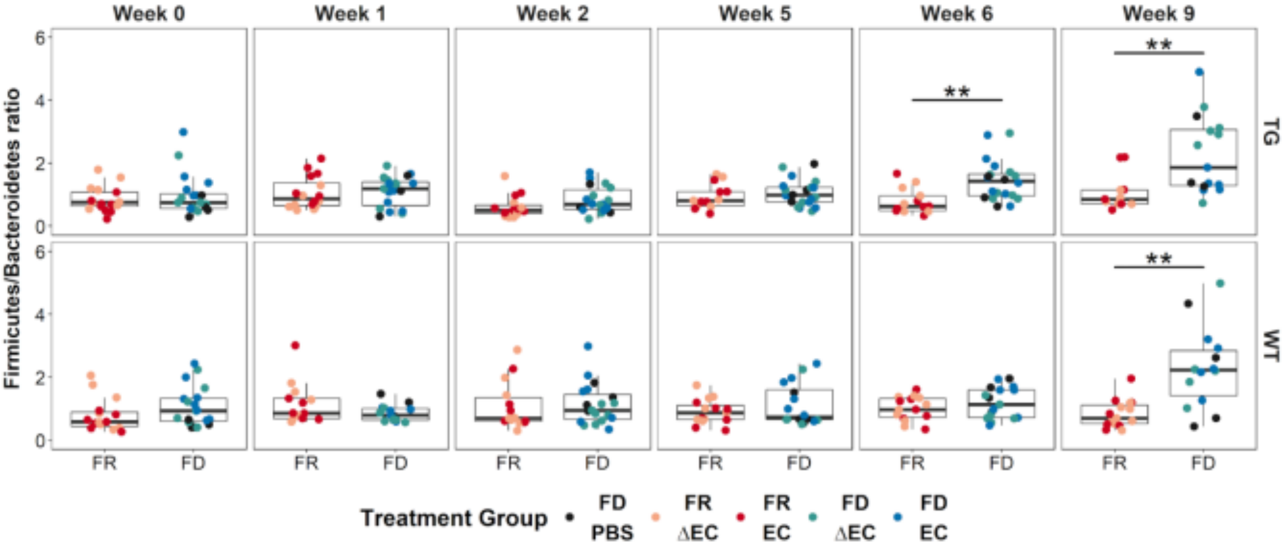

Figure S3 | Fiber-deprivation induced increased Firmicutes to Bacteroidetes ratios

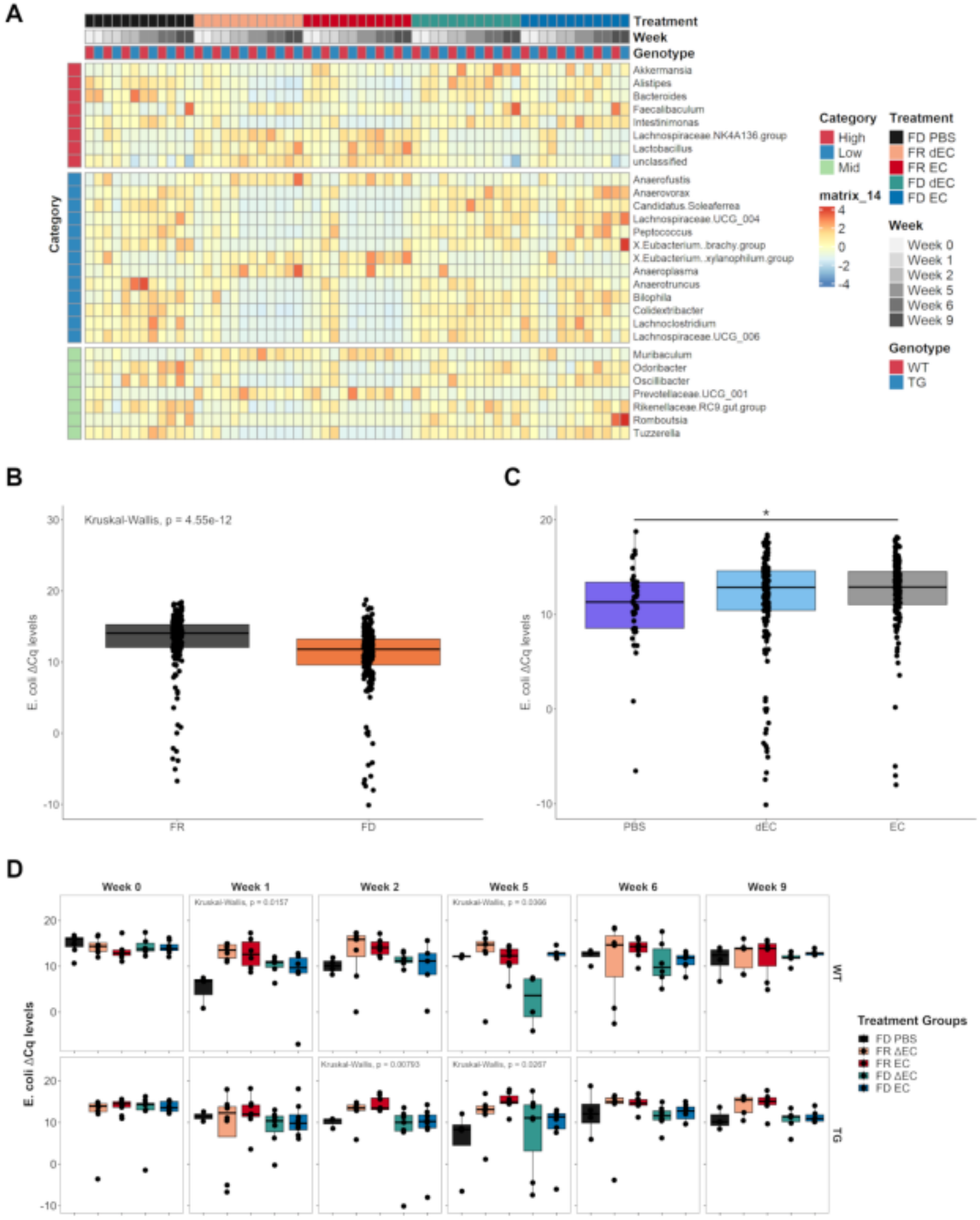

Figure S4 | Differential taxa abundance over time is diet regulated

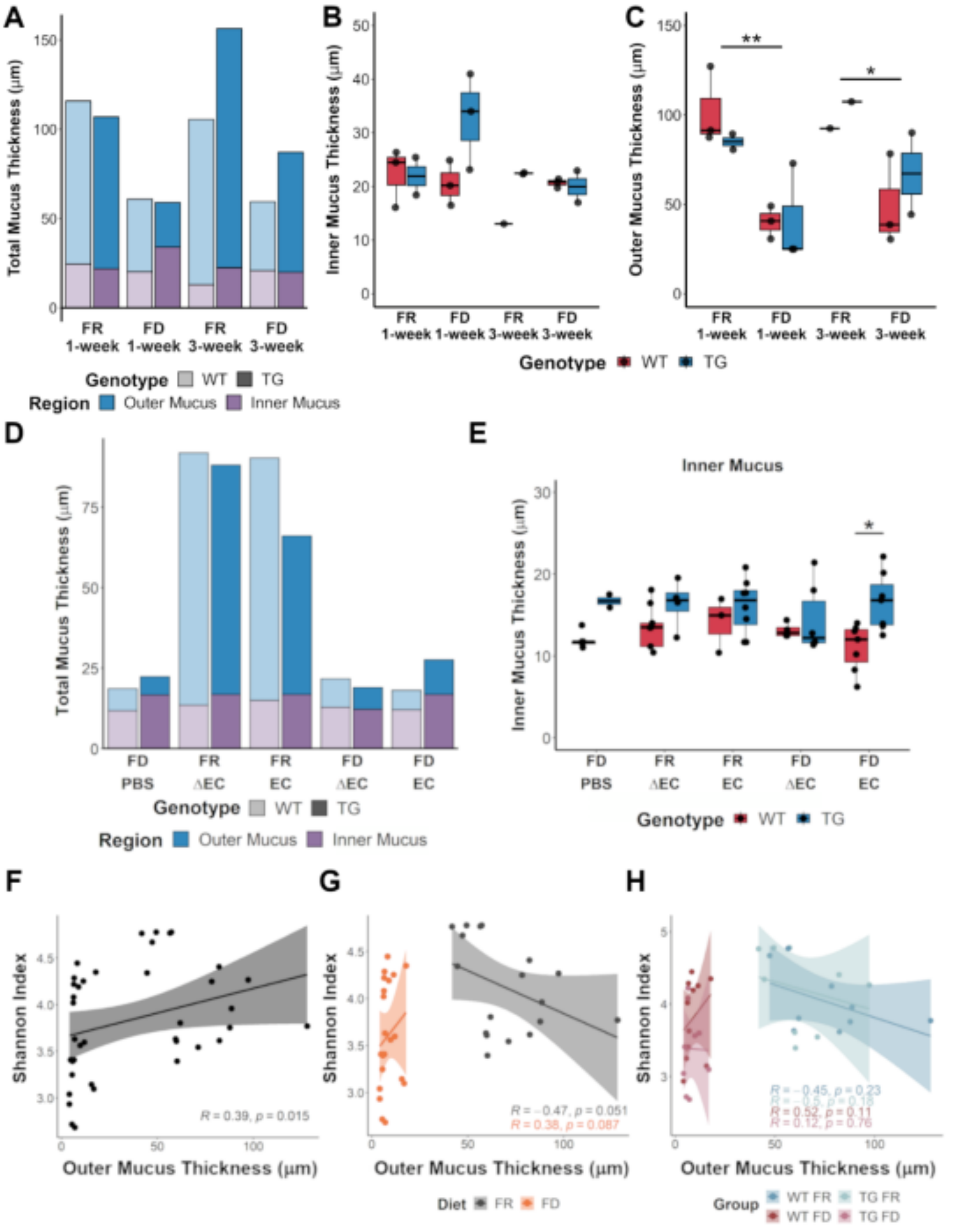

Figure S5 | Invariant inner mucus thickness and outer mucus to microbial diversity associations

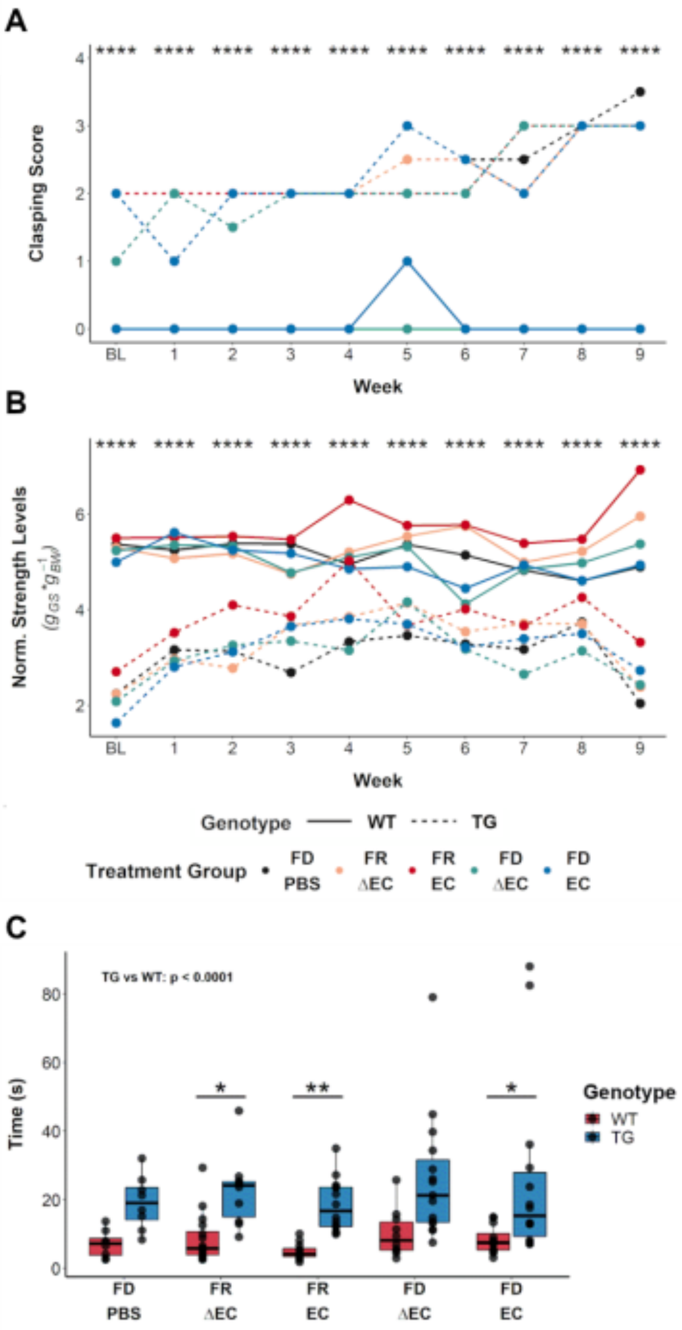

Figure S6 | Gross motor and sensorimotor functions are transgene driven

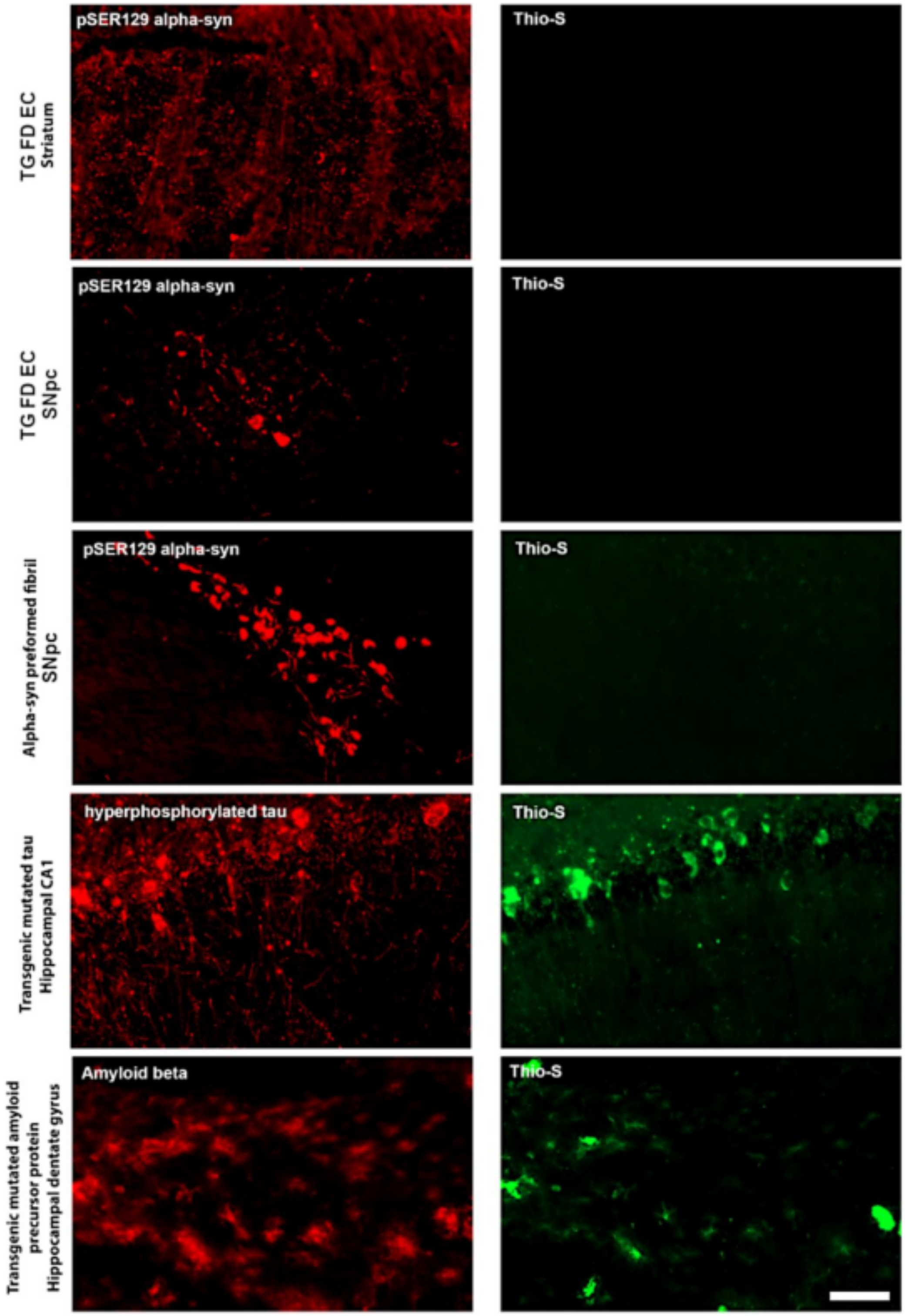

Figure S7 | Not all immunopositive pathological protein inclusions are Thio-S positive in different models of neurodegeneration.

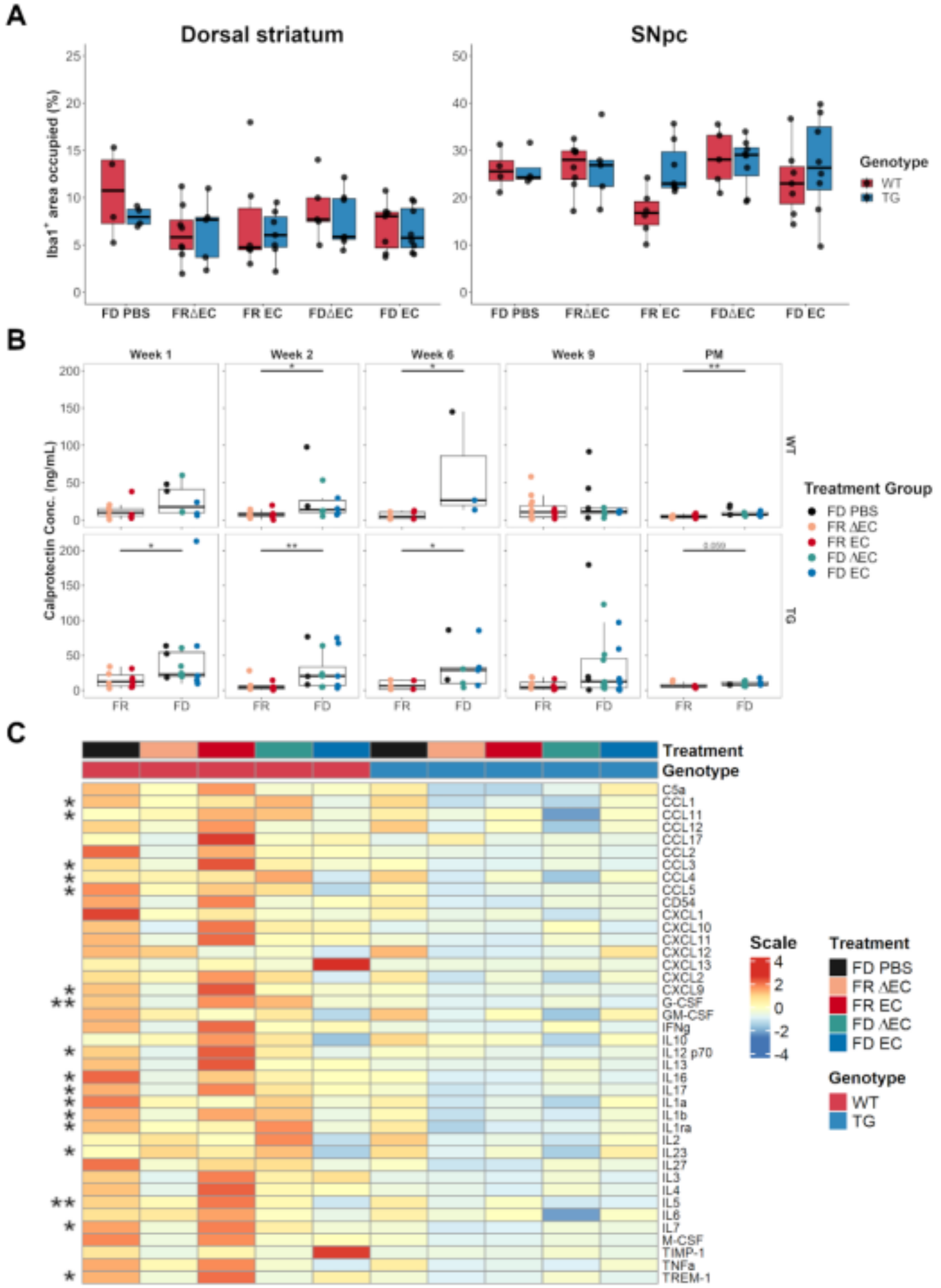

Figure S8 | Minimal or no central and peripheral inflammation in Thy1-Syn14 mice after diet and *E.coli* challenges

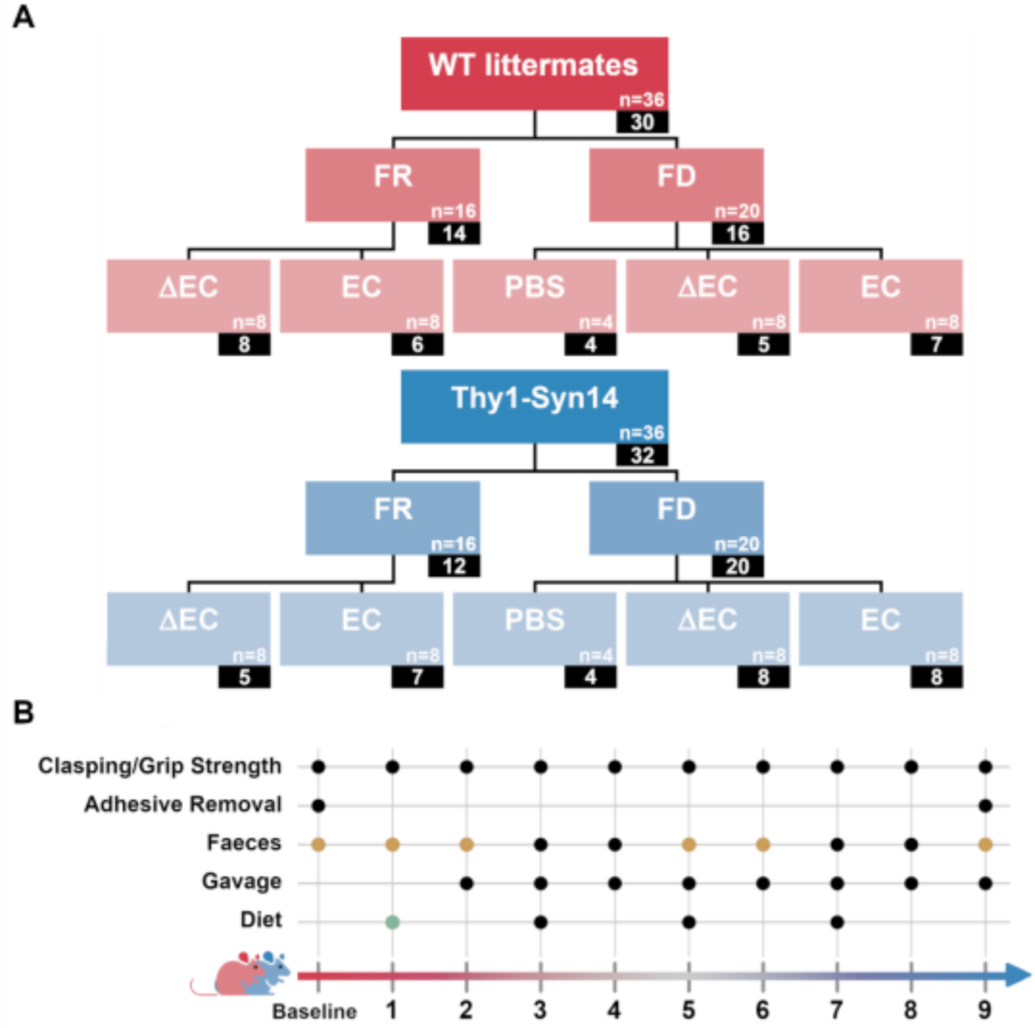

Figure S9 | Experimental design and in-life experiments

Supplementary Tables

Table S1 | Comparison of the 16S rRNA sequencing results to known taxa altered in stool samples of patients with PD (adapted from Boertien et al., 2019)

FR DEC						FR EC						FD DEC						FD EC						Treatment group
2		5		9		2		5		9		2		5		9		2		5		9		Week
WT	TG	WT	TG	WT	TG	WT	TG	WT	TG	WT	TG	WT	TG	WT	TG	WT	TG	WT	TG	WT	TG	WT	TG	Genotype
↓	↓	-	↓	-	↓	-	↓	↓	↓	↓	↓	↑	↑	↑	↑	↑	↑	↓	↑	↓	↑	↓	↑	
-	↓	-	↓	-	↓	-	↓	-	↓	-	↓	↗	↑	↗	↗	↑	↑	↘	↑	↓	↑	↓	↗	
↓	↓	-	↓	-	↓	↗	↓	↓	↓	↓	↓	↗	↑	↗	↑	↑	↑	↓	↑	↓	↑	↓	↗	
↘	-	↘	↗	-	↑	↗	↑	↗	↑	↑	↑	↓	-	↓	-	↓	-	-	-	-	-	-	-	
↓	-	↘	↗	-	↑	↗	↑	↗	↑	↑	↑	↓	↘	↓	↓	↓	-	↓	-	↓	↓	↓	-	
N/A						N/A						N/A						N/A						
N/A						N/A						N/A						N/A						
N/A						N/A						N/A						N/A						
N/A						N/A						N/A						N/A						
↗	↓	↗	-	↘	-	↗	-	↗	↗	↗	↗	↘	-	↘	↗	↑	↑	-	↘	-	↘	↑	↗	
↗	↘	↗	-	↘	↘	↗	-	↗	↗	-	↗	↘	↘	↘	↘	-	↘	-	↘	↗	-	↓	-	
↗	↘	↗	↘	↗	↘	-	-	-	-	↑	↑	-	-	-	↑	↑	↑	↓	↘	↘	↘	↘	-	
-	↑	-	-	-	-	-	↗	-	-	-	↘	↓	-	↓		↓	↓	N/A						
N/A						N/A						N/A						N/A						
N/A						N/A						N/A						N/A						
-	↗	-	-	-	-	↘	↗	-	-	↘	↘	-	-	-	-	↓	↓	-	↗	-	-	↓	↓	
↘	↘	↗	↗	↗	↗	↘	-	↘	-	↗	-	↘	↗	↓	↗	↑	↑	↘	↓	↘	↓	↑	↗	
↗	↘	-	↘	↘	↘	↗	↘	↘	↘	↘	-	↑	↘	↑	↘	↗	↓	-	-	↗	↗	↓	↓	

Phylum	Family	Genus	FD PBS					
			2		5		9	
			WT	TG	WT	TG	WT	TG
Verrucomicrobia			↑	↑	↗	-	↑	↗
Verrucomicrobia	Akkermansiaceaea		↗	↑	↗	-	↗	↗
Verrucomicrobia	Akkermansiaceaea	Akkermansia	↑	↑	↗	-	↑	↑
Firmicutes	Lactobacillaceae		-	-	-	-	-	-
Firmicutes	Lactobacillaceae	Lactobacillus	↗	↘	↓	↓	-	-
Actinobacteria	Bifidobacteriaceae		N/A					
Actinobacteria	Bifidobacteriaceae	Bifidobacterium	N/A					
Proteobacteria	Enterobactericiaceae		N/A					
Firmicutes	Enterococcaceae		N/A					
Firmicutes			↑	-	↗	↑	↑	↑
Firmicutes	Lachnospiraceae		↗	-	-	↑	↑	-
Firmicutes	Lachnospiraceae	Roseburia	↗	-	↗	-	↗	-
Bacteriodetes	Prevotellaceae		↘	↘	↘	↘	↘	↘
Bacteriodetes	Prevotellaceae	Prevotella	N/A					
Firmicutes	Clostridiaceae	Faecalibacterium	N/A					
Bacteriodetes			↓	↘	↘	↘	↓	↓
Firmicutes	Erysipelotrichacceae		↗	↗	↘	-	↘	↑
Firmicutes	Ruminococcaceae		↗	↗	↑	↗	↗	-

Table S2 | Abbreviations for the main figures

Abbreviation	Definition
Snca	endogenous gene for aSyn
SNCA	human aSyn transgene
3M	3-month-old
6M	6-month-old
9M	9-month-old
13M	13-month-old
WT	wild-type littermates
TG	Thy1-Syn14 mice
FD	fiber-deprived
FR	fiber-rich
PBS	phosphate buffered saline solution
DEC	curli-KO E. coli
EC	wild-type curli expressing E. coli
CNS	central nervous system
ENS	enteric nervous system
SNpc	Substantia Nigra pars compacta
ZO-1	zonula occludens-1
