# Supplementary material for: Fiber deprivation and microbiome-borne curli shift gut bacterial populations and accelerate disease in a mouse model of Parkinson’s disease
Source: Cell Rep. 2023 Sep 8;42(9):113071. doi: 10.1016/j.celrep.2023.113071 (PMC10548091; doi:10.1016/j.celrep.2023.113071)
Supplement: Document S2. Article plus supplemental information [file mmc3.pdf]

Cell Reports

Article

Fiber deprivation and microbiome-borne curli shift gut bacterial populations and accelerate disease in a mouse model of Parkinson's disease

Graphical abstract

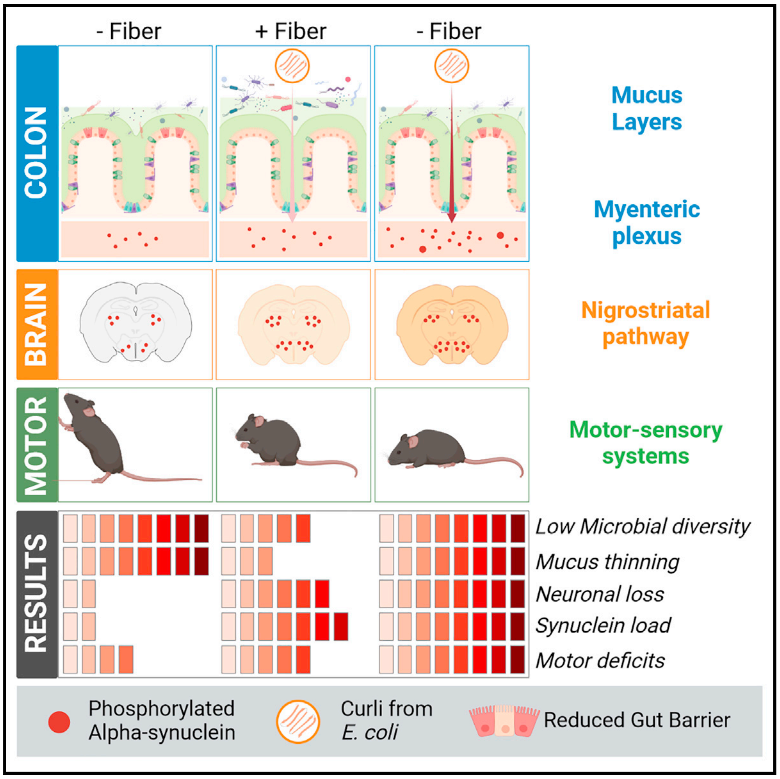

Highlights

- Fiber deprivation shifts microbiome structure and affects gut barrier
- Curli accelerates alpha-synuclein pathology in colon and brain
- Combining challenges replicates and accelerates Parkinsonian features

Authors

Kristopher J. Schmit, Pierre Garcia, Alessia Sciortino, ..., Michel Mittelbronn, Manuel Buttini, Paul Wilmes

Correspondence

kristopher.john.schmit@med.uni-tuebingen.de (K.J.S.),
paul.wilmes@uni.lu (P.W.)

In brief

Schmit et al. investigate how dietary fiber deprivation in combination with a bacterial toxin affect PD progression. They point to a translational PD-relevant sequence of events exacerbating disease progression in a disease mouse model and put forth the idea of lifestyle adaptations to mitigate the disease.

Schmit et al., 2023, Cell Reports *42*, 113071
September 26, 2023 © 2023 The Authors.
https://doi.org/10.1016/j.celrep.2023.113071

CellPress

CellPress
OPEN ACCESS

Article

Fiber deprivation and microbiome-borne curli shift gut bacterial populations and accelerate disease in a mouse model of Parkinson's disease

Kristopher J. Schmit,[1,2,3,9,*] Pierre Garcia,[1,3,9] Alessia Sciortino,[1,3] Velma T.E. Aho,[1] Beatriz Pardo Rodriguez,[1,3] Mélanie H. Thomas,[1,3] Jean-Jacques Gérardy,[3,4] Irati Bastero Acha,[1,3] Rashi Halder,[1] Camille Cialini,[3,5] Tony Heurtaux,[1,3,6] Irina Ostahi,[4] Susheel B. Busi,[1] Léa Grandmougin,[1] Tuesday Lowndes,[1] Yogesh Singh,[2] Eric C. Martens,[7] Michel Mittelbronn,[1,3,4,5,8] Manuel Buttini,[1,3,9] and Paul Wilmes[1,8,10,*]

[1]Luxembourg Centre for Systems Biomedicine, University of Luxembourg, 4362 Esch-sur-Alzette, Luxembourg
[2]Institute for Medical Genetics and Applied Genomics, Hospital University Tubingen, 72076 Tubingen, Germany
[3]Luxembourg Center of Neuropathology, 3555 Dudelange, Luxembourg
[4]National Center of Pathology, Laboratoire National de Santé, 3555 Dudelange, Luxembourg
[5]Department of Cancer Research, Luxembourg Institute of Health, 1526 Luxembourg, Luxembourg
[6]Department of Life Sciences and Medicine, University of Luxembourg, 4362 Esch-sur-Alzette, Luxembourg
[7]Department of Microbiology & Immunology, University of Michigan Medical School, Ann Arbor, MI 48109, USA
[8]Faculty of Science, Technology and Medicine, University of Luxembourg, 4365 Esch-sur-Alzette, Luxembourg
[9]These authors contributed equally
[10]Lead contact
*Correspondence: kristopher.john.schmit@med.uni-tuebingen.de (K.J.S.), paul.wilmes@uni.lu (P.W.)
https://doi.org/10.1016/j.celrep.2023.113071

SUMMARY

Parkinson's disease (PD) is a neurological disorder characterized by motor dysfunction, dopaminergic neuron loss, and alpha-synuclein (αSyn) inclusions. Many PD risk factors are known, but those affecting disease progression are not. Lifestyle and microbial dysbiosis are candidates in this context. Diet-driven gut dysbiosis and reduced barrier function may increase exposure of enteric neurons to toxins. Here, we study whether fiber deprivation and exposure to bacterial curli, a protein cross-seeding with αSyn, individually or together, exacerbate disease in the enteric and central nervous systems of a transgenic PD mouse model. We analyze the gut microbiome, motor behavior, and gastrointestinal and brain pathologies. We find that diet and bacterial curli alter the microbiome and exacerbate motor performance, as well as intestinal and brain pathologies, but to different extents. Our results shed important insights on how diet and microbiome-borne insults modulate PD progression via the gut-brain axis and have implications for lifestyle management of PD.

INTRODUCTION

Lifestyle and environmental factors contribute to chronic neurodegenerative diseases, burdening socio-economic structures in an ever-aging population.[1,2] The incidence of Parkinson's disease (PD), the second most common neurodegenerative disease, has increased over the last decades[3] and will increase further.[4] Only 5%–10% of cases are familial.[5] Because of the protracted nature of this disease, years lived with disabilities pose a significant burden on patients, their families, and society.[6] Genetic predispositions and environmental/lifestyle factors contribute to PD onset and progression.[7] Such disease-promoting factors are exposure to chemicals (e.g., pesticides), head trauma, physical activity, stress, and diet.[8] Which factors modulate disease progression has only recently been investigated.[9] Increasing evidence has highlighted the importance of diet in PD progression. A fiber-rich Mediterranean diet (fresh fruits and vegetables) reduces the risk for PD.[10] Conversely, a "Western" diet, with low amounts of fiber and high amounts of saturated fats and simple carbohydrates, increases risk for different diseases,[11,12] and in the case of PD, it accelerates progression.[13]

How diet contributes to disease starts to be understood.[9] Western diet has led to reduced gene expression related to complex carbohydrate fermentation.[14] In mouse models, low-fiber or fiber-free diets led to lower abundance of fiber-fermenting bacteria[15] and higher abundance of mucus-foraging species like *Akkermansia muciniphila*.[16] *Akkermansia* plays opposing roles in inflammatory (e.g., type 2 diabetes, atherosclerosis) versus neurological diseases.[17] Increased levels of *A. muciniphila* were consistently found in patients with PD[18] and were strongly associated with mucus erosion in patients[18] and mice.[16]

Pathogens typically originate from infectious agents[19] but also from commensal bacteria of the gut microbiome.[20] Commensal bacteria occupy the outer mucus layer of the colon and can form biofilms.[21,22] Even though there is no consensus on biofilm formation in the healthy gut,[23,24] it is observed in gastrointestinal diseases. Prominent biofilm-forming species are *Escherichia coli*

© 2023 The Authors.
This is an open access article under the CC BY license (http://creativecommons.org/licenses/by/4.0/).

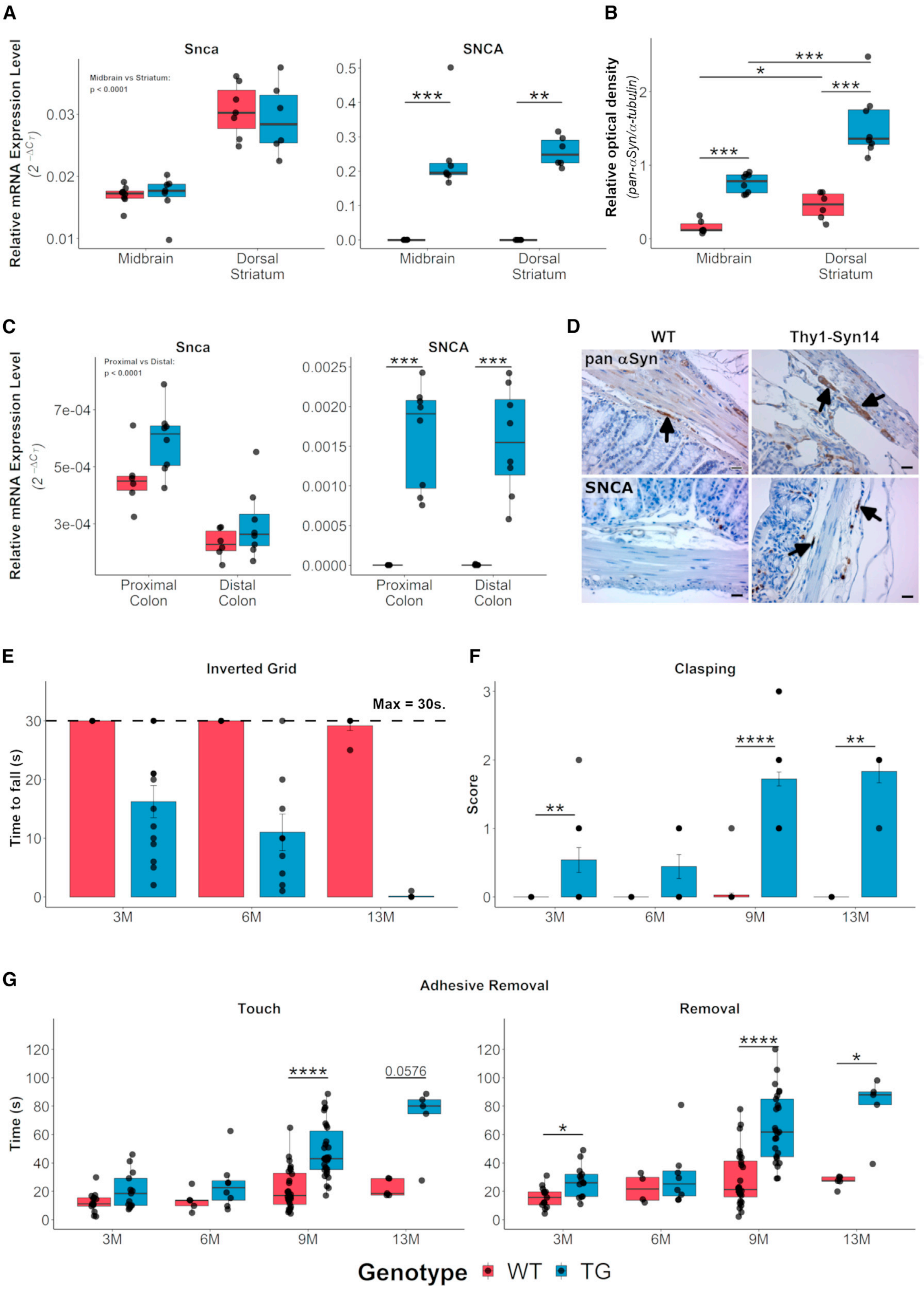

(legend on next page)

CellPress
OPEN ACCESS

(*E. coli*) and *Salmonella typhimurium*, which both express curli, a major biofilm component.[20] Curli is an amyloidogenic protein that has similarities to β-amyloid and α-synuclein (αSyn),[25,26] and it cross-seeds with αSyn.[27,28] αSyn aggregates are present in the gut of patients with PD.[29–32] Braak and colleagues proposed that αSyn aggregates in the enteric nervous system (ENS) precede those in the lower brainstem of the central nervous system (CNS), and that αSyn propagates retrogradely in a “prion-like” manner, via the vagus nerve, to and throughout the brain.[33]

Because environmental factors modulating disease act in concert, we investigated the effect of dietary fiber deprivation and bacterial curli exposure, individually or combined, on a human αSyn-overexpressing transgenic mouse. Our treatment protocol primed the naive untreated microbiome with a “Westernized” fiber-deprived diet,[16] followed by exposure to curli-producing bacteria.[27] We analyzed the mice at gut microbial, behavioral, gastrointestinal, and neuropathological levels. Overall, transgenic, but not wild-type mice, were susceptible to the different challenges. In transgenic mice, we found that, while αSyn overexpression was largely responsible for motor impairments, fiber deprivation caused dysbiosis, with increased levels of *Akkermansia* spp. and *Bacteroides* spp., sparking mucus erosion, facilitating entry of enteric pathogens.[16] This resulted in increased PD-like pathologies, including increased motor dysfunction, αSyn aggregation in the ENS and CNS, and nigrostriatal degeneration. We believe that our study sheds light on how a combination of internal and external pathogenic factors drives PD-like pathologies in the CNS and ENS. Our findings may have important implications for lifestyle adjustments that could mitigate PD progression.

RESULTS

Thy1-Syn14 mice overexpressed αSyn in brain and gut with regional differences

The model we used overexpresses human wild-type αSyn under the transcriptional control of the Thy1 promotor, and it carries 13 copies of the transgene.[34] Only protein levels in bulk brain tissue have been reported.[34] Thus, we first determined baseline gene expression and protein levels of αSyn in the nigrostriatal pathway and in the colon of naive 9-month-old mice. We hypothesized that, at this age, the mice would already have developed PD-like pathology (e.g., behavioral deficits, described below).

In our mice, mRNA level for the murine αSyn (*Snca*) gene (Figure 1A, left panel) did not differ between genotypes, but it was significantly ($p < 0.0001$) higher in the dorsal striatum versus the ventral midbrain. For the human αSyn transgene (*SNCA*) (Figure 1A, right panel), we only detected a signal in transgenic (TG) mice, and those levels did not differ between the two regions. When we stained for total αSyn, using an antibody (Figure S1A) that detects both murine and human αSyn, we saw that αSyn was present in both wild-type (WT) and TG mice in the dorsal striatum as well as in the substantia nigra pars compacta (SNpc). By western blot with that same antibody, we observed a 2.91-fold increase ($p = 6.67E-4$) in the dorsal striatum and a 6.67-fold increase ($p = 6.67E-4$) in the ventral midbrain of TG compared to WT mice (Figures 1B and S1B). Additionally, αSyn protein levels were significantly higher in the dorsal striatum compared to the ventral midbrain (WT: $p = 1.52E-2$; TG: $p = 1.55E-4$; Figures 1B and S1B).

In the colon, which we split into proximal and distal parts, we observed that the mRNA levels for *Snca* and *SNCA* were much lower compared to those in the CNS (Figure 1C). A possible explanation could be the lower neuronal density in colon compared to brain. In the colon, only about 1% of all cells are neurons,[35] whereas ventral structures of the midbrain (e.g., SNpc and ventral tegmental area) have an estimated 15%–20% neurons.[36–39] Nevertheless, we observed that again *SNCA* was only expressed in TG mice, and *Snca* was expressed at significantly ($p < 0.0001$) different levels between proximal and distal colonic regions but not between genotypes (Figure 1C). Additionally, we stained for human αSyn and total αSyn (Figure 1D). The latter was expressed in mice of both genotypes, while only TG mice expressed human αSyn (Figure 1D, left bottom panel).

Based on our analysis, the Thy1-Syn14 model is an appropriate system to investigate the effect of environmental challenges delivered through the gut in the context of αSyn overexpression.

Thy1-Syn14 mice exhibited progressive motor deficits

PD-like motor deficits were reported in different mouse models of the disease.[40] We assessed grip strength, hindlimb reflexes, and coordination/movement as measures of motor dysfunction in naive cohorts of 3-, 6-, and 13-month (M)-old mice. For hindlimb reflexes and coordination/movement assessments, we added

Figure 1. Thy1-Syn14 mice had pronounced alpha-synuclein and motor impairment phenotypes

(A) Relative mRNA expression levels for *Snca* (left) do not differ between Thy1-Syn14 (TG, blue) and wild-type (WT, red) mice but are significantly different between ventral midbrain and dorsal striatum. *SNCA* (right) is only expressed in TG mice and at identical levels in regions. Mice were 9 months old. Normalized to *Gapdh*. Sample size: WT, n = 8; TG, n = 7.

(B) Relative densities from western blots for total αSyn (pan-αSyn) in the ventral midbrain and dorsal striatum. Mice were 9 months old. Normalized to α-tubulin. See also Figure S1B. Sample size: WT, n = 6; TG, n = 8.

(C) Relative mRNA expression levels for *Snca* (left) do not significantly differ between TG and WT mice but are significantly different between proximal and distal colon regions. *SNCA* (right) is only expressed in TG mice at identical levels between regions. Mice were 9 months old. Normalized to *Gapdh*. Sample size: WT, n = 6; TG, n = 8.

(D) Representative micrographs from immunohistochemical stainings showing that SNCA is detected only in enteric neurons (arrow, right lower panel) of TG animals, while pan-αSyn (top row panel, black arrows) is expressed in genotypes. Scale bar: 25 μm.

(E–G) Mice were tested at different ages for gross and fine motor skills by (E) inverted grid, (F) hindlimb clasping, and (G) adhesive removal. All tests show an age-dependent increase in motor deficits in TG mice. Each age group was a separate cohort. Baseline results at 9 months (M) served for the selection of this age for the challenge study cohort. Sample size: 3M WT, n = 13; TG, n = 13; 6M WT, n = 7; TG, n = 9; 9M WT, n = 35; TG, n = 37; and 13M WT, n = 6; TG, n = 6. Results were analyzed by Mann-Whitney U, corrected for FDR; *FDR < 0.05, **FDR < 0.01, ***FDR < 0.001, ****FDR < 0.0001. See Table S2.

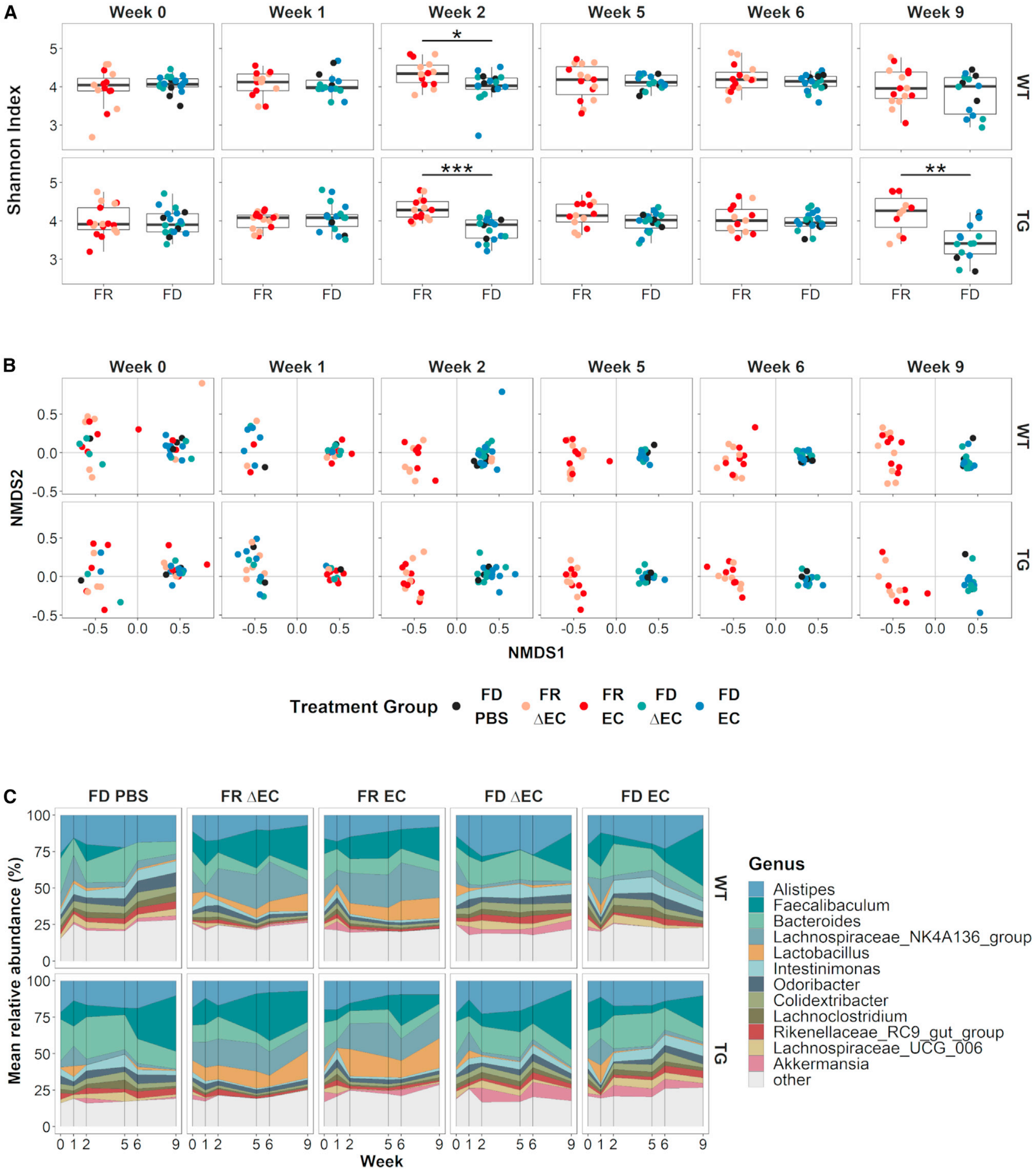

Figure 2. Longitudinal changes in microbial diversity and composition shifts were diet driven

(A) Boxplots illustrating alpha diversity for the two diets over time; both genotypes are shown separately. Low amount of dietary fiber (FD) reduced microbial diversity significantly at week 2 in WT ($p < 0.05$) and TG ($p < 0.001$) mice. At week 2, we started gavages with *E. coli*. In TG mice, microbial diversity was again significantly ($p < 0.01$) reduced at week 9. See also Figure S2A. Results were analyzed by Mann-Whitney U corrected for FDR; *FDR < 0.05; **FDR < 0.01; ***FDR < 0.001.

(legend continued on next page)

the baseline results from our treatment cohort at 9 months of age (see below).

For grip strength, we measured the hanging time to assess the simultaneous four-limb grip strength. Grip strength gradually decreased with age in TG mice (3M: 16.2 ± 9.88 s, p = 3E−4; 6M: 11 ± 9.39 s, p = 3E−4; 13M: 0.17 ± 0.41 s, p = 4.86E−7; Figure 1E), while it remained unchanged in WT littermates.

The hindlimb reflexes confirmed that the performance of TG mice decreased with age (3M: p = 9.33E−3; 6M: p = 5.7E−2; 9M: p < 0.0001; 13M: p = 4E−3; Figure 1F), while WT littermates remained unimpaired.

Finally, we tested mice for fine motor skills with the adhesive removal test. We did not observe relevant motor deficits in young mice (3M and 6M; Figure 1G). At 9 and 13 months, we saw that both sensitivity (time at touch, left panel) as well as coordination (time at removal, right panel) were significantly delayed in TG mice (time at touch – 9M: p = 7.57E−7, 13M: p = 0.057; time at Removal – 3M: p = 0.016, 9M: p = 1.12E−6, 13M: p = 0.016).

Taken together, these data indicate that overexpression of human αSyn drives progressive motor dysfunction in Thy1-Syn14 mice.

Fiber deprivation induced PD-related microbiome changes

Onset and progression of PD have been linked to environmental factors,[41–44] of which some, e.g., diet, impact the gut microbiome.[45,46] Changes in gut microbial composition have been described in patients with PD[47–53] and in animal models.[54–56] It is unknown how these changes affect the progression of PD. Using 16S rRNA gene amplicon sequencing, our goal was to first understand how the exogenous challenges affected, independently or in combination, the gut microbiome in our mice. The exogenous challenges were as follows:

(1) Diet: a normal fiber-rich (FR) or a fiber-deprived (FD) diet
(2) Gavages: PBS, curli knockout isogenic *E. coli* (ΔEC), or curli-producing *E. coli* (EC)

We started by looking at how the challenges affected microbial diversity. We saw that the FD diet and TG challenges both reduced inner-group diversity (alpha diversity) but that the gavage challenges, PBS or *E. coli* (EC or ΔEC) (Figure S2A), did not. Alpha diversity was lowest at week 2 (WT: p = 0.045; TG: p = 6.1E−4) and week 9 (TG: p = 9.7E−3) in FD-challenged and more strongly so in TG mice (Figure 2A). We did not observe this microbial shift at weeks 5 and 6. A possible explanation is that the initial treatment with *E. coli* at week 2 caused a transitory disruption in the microbial balance. We made similar observations for beta diversity, where the diet challenge was the main driver (adonis p = 0.001) of dissimilarities between groups (Figure S2B, middle panel). However, in contrast to alpha diversity, the gavage challenges, PBS or *E. coli* (EC or ΔEC), also significantly (adonis p = 0.001) contributed to microbial community shifts (Figure S2B, right panel). This might have been due to the PBS-gavaged mice having all been FD challenged. Hence, solely the FD challenge drove the observed rapid shift in microbiome (Figure 2B). Already at week 2, the FD and FR groups, independent of the other challenges, formed two homogeneous clusters, which, unlike the alpha diversity results, lasted until the end of the in-life phase. Such a shift is indicative of dysbiosis. Changes in the *Firmicutes* to *Bacteroidetes* ratio, formerly a standard for microbial imbalance,[57,58] showed significantly higher levels of *Firmicutes* compared to *Bacteroidetes* in FD-challenged mice at the end of the in-life phase (week 9, Figure S3).

Abundance differences on the genus level were again driven though the FD diet challenge (Figure S4A). When we focused on our most relevant genera (Figure 2C), we observed that the FD challenge led to higher levels in *Intestinimonas, Odoribacter, Colidextribacter, Rikenellaceae RC9 gut group* and *Akkermansia* and decreased levels in *Lachnospiraceae NK4A136 group* and *Lactobacillus* over time (Figures 2C and S4A). Other taxa presented with a more fluctuating behavior over time (Figures 2C and S4A). When compared to data from patients with PD (Table 3 in Boertien et al.[47]), FD-challenged mice showed similar changes in *Akkermansia*, *Lachnospiraceae*, *Roseburia*, and *Prevotellaceae*, while *Lactobacillaceae* and its genus *Lactobacillus* were inversely altered (Table S1).

When we checked for the relative abundance of *E. coli*, the species we gavaged with, we did not detect it in our 16S rRNA gene amplicon sequencing data (not shown). We performed a probe-based quantitative real-time PCR analysis, revealing that fecal *E. coli* levels were significantly (Kruskal-Wallis, p = 4.55E−12) increased in FD-diet-challenged mice compared to mice on the FR diet (Figure S4B), as well as being significantly (Mann-Whitney U test, false discovery rate [FDR] = 0.027) higher in mice gavaged with PBS compared to EC-challenged mice (Figure S4C) (independent of genotype and time points). A possible explanation for higher fecal *E. coli* levels in FD-diet-fed mice could be the inability of *E. coli* to implant in the eroded outer mucus layer but still sufficiently so as to have pathogenic effects (see below). Looking into how levels changed over time, starting when we first gavaged animals (week 2), we did see significantly higher levels of *E. coli* in FD-diet-fed compared to FR-diet-fed mice (Kruskal-Wallis; WT: week 5, p = 0.0366; TG: week 2, p = 0.00793; week 5, p = 0.0267; Figure S4D), but we did not observe incrementally increasing fecal *E. coli* levels. Similarly, in another study,[27] *E. coli* administration induced PD-like pathologies without colonizing the gut.

In summary, the FD challenge caused reduced gut microbial diversity with changes in taxa associated with gut health and disease. Of note were reduced levels of *Lactobacillus*, a probiotic genus,[59,60] reported to have neuroprotective[61] and gut-barrier-integrity-enhancing functions,[62] and of the *Lachnospiraceae*

(B) Non-metric multi-dimensional scaling (NMDS) representations of beta diversity for both genotypes (row) and the different time points (week, column). We observed a microbiome structure shift leading to two homogeneous clusters by week 2 induced by the FD diet challenge. See also Figure S2B.
(C) Temporal distribution of relative abundance for the 12 most abundant taxa at the genus level for weeks 0 (baseline), 1, 2, 5, 6, and 9. Both WT and TG mice showed similar changes in relative abundance for the different taxa. Differences were observed between the diet challenge groups. See also Figure S4A and Table S2.

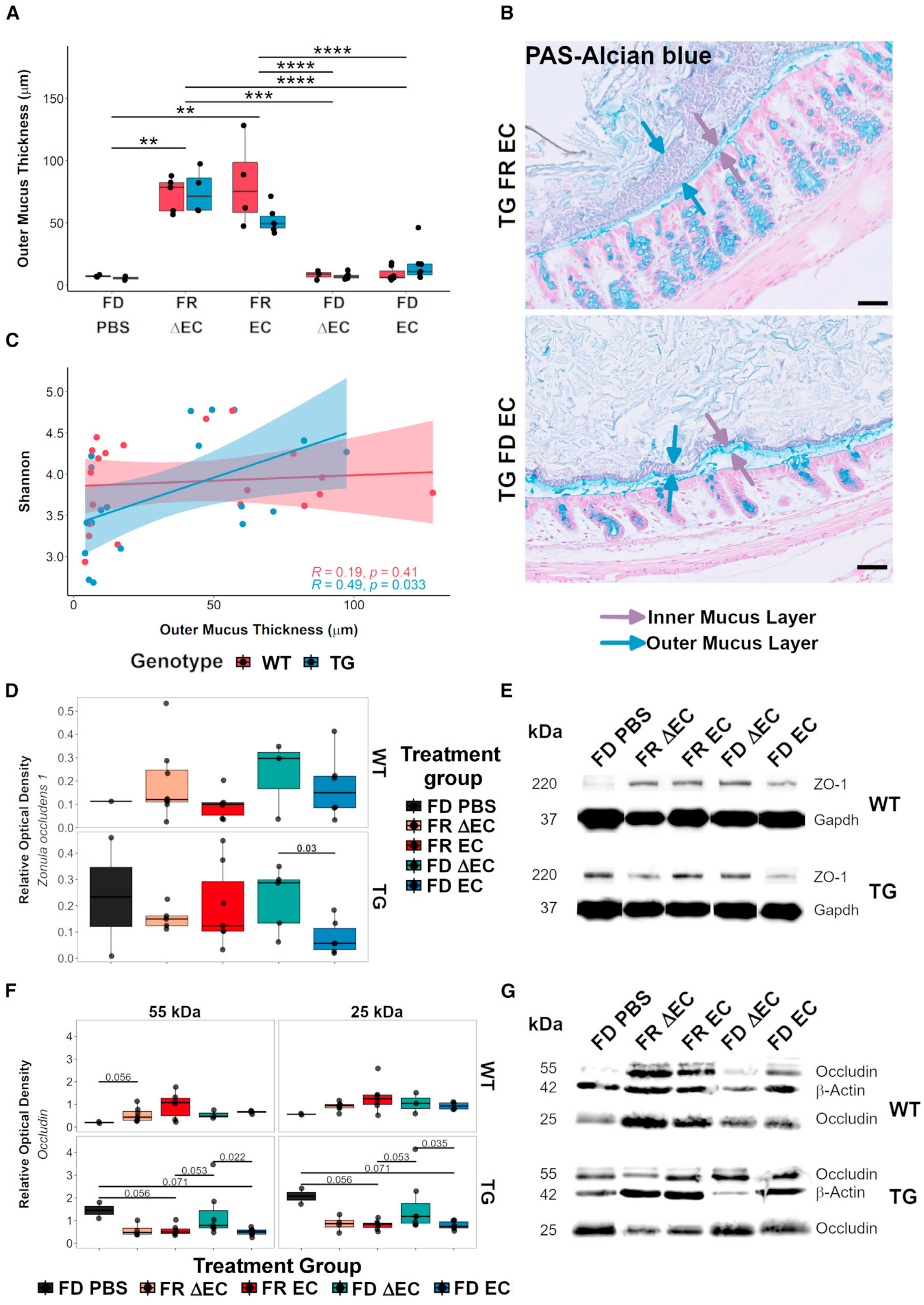

(legend on next page)

NK4A136 group, which inversely correlates with risk for PD or dementia.[63] Additionally, *Lachnospiraceae NK4A136 group* and *Roseburia* are important butyrate producers, promoting proper gut barrier function.[64] Together with higher levels of mucin-foraging genera *Akkermansia, Alistipes*, and *Bacteroides*,[16,65,66] the passage of pathogenic factors through the barrier might be increased.

Microbiome-driven colonic mucus erosion in fiber-deprived Thy1-Syn14 mice

The mucus layers of the colon have two basic functions: the inner layer acts as a physical barrier to prevent pathogens from reaching the gut epithelium, and the outer layer harbors commensal bacteria interacting with the host.[22] Increasing levels of mucin-degrading bacteria, such as *A. muciniphila* and certain *Alistipes* spp. and *Bacteroides* spp.,[16] cause mucus thinning and thus enable epithelial access for pathogens. In a pilot study (Figures S5A–S5C), where we fed an FD diet to TG mice and WT mice, we saw that, after 1 week, there was a rapid and significant (FDR = 0.004; Figure S5C) erosion of the outer mucus layer in FD-diet-fed mice, independent of their genotype. After 3 weeks, the outer mucus layer remained significantly (FDR: 0.036; Figure S5C) thinned in mice of both genotypes. The inner mucus layer did not differ between the different conditions (Figure S5B). This initial observation showed how rapidly the outer mucus erodes upon fiber deprivation.

In FD-challenged mice, similar to the pilot study, we did not detect changes of the inner mucus layer (Figures S5D and S5E). Our results for the outer mucus layer on the other hand clearly showed that the FD challenge caused significant ($p = 1.15E-12$) thinning of the outer mucus layer (Figure 3A). The layer thickness decreased extensively by 49.1%–92.9% in the FD diet group (Figure 3B). Hence, the habitat for specific gut bacteria was reduced, which we expected to directly affect microbial diversity. Therefore, we compared alpha diversity and mucus thickness using the Spearman's rank test (Figure S5F). We were specifically interested in dietary- or transgene-driven associations. While we only saw moderate dietary-driven correlations (FR: $r = -0.47$, $p = 0.051$; FD: $r = 0.38$, $p = 0.087$; Figures S5G and S5H), there was a significant positive correlation between alpha diversity and mucus thickness in TG mice (Figure 3C), indicating that the outer mucus was more susceptible to FD-induced thinning when αSyn was overexpressed.

Curli exposure and fiber deprivation disrupted gut barrier integrity in Thy1-Syn14 mice

Our microbiome data indicated changes in gut-barrier-integrity-relevant taxa. To assess gut barrier integrity, we looked at two different tight junction proteins, zonula occludens-1 (ZO-1) and occluding, by western blot. These tight junction proteins, form, with others, a continuous barrier between epithelial cells that is disrupted by pathological conditions,[67–69] resulting in a "leaky gut," which has been described in patients with PD and other diseases.[70,71]

Interaction of genotype and diet was the main driver of differences in ZO-1, which not quite reached significance ($p = 0.0698$, by permutational multivariate analysis of variance). When we investigated group differences for each genotype, we saw that the FD EC challenge in TG mice had overall the lowest levels of ZO-1, with notable (Mann-Whitney U test, before correction; $p = 0.03$) lower levels than the FD ΔEC-challenged TG mice (Figures 3D and 3E).

For occludin, we detected a band at 55 kDa (up to 65 kDa; referred to as 55 kDa here) and another band at 25 kDa in our gels (Figure 3G). This smaller isoform is most likely due to alternative gene splicing[72,73] and has been observed in another study.[74] The 25-kDa isoform levels were significantly higher in both WT (Kruskal-Wallis, $p = 0.00335$) and TG (Kruskal-Wallis, $p = 0.00483$) mice compared to the 55-kDa form. We analyzed the datasets for the 55-kDa and the 25-kDa occludin isoforms separately (Figure 3F). For both, the genotype did not explain group differences. Thus, we analyzed each genotype separately. While in WT mice, occludin levels were quite variable, in TG mice, we saw significantly (Mann-Whitney U test before correction for multi-comparison) lower levels of 55-kDa ($p = 0.022$) and 25-kDa ($p = 0.035$) occludin, respectively, after the FD EC compared to the FD ΔEC challenges (Figure 3F). Thus, the level of occludin was lowered by all treatments in TG mice compared to the FD PBS control group, indicating that occludin is a sensitive marker for gut barrier dysfunction (Figure 3F).

We can conclude that the EC challenge alone affects the gut barrier integrity, but the effect is most pronounced when combined with the transgene and the fiber deprivation challenges.

Figure 3. Fiber deprivation acting with curli induced gut mucus erosion and barrier leakiness

(A) Boxplot of outer mucus thickness measurements. The FD challenge caused a strong reduction in outer mucus thickness. Results were analyzed by Mann-Whitney U test, corrected for FDR; **FDR < 0.01; ***FDR < 0.001; ****FDR < 0.0001. Sample sizes: WT FD PBS, n = 3; WT FR ΔEC, n = 5; WT FR EC, n = 4; WT FD ΔEC, n = 3; WT FD EC, n = 7; TG FD PBS, n = 2; TG FR ΔEC, n = 4; TG FR EC, n = 6; TG FD ΔEC, n = 6; and TG FD EC, n = 7

(B) Representative micrographs illustrating the differences in outer mucus erosion between mice challenged with FR (top) and FD diet (bottom).

(C) Scatterplot of a Spearman rank test comparing alpha and mucus thickness for both genotypes separately, revealing a significant positive correlation between microbial diversity and mucus thickness in Thy1-Syn14 mice. See also Figure S5.

(D and E) Boxplots illustrating protein levels of zonula occludens (ZO-1) (D) and representative western blot gel images (E). Relative optical density levels for ZO-1 (220kDa) were normalized against Gapdh (57 kDa). In TG mice, we saw that the FD EC challenge resulted in significantly ($p = 0.03$) lower ZO-1 levels compared to FD ΔEC-challenged mice. Sample size: WT FD PBS, n = 1; WT FR ΔEC, n = 8; WT FR EC, n = 6; WT FD ΔEC, n = 3; WT FD EC, n = 6; TG FD PBS, n = 2; TG FR ΔEC, n = 5; TG FR EC, n = 7; TG FD ΔEC, n = 5; and TG FD EC, n = 6.

(F and G) Boxplots illustrating protein levels of colonic occludin (F) and representative western blot gel images showing both molecular weight isoforms of occluding (G). Relative optical density levels for both occludin fractions were normalized against β-Actin (42 kDa). Particularly in TG mice, we saw significantly lower levels for both occludin fractions (55 kDa: $p = 0.022$; 25 kDa: $p = 0.035$) in FD EC- compared to FD ΔEC-challenged mice. Results were analyzed by Mann-Whitney U test, not corrected for FDR. Sample sizes: WT FD PBS, n = 2; WT FR ΔEC, n = 7; WT FR EC, n = 6; WT FD ΔEC, n = 3; WT FD EC, n = 4; TG FD PBS, n = 2; TG FR ΔEC, n = 4; TG FR EC, n = 7; TG FD ΔEC, n = 7; and TG FD EC, n = 6. See Table S2.

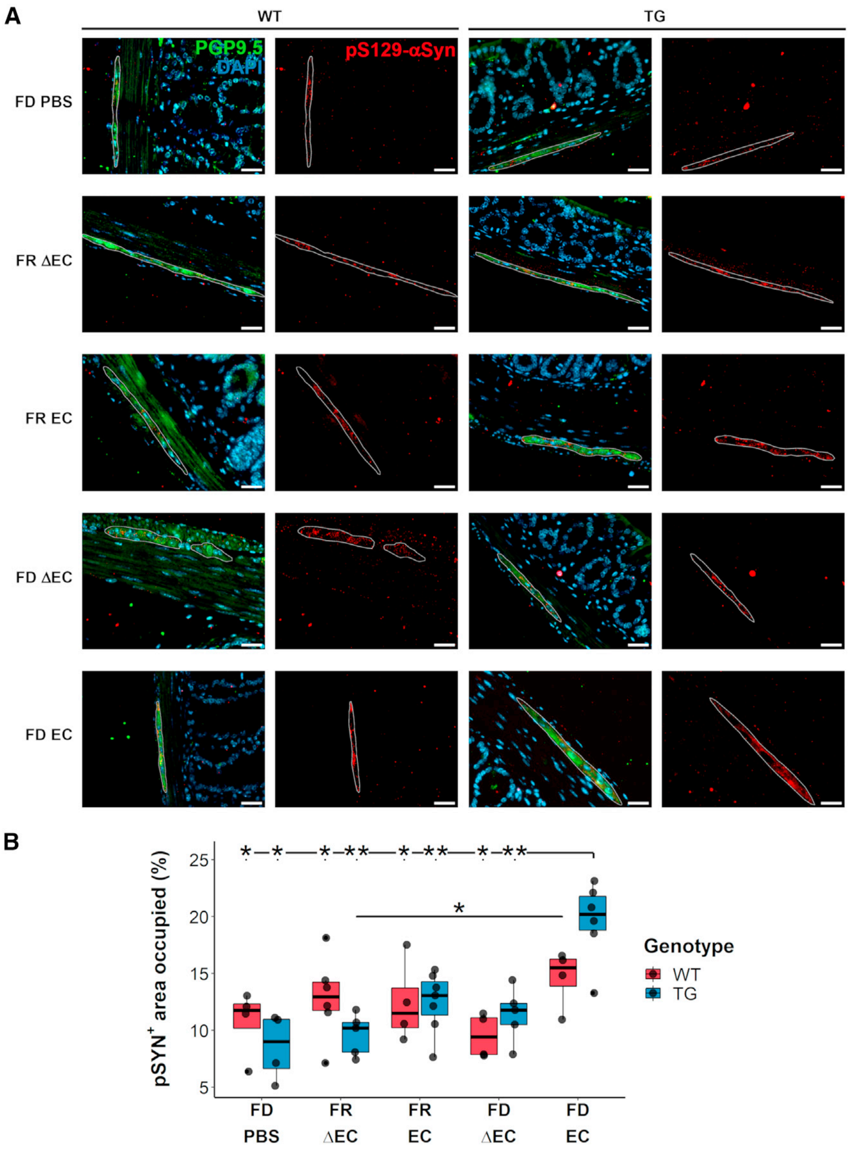

Figure 4. Curli-driven phosphorylated alpha-synuclein aggregate formation in fiber-deprived Thy1-Syn14 mice

(A) Representative microphotographs of pS129-αSyn-positive structure containing ganglions in the myenteric plexus of WT and TG (left to right) mice of any treatment group (FD PBS to FD EC from top to bottom). Qualitatively, in TG mice, pS129-αSyn-positive structures after FD EC challenges seemed larger and more numerous. Scale bar: 50 μm.

(B) Boxplot illustrating area occupied changes by pS129-αSyn-positive structures in ganglions of the myenteric plexus. The most significant area increase of pS129-αSyn-positive structures was seen in TG FD EC-challenged mice. Results were analyzed by Mann-Whitney U test, not corrected for FDR. Sample sizes: WT FD PBS, n = 4; WT FR ΔEC, n = 6; WT FR EC, n = 4; WT FD ΔEC, n = 4; WT FD EC, n = 4; TG FD PBS, n = 4; TG FR ΔEC, n = 5; TG FR EC, n = 7; TG FD ΔEC, n = 5; and TG FD EC, n = 6. See Table S2.

Bacterial curli increased pS129-αSyn-positive structures in the colonic myenteric plexus of fiber-deprived Thy1-Syn14 mice

αSyn aggregation in the gut is detected in patients with PD.[33,75,76] To test for αSyn aggregation in the gut of our mice, we used an antibody directed against phosphorylated Ser129 αSyn (pS129-αSyn). This type of antibody is commonly used to detect αSyn aggregation in murine and human CNS[77] and ENS.[78,79] We quantified pS129-αSyn-positive deposits in ganglions of the myenteric plexus positive for protein gene product 9.5 (PGP9.5), a neuronal cytoplasmic marker.[80] Other pS129-αSyn-positive structures in the submucosal plexus and submucosa were irregular and mainly detected in TG mice. This, however, did not differ between the different treatment groups, and we could not determine the cell types involved.

First, the staining showed that both WT and TG mice had pS129-αSyn-positive signals in ganglions of the myenteric plexus (micrographs in Figure 4A). Quantification of these signals showed that only TG mice exposed to the combined challenges of FD and curli-expressing *E. coli* (FD EC) had increased levels of pS129-αSyn in PGP9.5-positive ganglions (vs. TG FD PBS: $p = 0.019$; vs. WT FR EC: $p = 0.019$; vs. TG FR EC: $p = 0.008$; vs. WT FD ΔEC: $p = 0.019$; Figure 4B).

In summary, the strongest increase of pS129-αSyn-positive structures occurred in the myenteric plexus of the colon of TG EC FD-challenged mice.

Bacterial curli and dietary fiber deprivation combination exacerbated motor deficits in Thy1-Syn14 mice

At baseline, in 9-month-old TG mice, we did already observe motor deficits. Therefore, we examined if the challenges, individually or in combination, exacerbated these deficits.

Three different tests were chosen to assess motor performance: hindlimb clasping, grip strength, and adhesive removal. At baseline, TG mice differed significantly from WT littermates in the first two tests by showing age-related decline in performance (Figures 1F, S6A, and S6B). These declines were not affected by the other challenges and thus most likely driven by αSyn overexpression.

The adhesive removal test was used to assess fine motor function. The test consists of measuring the latencies of touch and removal, which at baseline were significantly ($p < 0.0001$) greater in TG mice compared to WT littermates (Figure 1G). This was still the case at the end of the 9-week challenge phase ($p < 0.0001$; Figures 5A and S6C). Hence, αSyn overexpression was again key in driving motor impairment. Additionally, EC-challenged TG mice, independent of diet, showed increased latencies of touch

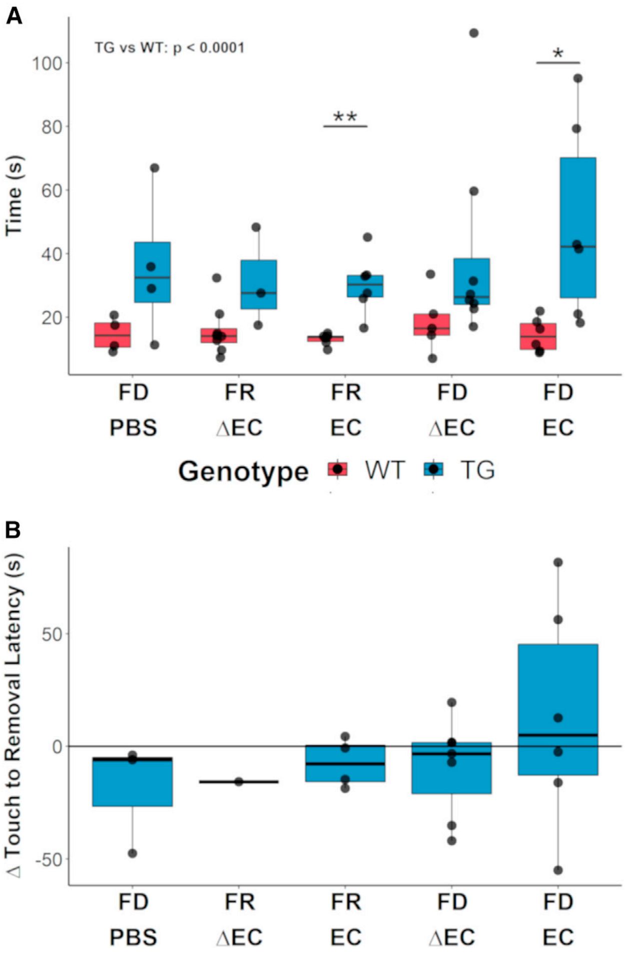

Figure 5. Challenges exacerbated motor impairment in a subset of Thy1-Syn14 mice

(A) Boxplots illustrating the latency for removal in the different treatment groups (x axis) for WT (red) and TG (blue) mice after 9 weeks. The results shown are for the first paw and the first replicate. TG animals took significantly (Kruskal-Wallis, $p < 0.0001$) longer than WT animals to remove the tape. On a group-by-group basis, TG mice took significantly longer than WT mice for the FR EC and the FD EC challenge groups. Results were analyzed by Mann-Whitney U test, not corrected for FDR; *$p < 0.05$; **$p < 0.01$. Sample sizes: WT FD PBS, n = 4; WT FR ΔEC, n = 8; WT FR EC, n = 6; WT FD ΔEC, n = 5; WT FD EC, n = 6; TG FD PBS, n = 4; TG FR ΔEC, n = 5; TG FR EC, n = 7; TG FD ΔEC, n = 8; and TG FD EC, n = 7.

(B) Summary plot illustrating how the adhesive removal performance evolved from baseline to endpoint in TG mice, plotting the time difference from touch to removal. Only the FD EC group shows a performance drop for three out of six mice. Note: for the TG FR ΔEC group, only one mouse performed normally and is therefore not representative. Sample sizes: FD PBS, n = 3; FR ΔEC, n = 1; FR EC, n = 5; FD ΔEC, n = 6; and FD EC, n = 6. See Table S2.

and removal compared to WT mice (Figures 5A and S6C). Further, we saw a noticeable increase in both measures for a subset of TG FD EC-challenged mice. To understand how the ability to remove the adhesive tape changed over time, we (1) subtracted the time of touch from the time of removal and then (2) compared "baseline" to "endpoint" results. We found that, in the TG FD EC group, three out of six mice had reduced coordinative ability to remove the adhesive tape compared to their initial performance (Figure 5B). Thus, after grouping mice into categories, our results indicate that the combination of the FD diet and curli exacerbated the motor phenotype in a subset of TG mice.

When we compared these results to our baseline assessment of 13-month-old untreated transgenic mice, there was no significant difference. We believe that this could indicate that severity of motor deficits may already have plateaued. They may be due to expression of αSyn in the spinal cord as has been described in models using the Thy1 promotor.[81] Only the further increase picked up in the sensitive adhesive removal test after FD and EC challenge may be due to the loss of striatal TH-positive axons and nigral TH-positive neurons (see below).

Bacterial curli increased pS129-αSyn-positive structures in the nigrostriatal pathway of Thy1-Syn14 mice

All neuropathological analyzes were limited to TG mice, since they had pre-existing PD pathologies and were susceptible to the challenges. In a first step, we determined abnormal αSyn structures in the nigrostriatal pathway. We quantified pS129-αSyn-positive structures in the dorsal striatum and the SNpc. We observed that the EC challenge was the main driver of enhanced pS129-αSyn aggregation in both regions (Figure 6).

In the dorsal striatum, we measured significantly ($p = 0.018$) higher levels of pS129-αSyn-positive structures in FR EC-challenged TG mice compared to the FR ΔEC group (Figure 6A, top panel), a difference that was obvious upon viewing (Figure 6A, lower panels). In the SNpc, we saw that the impact of the FD challenge was greater than in the dorsal striatum (Figure 6A). Both pS129-αSyn-positive cell body counts and pS129-αSyn-positive structures with neurite-like morphology were increased the most in FD EC-challenged mice ("Cell Body," vs. FR ΔEC: $p = 0.127$; "Other," vs. FD PBS: $p = 0.109$, vs. FR ΔEC: $p = 0.019$, vs. FR EC: $p = 0.072$, vs. FD ΔEC: $p = 0.13$, Figure 6B, top panel). Qualitatively, pS129-αSyn-positive structures observed in cell bodies appeared diffuse in the cytoplasm but more compact in the nuclei (Figure 6B, bottom panel, white arrowhead). Importantly, nuclear αSyn aggregation has been demonstrated in brains of patients with Lewy Boyd dementia.[82] However, in FD EC-challenged mice, we also found dense pS129-αSyn-positive deposits in cell bodies (Figure 6B, bottom panel, blue arrowheads). All other pS129-αSyn-positive structures that were not within cell bodies were of three different types:

- Bead-like varicosities (Figure 6B, bottom panel, white arrow), similar to what has been observed in other *in vivo*[83] and *in vitro*[84] models and human postmortem brains[85]
- Spheroid-shaped structures (Figure 6B, bottom panel, open arrow)
- Corkscrew-like spheroid structures (Figure 6B, bottom panel, blue arrow), rare and only observed in FD EC-challenged TG mice

To test if the pS129-αSyn-positive structures observed after challenges were composed of beta-pleated sheets, we performed consecutive immunostaining for pS129-αSyn and

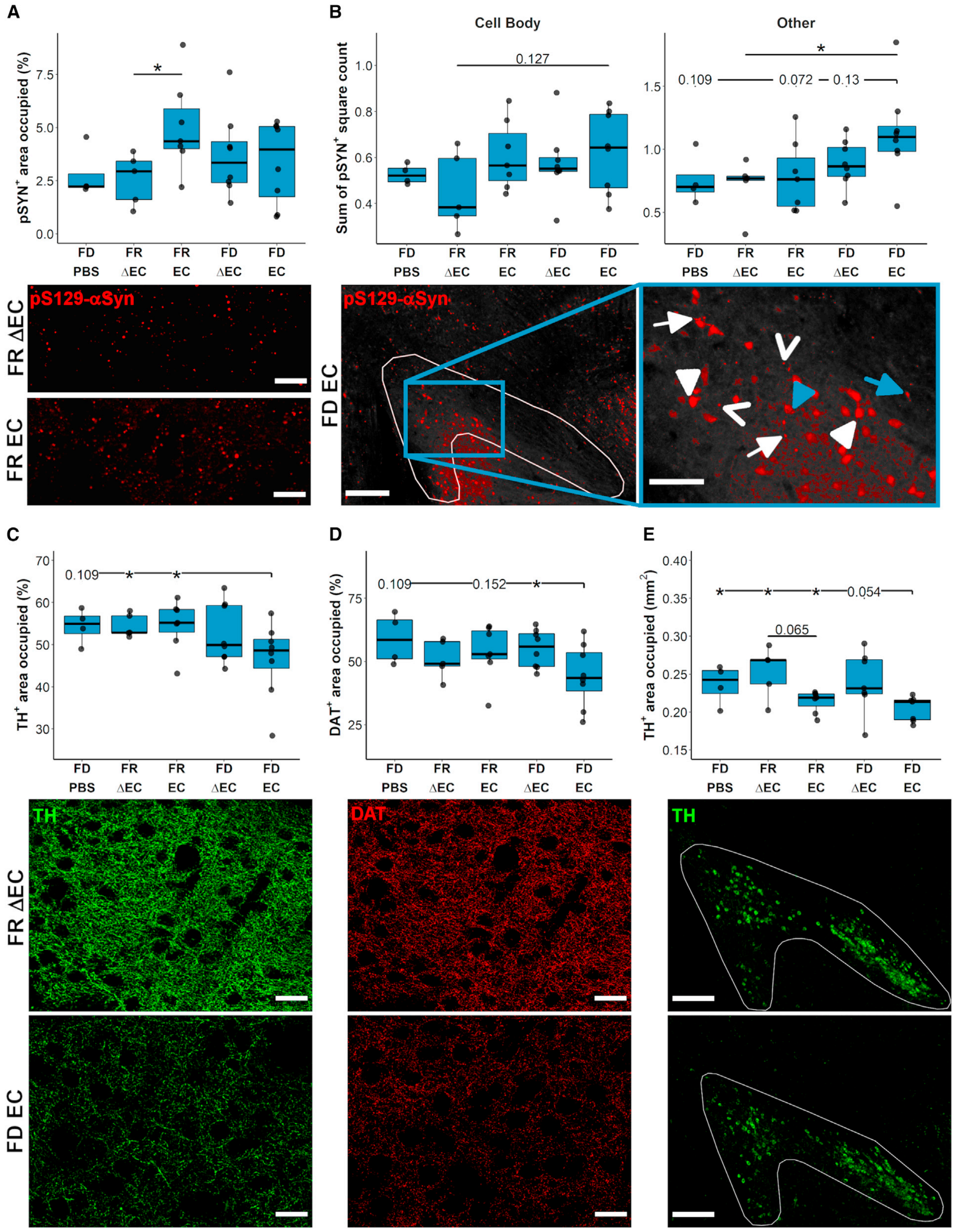

(legend on next page)

CellPress
OPEN ACCESS

thioflavin-S (Thio-S) on select sections from challenged TG mice. None of the pS129-αSyn-positive structures (Figure S7; first two rows) stained positive for Thio-S. This observation was similar to the one we made in the αSyn-preformed fibril injection model (Figure S7; second row), where intracellular inclusions in the SNpc (Figure S7; third row) and dorsal striatum (not shown) were also Thio-S negative, despite significant loss of TH-positive neurons in the SNpc and their striatal projections.[86] This indicates that Thio-S-positive inclusions are not a necessary feature of PD-like neurodegeneration. To ensure that our consecutive immuno-/Thio-S staining procedure detects pathologically aggregated protein deposits, we confirmed presence of doubly labeled deposits (amyloid beta and tau) in brain sections of two models of Alzheimer's disease (Figure S7; bottom two rows). Which form of misfolded or aggregated αSyn is the major pathological culprit in PD is still a matter of debate[87] and beyond the scope of this study.

In summary, it was primarily the EC challenge that increased pS129-αSyn aggregation, a process that was exacerbated by fiber deprivation.

Curli combined with dietary fiber deprivation drove neurodegeneration in the nigrostriatal pathway of Thy1-Syn14 mice

The loss of neurons in the SNpc and their projections to the dorsal striatum is a main pathological hallmark of PD.[5] To detect neurodegeneration in TG mice after the different challenges, we stained against tyrosine hydroxylase (TH), an enzyme involved in dopamine synthesis and a marker for dopaminergic neurons and their projections, in both the SNpc and dorsal striatum. Additionally, in the dorsal striatum, we stained for the dopamine transporter (DAT), a marker for dopamine-cycling synapses.

In the dorsal striatum, the combination of FD and EC challenges in TG mice resulted in significantly reduced TH-positive projections when compared to the FR ΔEC ($p = 0.029$) and FR EC ($p = 0.04$) groups (Figure 6C, top panel). For DAT, we observed a similar pattern (Figure 6D, top panel). The FD EC-challenged TG mice showed significantly reduced levels in DAT when compared to FD ΔEC ($p = 0.05$) and strong trends compared to the FD PBS and FR EC groups (Figure 6D, top panel).

Quantitation of TH-positive neurons (Figure 6E, top panel) showed that the exposure to curli caused significant neuronal loss (EC-PBS: FDR = 0.041; EC-ΔEC: FDR = 1.68E−4). FD mice showed an exacerbation of this loss. The difference between the FR EC and FD EC groups showed a strong ($p = 0.054$) trend, indicating that the FD challenge led to an exacerbation of curli-driven neurodegeneration and no evidence for central, and only minimal peripheral, inflammation in Thy1-Syn14 mice after fiber deprivation and/or curli exposure.

Central, but also peripheral, inflammation is an intrinsic part of PD pathogenesis.[88] To assess inflammation in the brain, we used staining for the microglial marker ionized calcium-binding adapter molecule 1 (Iba1). Surprisingly, we found no evidence for increased Iba1 staining in TG mice of any group compared to their WT or treatment controls (Figure S8A).

To assess inflammation in the periphery, we measured calprotectin in feces and a panel of chemokines and cytokines in plasma. Calprotectin is secreted into the intestinal lumen by immune and epithelial cells, and it is an inflammation marker for inflammatory bowel disease (IBD)[89,90] or PD.[46,91] We found a slight but significant increase of calprotectin over time (WT – week 2, $p = 0.025$; week 6, $p = 0.017$; post-mortem [PM], $p = 0.002$; TG – week 1, $p = 0.02$; week 2, $p = 0.003$; week 6, $p = 0.017$; PM, $p = 0.059$), driven by the FD diet challenge (Figure S8B). When compared to a colitis model,[92] these values were greatly lower, indicating that, in our model, gut inflammation was minimal.

Patients with PD have increased circulating inflammation markers.[88] Therefore, we tested plasma from a randomly selected subset of mice from each group against a panel of 40 chemo- and cytokines. Surprisingly, TG mice, no matter the challenges, had overall lower inflammation levels than WT mice (Figure S8C).

We believe these surprising observations can be explained by the probable expression of the human SNCA transgene in hematopoietic cells, known to express Thy1.[93] This could suppress their immune function, a phenomenon observed in other human SNCA transgenic mice.[94] We cannot exclude that a deeper investigation may uncover signs of subtle inflammation in our model. While inflammation certainly plays a role in initiating and driving pathologies in PD and in many models thereof, our result here indicates that, at least in some scenarios, it is not necessary to exacerbate PD-like pathologies.

Figure 6. Curli-driven nigrostriatal alpha-synuclein aggregation and neurodegeneration in Thy1-Syn14 mice was exacerbated by fiber deprivation

(A and B) Quantification and representative images of immunofluorescent pS129-αSyn stainings of the (A) dorsal striatum and (B) the SNpc. (A) The pS129-αSyn-positive area occupied was increased by the EC challenge. The FD diet challenge contributed to a lesser extent. The representative images (40×, scale bar: 50 μm) below illustrate the differences in pS129-αSyn accumulations between FR ΔEC- (top) and FR EC- (bottom) challenged TG mice. Sample sizes: FD PBS, n = 4; FR ΔEC, n = 5; FR EC, n = 7; FD ΔEC, n = 8; and FD EC, n = 8. (B) In the SNpc, we distinguished between accumulations in cell bodies (left panel) and other forms (right panel) based on cellular morphology. See STAR Methods for details on quantification. In EC-challenged animals, we found increased pS129-αSyn-positive aggregates. For other structures (see details in the main text), FD exacerbated the EC-induced pathology. The representative images (left: 10× tiles, scale bar: 250 μm; right: zoom in, scale bar: 100 μm) below illustrate the different observed structures of pS129-αSyn-positive structures (see details on the different forms in main text) in FD EC-challenged TG mice. Sample sizes: FD PBS, n = 4; FR ΔEC, n = 5; FR EC, n = 7; FD ΔEC, n = 8; and FD EC, n = 8.

(C–E) Quantification and representative images for (C) TH and (D) DAT in the dorsal striatum and (E) TH in the SNpc. (C and D) FD EC transgenic animals exhibited a decrease in axonal and synaptic density. The representative high-magnification (40x, scale bar: 50 μm) images illustrate the differences between FR ΔEC (top row) and FD EC (bottom row) TG mice. Sample sizes: FD PBS, n = 4; FR ΔEC, n = 5; FR EC, n = 8; FD ΔEC, n = 8; and FD EC, n = 8. (E) In the SNpc, curli drove neurodegeneration independently of the diet. FD did however exacerbate the pathology. The representative images (10×; scale bar: 250 μm) illustrate the average differences between FR ΔEC (top) and FD EC (bottom) transgenic animals. Sample sizes: FD PBS, n = 4; FR ΔEC, n = 5; FR EC, n = 7; FD ΔEC, n = 8; and FD EC, n = 8. All results were analyzed by Mann-Whitney U test, not corrected for FDR. See Table S2.

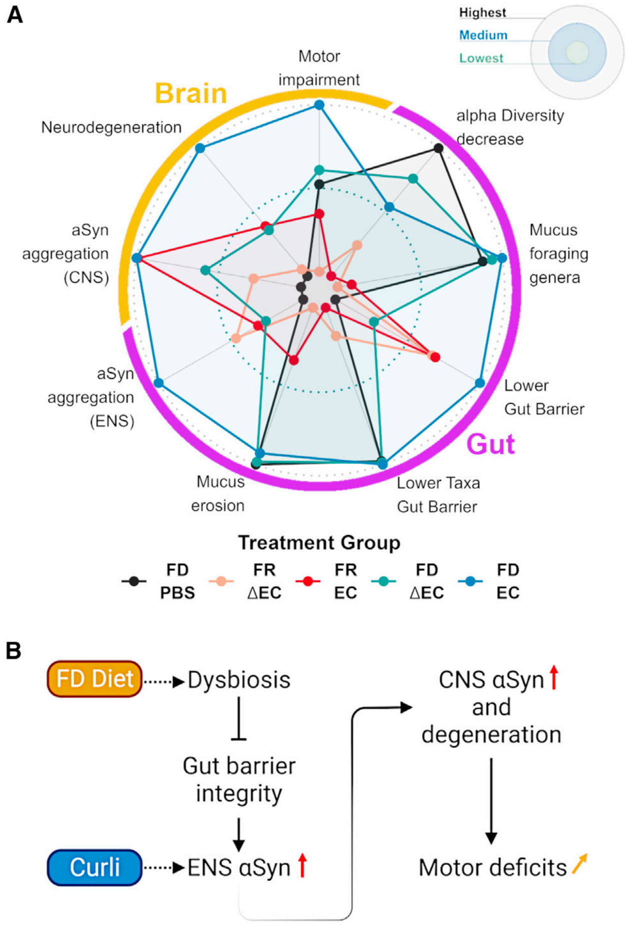

Figure 7. A multi-challenge-driven sequence of events for PD progression

(A) Radar plot of the total output for each challenge group in Thy1-Syn14 mice. The center of the plot defines the treatment with the lowest and the outline the one that showed the highest effect on Thy1-Syn14 mice. The combination of FD and EC had the greatest impact on αSyn-overexpressing mice. View main text for details.

(B) Putative sequence of events based on the results in this study. FD challenge leads to changes in the microbiome (dysbiosis), which affects gut barrier integrity and consequently facilitates the interaction of curli with neurons in the submucosa and plexuses. As a result, αSyn aggregates. This propagates to the brain, where αSyn aggregation increases, accompanied by neurodegeneration in the nigrostriatal pathway and exacerbation of motor deficits. See Table S2.

Multi-factor-driven exacerbation of PD pathologies in Thy1-Syn14 mice

To obtain an integrated view, parsing out the contribution of each challenge to PD pathology progression, we generated a radar plot. We simplified the output by classifying the results of the different treatment groups from lowest to highest (Figure 7A). We further split our findings over two categories (brain and gut) and eight sub-categories (gut: alpha diversity decrease, mucus foraging genera, lower gut barrier, lower taxa gut barrier, mucus erosion, and αSyn aggregation (ENS); brain: motor impairment, neurodegeneration, and αSyn aggregation (CNS); Figure 7A).

In TG mice, fiber deprivation was the underlying cause for changes in the gut microbiome and barrier, while bacterial curli drove enhanced αSyn aggregation in both ENS and CNS (Figure 7A). However, overall, the combination of the different challenges (TG FD EC) had the greatest effect on all PD-relevant pathologies (Figure 7A).

We propose a sequence of events (Figure 7B), whereby chronic dietary fiber deprivation leads to changes in microbial populations, gut mucus erosion, and increased gut permeability. The resulting higher exposure to amyloidogenic curli causes increased αSyn aggregation in the ENS and CNS, leading to neurodegeneration in the nigrostriatal pathway and exacerbation of motor deficits.

DISCUSSION

While the identification of genetic and environmental factors that modulate PD risk has progressed rapidly,[95,96] less is known about factors that modulate PD progression.[9] Our findings propose a sequence of events, starting with diet-induced dysbiosis, leading to reduced gut barrier integrity, which increases bacterial amyloidogenic protein entry, and ultimately culminating in exacerbation of PD pathologies in ENS and CNS.

The impact of diet on microbial gut health has been widely reported. Rodent studies reported rapid shifts in microbial gut populations after fiber deprivation.[15,16,97,98] Accordingly, our data showed decreased diversity and altered abundances of many PD-associated taxa when mice were fed an FD diet. The lack of dietary fiber makes specialized taxa, which are equipped with glycan-degrading enzymes, such as *Akkermansia* spp. or the *Bacteriodetes* genera *Alistipes* spp. and *Bacteroides* spp.,[65] switch to host glycans as energy resource, promoting mucus erosion and pathogens susceptibility.[16,65,99] Longer-term lack of dietary fiber triggers a compensatory mechanism, increasing mucin production, and re-establishing at least the inner mucus thickness.[15] In our study, only the outer mucus layer showed treatment-induced thinning. The thin outer mucus layer was associated with reduced bacterial diversity, possibly leading to changes in host-microbe interactions. We further observed reduced abundances of the butyrate-producing genera *Lachnospiraceaea NK4A136* and *Roseburia.* Additionally, *Lactobacillus*, which was also reduced in FD mice, was shown to stimulate butyrate production by such bacteria.[100] Recent studies showed the impact of microbial metabolite changes on gut barrier integrity. The metabolite butyrate, for instance, is essential in regulating energy metabolism, proliferation, and differentiation of gut epithelial cells, has anti-inflammatory properties, stimulates mucin production, and, importantly, promotes gut barrier protection by stimulating expression of ZO-1, ZO-2, cingulin, and occludin.[64,101] Our findings on gut barrier integrity revealed the lowest levels of tight junction proteins ZO-1 and occludin in TG mice challenged by fiber deprivation and curli. This supports the notion that these environmental challenges work in concert with abnormal αSyn to promote a leaky gut barrier. In intestinal biopsies from patients with PD, especially occludin was found at lower levels, whereas ZO-1 was unchanged.[102] Future studies will have to parse out the effect of different environmental challenges on features of gut barrier integrity.

Curli is a bacterial protein produced by *Enterobacteriaceae*. This bacterial family is increased in patients with PD and is associated with disease severity.[103,104] Its role as an αSyn cross-seeding agent, in the promotion of αSyn aggregation and worsening of motor symptoms, was documented in different models, such as *C. elegans*,[105] Fisher rats,[27] and another line of αSyn-overexpressing mice.[28] Enhanced curli exposure in PD could be the result of its secretion by *Enterobacteriaceae* for biofilm formation covering the intestinal mucosa. These biofilms could form as a consequence of excessive consumption of antibiotics.[106] Interestingly, excessive and repeated antibiotic consumption leads to enrichment of other PD-relevant taxa such as *A. muciniphila*,[107] and it is linked to PD risk.[108,109] Either gavaged[27] or supplemented in a human fecal microbiota transplant,[28] curli promotes PD pathologies, such as αSyn aggregation, in gut and brain.

PD initiation and progression are modulated by numerous external factors, for which an important entry point is the gut and from where abnormal αSyn can spread in a prion-like manner via the vagus nerve to the brain.[33,110] One of the most obvious factors is diet. A "Western" diet poor in fiber worsens PD progression, whereas a FR diet delays it.[13] In this study, using an αSyn transgenic mouse model, we show how these factors work together in worsening PD progression. Fiber deprivation shifts the composition of the microbiome toward bacteria that harm gut barrier integrity. Dysbiosis in PD promotes bacteria that produce amyloidogenic proteins.[111] We show that oral administration of *E. coli* to αSyn-overexpressing mice exacerbates PD-like pathologies, such as phosphorylated αSyn-positive structures and neurodegeneration, which are more pronounced in mice fed an FD diet. Thus, our study indicates that, in the context of initial PD-like disease induced by abnormal αSyn production, PD pathologies are significantly precipitated by environmental factors' action through the gut and its microbiome.

An unexpected observation of our study was the absence of substantial central and peripheral inflammation, which was probably due to αSyn transgene-mediated immunosuppression. Indeed, hematopoietic cells express Thy1.[93] Alpha-synuclein overexpression in isolated microglia and peripheral monocytes greatly reduces, in other αSyn overexpressing mice, the cytokine release by these cells after exposure to lipopolysaccharide.[94] We tentatively conclude that inflammation is not necessary to exacerbate the PD-like pathologies we observed.

In conclusion, we propose a sequence of interacting events involving exogenous and endogenous factors driving PD progression. We underline the importance of a balanced diet in limiting that progression. As their ability to perform activities of daily living declines over time,[112] patients with PD may require special assistance to ensure an optimal diet and oversight of antibiotics' use that could favor the proliferation of curli-producing intestinal bacteria. Our study puts forth the idea of lifestyle adaptations to mitigate PD progression.

Limitations of the study

While our study sheds light on interactions between diet, loss of gut barrier integrity, amyloidogenic protein of commensal bacteria, and the manifestations of PD pathologies, the underlying molecular mechanisms of these interactions remain to be uncovered. It is known that bacterial curli cross-seeds with αSyn and thus can initiate a pathological cascade. But how αSyn propagates and which misfolded form(s) of αSyn is/are toxic, in particular in our model after challenges, is unknown. There was no evidence of inflammation in the challenged mice of this study, and the downstream pathological effects of misfolded αSyn also need further investigation. The use of a strong pro-inflammatory challenge, such as LPS or αSyn preformed fibrils, in a study design similar to ours, followed by investigation of mitochondrial, lysosomal, synaptic dysfunctions, and of oxidative and inflammatory stress, may reveal a more complete picture of how PD progression is modulated. Finally, to strengthen the translational relevance of our findings, future studies should include other PD models, ideally in mice of various genetic strain backgrounds that are better models of genetic diversity in human patients.

STAR★METHODS

Detailed methods are provided in the online version of this paper and include the following:

- KEY RESOURCES TABLE
- RESOURCE AVAILABILITY
  - Lead contact
  - Materials availability
  - Data and code availability
- EXPERIMENTAL MODEL AND SUBJECT DETAILS
  - Ethical Approval
  - Mice
- METHOD DETAILS
  - Experimental design
  - Bacterial solution preparation and gavage
  - Tissue collection and preparation
  - RNA extraction and RT-qPCR for midbrain and striatal αSyn characterization
  - Protein extraction and western blot
  - Microbial DNA extraction and 16S rRNA gene amplicon sequencing
  - 16S rRNA gene amplicon sequence analysis
  - Microbial diversity and related statistics
  - *E. coli* quantitative Real-Time PCR (qRT-PCR)
  - Calprotectin ELISA
  - Plasma cytokine measurements
  - Alcian blue staining and outer mucus thickness measurements
  - Immunofluorescent staining of colon sections
  - Behavior
  - Immunofluorescent staining on free-floating brain sections
  - Quantitative neuropathology
  - Double IF and thioflavin-S staining on free-floating sagittal brain sections
- QUANTIFICATION AND STATISTICAL ANALYSIS

SUPPLEMENTAL INFORMATION

Supplemental information can be found online at https://doi.org/10.1016/j.celrep.2023.113071.

ACKNOWLEDGMENTS

This project received funding from the European Research Council (ERC) under the European Union's Horizon 2020 research and innovation programme (grant agreement no. 863664). The following people were supported by the Luxembourg National Research Fund (FNR): K.J.S. (fellowship AFR 12515776), A.S. (DTU PRIDE17/12244779/PARK-QC), and M.M. (PEARL P16/BM/11192868). The authors thank the Jean Think Foundation (Luxembourg) for support, Wagner Zago (Prothena) for the 11A5 antibody, Matt Chapman (University of Michigan) for the *E. coli* strains, Luc Buee (University of Lille) for hTau mice, Lennart Mucke (Gladstone Institutes) for J20-hAPP mouse tissues, and the Animal Facility staff at the University of Luxembourg for help.

AUTHORS CONTRIBUTIONS

K.J.S., M.B., E.C.M., and P.W. designed the study. K.J.S., P.G., A.S., B.P.R., M.H.T., J.-J.G., I.B.A., C.C., L.G., T.L., and T.H. did the experiments. R.H. performed the 16S rRNA amplicon sequencing. V.T.E.A. analyzed the 16S rRNA gene amplicon sequencing data. K.J.S., A.S., V.T.E.A., P.G., I.O., S.B.B., Y.S., M.M., M.B., and P.W. analyzed and interpreted the data. K.J.S. drafted the paper. M.B. edited the paper. All authors read and approved the final manuscript.

DECLARATION OF INTERESTS

The authors declare no competing interests.

INCLUSION AND DIVERSITY

We support inclusive, diverse, and equitable conduct of research.

Received: July 29, 2022
Revised: July 1, 2023
Accepted: August 16, 2023
Published: September 8, 2023

REFERENCES

1. Al-Maskari, F. LIFESTYLE DISEASES: An Economic Burden on the Health Services. United Nations. https://www.un.org/en/chronicle/article/lifestyle-diseases-economic-burden-health-services.
2. National Research Council (US); Institute of Medicine (US); Woolf, S.H., and Aron, L. (2013). Physical and Social Environmental Factors (National Academies Press (US)).
3. GBD 2016 Parkinson's Disease Collaborators; Elbaz, A., Nichols, E., Abbasi, N., Abd-Allah, F., Abdelalim, A., Adsuar, J.C., Ansha, M.G., Brayne, C., Choi, J.-Y.J., et al. (2018). Global, regional, and national burden of Parkinson's disease, 1990–2016: a systematic analysis for the Global Burden of Disease Study 2016. Lancet Neurol. *17*, 939–953. https://doi.org/10.1016/S1474-4422(18)30295-3.
4. Yang, W., Hamilton, J.L., Kopil, C., Beck, J.C., Tanner, C.M., Albin, R.L., Ray Dorsey, E., Dahodwala, N., Cintina, I., Hogan, P., and Thompson, T. (2020). Current and projected future economic burden of Parkinson's disease in the. NPJ Parkinsons Dis. *6*, 15–19. https://doi.org/10.1038/s41531-020-0117-1.
5. Poewe, W., Seppi, K., Tanner, C.M., Halliday, G.M., Brundin, P., Volkmann, J., Schrag, A.-E., and Lang, A.E. (2017). Parkinson disease. Nat. Rev. Dis. Prim. *3*, 17013–17021. https://doi.org/10.1038/nrdp.2017.13.
6. Shameem, A., Muliyar, S., Thankachan, R.P., Kalliath, J.T., Mangalath, U., and Mangalath, S. (2018). Study to evaluate the Efficacy of Resin-modified Glass Ionomer Cement Liner as a Direct Pulp Capping Material. J. Contemp. Dent. Pract. *19*, 1065–1071.
7. Gorell, J.M., Peterson, E.L., Rybicki, B.A., and Johnson, C.C. (2004). Multiple risk factors for Parkinson's disease. J. Neurol. Sci. *217*, 169–174. https://doi.org/10.1016/j.jns.2003.09.014.
8. Marras, C., Canning, C.G., and Goldman, S.M. (2019). Environment, lifestyle, and Parkinson's disease: Implications for prevention in the next decade. Mov. Disord. *34*, 801–811. https://doi.org/10.1002/mds.27720.
9. Nag, N., and Jelinek, G.A. (2019). More Research Is Needed on Lifestyle Behaviors That Influence Progression of Parkinson's Disease. Front. Neurol. *10*, 452.
10. Maraki, M.I., Yannakoulia, M., Stamelou, M., Stefanis, L., Xiromerisiou, G., Kosmidis, M.H., Dardiotis, E., Hadjigeorgiou, G.M., Sakka, P., Anastasiou, C.A., et al. (2019). Mediterranean diet adherence is related to reduced probability of prodromal Parkinson's disease. Mov. Disord. *34*, 48–57. https://doi.org/10.1002/mds.27489.
11. Hirschberg, S., Gisevius, B., Duscha, A., and Haghikia, A. (2019). Implications of Diet and The Gut Microbiome in Neuroinflammatory and Neurodegenerative Diseases. Int. J. Mol. Sci. *20*, 3109. https://doi.org/10.3390/ijms20123109.
12. Martínez Leo, E.E., and Segura Campos, M.R. (2020). Effect of ultra-processed diet on gut microbiota and thus its role in neurodegenerative diseases. Nutrition *71*, 110609. https://doi.org/10.1016/j.nut.2019.110609.
13. Mischley, L.K., Lau, R.C., and Bennett, R.D. (2017). Role of Diet and Nutritional Supplements in Parkinson's Disease Progression. Oxid. Med. Cell. Longev. *2017*, 6405278. https://doi.org/10.1155/2017/6405278.
14. Rampelli, S., Schnorr, S.L., Consolandi, C., Turroni, S., Severgnini, M., Peano, C., Brigidi, P., Crittenden, A.N., Henry, A.G., and Candela, M. (2015). Metagenome Sequencing of the Hadza Hunter-Gatherer Gut Microbiota. Curr. Biol. *25*, 1682–1693. https://doi.org/10.1016/j.cub.2015.04.055.
15. Schroeder, B.O., Birchenough, G.M.H., Ståhlman, M., Arike, L., Johansson, M.E.V., Hansson, G.C., and Bäckhed, F. (2018). Bifidobacteria or Fiber Protects against Diet-Induced Microbiota-Mediated Colonic Mucus Deterioration. Cell Host Microbe *23*, 27–40.e7. https://doi.org/10.1016/j.chom.2017.11.004.
16. Desai, M.S., Seekatz, A.M., Koropatkin, N.M., Kamada, N., Hickey, C.A., Wolter, M., Pudlo, N.A., Kitamoto, S., Terrapon, N., Muller, A., et al. (2016). A dietary fiber-deprived gut microbiota degrades the colonic mucus barrier and enhances pathogen susceptibility. Cell *167*, 1339–1353.e21. https://doi.org/10.1016/j.cell.2016.10.043.
17. Hasani, A., Ebrahimzadeh, S., Hemmati, F., Khabbaz, A., Hasani, A., and Gholizadeh, P. (2021). The role of Akkermansia muciniphila in obesity, diabetes and atherosclerosis. J. Med. Microbiol. *70*. https://doi.org/10.1099/jmm.0.001435.
18. Rosario, D., Bidkhori, G., Lee, S., Bedarf, J., Hildebrand, F., Le Chatelier, E., Uhlen, M., Ehrlich, S.D., Proctor, G., Wüllner, U., et al. (2021). Systematic analysis of gut microbiome reveals the role of bacterial folate and homocysteine metabolism in Parkinson's disease. Cell Rep. *34*, 108807. https://doi.org/10.1016/j.celrep.2021.108807.
19. Nerius, M., Doblhammer, G., and Tamgüney, G. (2020). GI infections are associated with an increased risk of Parkinson's disease. Gut *69*, 1154–1156. https://doi.org/10.1136/gutjnl-2019-318822.
20. Miller, A.L., Bessho, S., Grando, K., and Tükel, Ç. (2021). Microbiome or Infections: Amyloid-Containing Biofilms as a Trigger for Complex Human Diseases. Front. Immunol. *12*, 638867. https://doi.org/10.3389/fimmu.2021.638867.
21. Johansson, M.E.V., Larsson, J.M.H., and Hansson, G.C. (2011). The two mucus layers of colon are organized by the MUC2 mucin, whereas the outer layer is a legislator of host–microbial interactions. Proc. Natl. Acad. Sci. USA *108*, 4659–4665. https://doi.org/10.1073/pnas.1006451107.

22. Johansson, M.E.V., Sjövall, H., and Hansson, G.C. (2013). The gastrointestinal mucus system in health and disease. Nat. Rev. Gastroenterol. Hepatol. *10*, 352–361. https://doi.org/10.1038/nrgastro.2013.35.

23. de Vos, W.M. (2015). Microbial biofilms and the human intestinal microbiome. npj Biofilms Microbiomes *1*, 15005. https://doi.org/10.1038/npjbiofilms.2015.5.

24. Tytgat, H.L.P., Nobrega, F.L., van der Oost, J., and de Vos, W.M. (2019). Bowel Biofilms: Tipping Points between a Healthy and Compromised Gut? Trends Microbiol. *27*, 17–25. https://doi.org/10.1016/j.tim.2018.08.009.

25. Chapman, M.R., Robinson, L.S., Pinkner, J.S., Roth, R., Heuser, J., Hammar, M., Normark, S., and Hultgren, S.J. (2002). Role of Escherichia coli curli operons in directing amyloid fiber formation. Science *295*, 851–855. https://doi.org/10.1126/science.1067484.

26. Barnhart, M.M., and Chapman, M.R. (2006). Curli biogenesis and function. Annu. Rev. Microbiol. *60*, 131–147. https://doi.org/10.1146/annurev.micro.60.080805.142106.

27. Chen, S.G., Stribinskis, V., Rane, M.J., Demuth, D.R., Gozal, E., Roberts, A.M., Jagadapillai, R., Liu, R., Choe, K., Shivakumar, B., et al. (2016). Exposure to the Functional Bacterial Amyloid Protein Curli Enhances Alpha-Synuclein Aggregation in Aged Fischer 344 Rats and Caenorhabditis elegans. Sci. Rep. *6*, 34477. https://doi.org/10.1038/srep34477.

28. Sampson, T.R., Challis, C., Jain, N., Moiseyenko, A., Ladinsky, M.S., Shastri, G.G., Thron, T., Needham, B.D., Horvath, I., Debelius, J.W., et al. (2020). A gut bacterial amyloid promotes α-synuclein aggregation and motor impairment in mice. Elife *9*, e53111. https://doi.org/10.7554/eLife.53111.

29. Wakabayashi, K., Takahashi, H., Takeda, S., Ohama, E., and Ikuta, F. (1988). Parkinson's disease: the presence of Lewy bodies in Auerbach's and Meissner's plexuses. Acta Neuropathol. *76*, 217–221. https://doi.org/10.1007/BF00687767.

30. Wakabayashi, K., Takahashi, H., Ohama, E., Takeda, S., and Ikuta, F. (1993). Lewy bodies in the visceral autonomic nervous system in Parkinson's disease. Adv. Neurol. *60*, 609–612.

31. Wakabayashi, K., Takahashi, H., Ohama, E., and Ikuta, F. (1990). Parkinson's disease: an immunohistochemical study of Lewy body-containing neurons in the enteric nervous system. Acta Neuropathol. *79*, 581–583. https://doi.org/10.1007/BF00294234.

32. Qualman, S.J., Haupt, H.M., Yang, P., and Hamilton, S.R. (1984). Esophageal Lewy bodies associated with ganglion cell loss in achalasia. Similarity to Parkinson's disease. Gastroenterology *87*, 848–856.

33. Braak, H., de Vos, R.A.I., Bohl, J., and Del Tredici, K. (2006). Gastric alpha-synuclein immunoreactive inclusions in Meissner's and Auerbach's plexuses in cases staged for Parkinson's disease-related brain pathology. Neurosci. Lett. *396*, 67–72. https://doi.org/10.1016/j.neulet.2005.11.012.

34. Kahle, P.J., Neumann, M., Ozmen, L., Müller, V., Odoy, S., Okamoto, N., Jacobsen, H., Iwatsubo, T., Trojanowski, J.Q., Takahashi, H., et al. (2001). Selective insolubility of alpha-synuclein in human Lewy body diseases is recapitulated in a transgenic mouse model. Am. J. Pathol. *159*, 2215–2225. https://doi.org/10.1016/s0002-9440(10)63072-6.

35. Drokhlyansky, E., Smillie, C.S., Van Wittenberghe, N., Ericsson, M., Griffin, G.K., Eraslan, G., Dionne, D., Cuoco, M.S., Goder-Reiser, M.N., Sharova, T., et al. (2020). The Human and Mouse Enteric Nervous System at Single-Cell Resolution. Cell *182*, 1606–1622.e23. https://doi.org/10.1016/j.cell.2020.08.003.

36. Keller, D., Erö, C., and Markram, H. (2018). Cell Densities in the Mouse Brain: A Systematic Review. Front. Neuroanat. *12*, 83. https://doi.org/10.3389/fnana.2018.00083.

37. Murakami, T.C., Mano, T., Saikawa, S., Horiguchi, S.A., Shigeta, D., Baba, K., Sekiya, H., Shimizu, Y., Tanaka, K.F., Kiyonari, H., et al. (2018). A three-dimensional single-cell-resolution whole-brain atlas using CUBIC-X expansion microscopy and tissue clearing. Nat. Neurosci. *21*, 625–637. https://doi.org/10.1038/s41593-018-0109-1.

38. Zhang, J., Pho, V., Bonasera, S.J., Holtzman, J., Tang, A.T., Hellmuth, J., Tang, S., Janak, P.H., Tecott, L.H., and Huang, E.J. (2007). Essential function of HIPK2 in TGFβ-dependent survival of midbrain dopamine neurons. Nat. Neurosci. *10*, 77–86. https://doi.org/10.1038/nn1816.

39. Zhang, Y., Granholm, A.-C., Huh, K., Shan, L., Diaz-Ruiz, O., Malik, N., Olson, L., Hoffer, B.J., Lupica, C.R., Hoffman, A.F., and Bäckman, C.M. (2012). PTEN deletion enhances survival, neurite outgrowth and function of dopamine neuron grafts to MitoPark mice. Brain *135*, 2736–2749. https://doi.org/10.1093/brain/aws196.

40. Barber Janer, A., Vonck, E., and Baekelandt, V. (2021). Chapter Two - Modeling synucleinopathies in rodents. In International Review of Movement Disorders Mechanisms of Cell Death and Approaches to Neuroprotection/Disease Modification in Parkinson's Disease, B. Dehay and E. Bezard, eds. (Academic Press), pp. 65–154. https://doi.org/10.1016/bs.irmvd.2021.09.001.

41. Chen, H., and Ritz, B. (2018). The Search for Environmental Causes of Parkinson's Disease: Moving Forward. J. Parkinsons Dis. *8*, S9–S17. https://doi.org/10.3233/JPD-181493.

42. Di Monte, D.A., Lavasani, M., and Manning-Bog, A.B. (2002). Environmental Factors in Parkinson's Disease. Neurotoxicology *23*, 487–502. https://doi.org/10.1016/S0161-813X(02)00099-2.

43. Dick, F.D., De Palma, G., Ahmadi, A., Scott, N.W., Prescott, G.J., Bennett, J., Semple, S., Dick, S., Counsell, C., Mozzoni, P., et al. (2007). Environmental risk factors for Parkinson's disease and parkinsonism: the Geoparkinson study. Occup. Environ. Med. *64*, 666–672. https://doi.org/10.1136/oem.2006.027003.

44. Warner, T.T., and Schapira, A.H.V. (2003). Genetic and environmental factors in the cause of Parkinson's disease. Ann. Neurol. *53*, S16–S23. https://doi.org/10.1002/ana.10487.

45. Bernardo-Cravo, A.P., Schmeller, D.S., Chatzinotas, A., Vredenburg, V.T., and Loyau, A. (2020). Environmental Factors and Host Microbiomes Shape Host–Pathogen Dynamics. Trends Parasitol. *36*, 616–633. https://doi.org/10.1016/j.pt.2020.04.010.

46. Singh, Y., El-Hadidi, M., Admard, J., Wassouf, Z., Schulze-Hentrich, J.M., Kohlhofer, U., Quintanilla-Martinez, L., Huson, D., Riess, O., and Casadei, N. (2019). Enriched Environmental Conditions Modify the Gut Microbiome Composition and Fecal Markers of Inflammation in Parkinson's Disease. Front. Neurosci. *13*, 1032. https://doi.org/10.3389/fnins.2019.01032.

47. Boertien, J.M., Pereira, P.A.B., Aho, V.T.E., and Scheperjans, F. (2019). Increasing Comparability and Utility of Gut Microbiome Studies in Parkinson's Disease: A Systematic Review. J. Parkinsons Dis. *9*, S297–S312. https://doi.org/10.3233/JPD-191711.

48. Gerhardt, S., and Mohajeri, M.H. (2018). Changes of Colonic Bacterial Composition in Parkinson's Disease and Other Neurodegenerative Diseases. Nutrients *10*, 708. https://doi.org/10.3390/nu10060708.

49. Heintz-Buschart, A., Pandey, U., Wicke, T., Sixel-Döring, F., Janzen, A., Sittig-Wiegand, E., Trenkwalder, C., Oertel, W.H., Mollenhauer, B., and Wilmes, P. (2018). The nasal and gut microbiome in Parkinson's disease and idiopathic rapid eye movement sleep behavior disorder. Mov. Disord. *33*, 88–98. https://doi.org/10.1002/mds.27105.

50. Keshavarzian, A., Green, S.J., Engen, P.A., Voigt, R.M., Naqib, A., Forsyth, C.B., Mutlu, E., and Shannon, K.M. (2015). Colonic bacterial composition in Parkinson's disease. Mov. Disord. *30*, 1351–1360. https://doi.org/10.1002/mds.26307.

51. Scheperjans, F., Aho, V., Pereira, P.A.B., Koskinen, K., Paulin, L., Pekkonen, E., Haapaniemi, E., Kaakkola, S., Eerola-Rautio, J., Pohja, M., et al. (2015). Gut microbiota are related to Parkinson's disease and clinical phenotype. Mov. Disord. *30*, 350–358. https://doi.org/10.1002/mds.26069.

52. Shen, T., Yue, Y., He, T., Huang, C., Qu, B., Lv, W., and Lai, H.-Y. (2021). The Association Between the Gut Microbiota and Parkinson's Disease, a Meta-Analysis. Front. Aging Neurosci. *13*, 636545. https://doi.org/10.3389/fnagi.2021.636545.

53. Unger, M.M., Spiegel, J., Dillmann, K.-U., Grundmann, D., Philippeit, H., Bürmann, J., Faßbender, K., Schwiertz, A., and Schäfer, K.H. (2016). Short chain fatty acids and gut microbiota differ between patients with Parkinson's disease and age-matched controls. Parkinsonism Relat. Disorders *32*, 66–72. https://doi.org/10.1016/j.parkreldis.2016.08.019.

54. Gorecki, A.M., Preskey, L., Bakeberg, M.C., Kenna, J.E., Gildenhuys, C., MacDougall, G., Dunlop, S.A., Mastaglia, F.L., Akkari, P.A., Koengten, F., and Anderton, R.S. (2019). Altered Gut Microbiome in Parkinson's Disease and the Influence of Lipopolysaccharide in a Human α-Synuclein Over-Expressing Mouse Model. Front. Neurosci. *13*, 839. https://doi.org/10.3389/fnins.2019.00839.

55. Sampson, T.R., Debelius, J.W., Thron, T., Janssen, S., Shastri, G.G., Ilhan, Z.E., Challis, C., Schretter, C.E., Rocha, S., Gradinaru, V., et al. (2016). Gut Microbiota Regulate Motor Deficits and Neuroinflammation in a Model of Parkinson's Disease. Cell *167*, 1469–1480.e12. https://doi.org/10.1016/j.cell.2016.11.018.

56. Yan, Y., Ren, S., Duan, Y., Lu, C., Niu, Y., Wang, Z., Inglis, B., Ji, W., Zheng, Y., and Si, W. (2021). Gut microbiota and metabolites of α-synuclein transgenic monkey models with early stage of Parkinson's disease. npj Biofilms Microbiomes *7*, 69–9. https://doi.org/10.1038/s41522-021-00242-3.

57. Magne, F., Gotteland, M., Gauthier, L., Zazueta, A., Pesoa, S., Navarrete, P., and Balamurugan, R. (2020). The Firmicutes/Bacteroidetes Ratio: A Relevant Marker of Gut Dysbiosis in Obese Patients? Nutrients *12*, 1474. https://doi.org/10.3390/nu12051474.

58. Mariat, D., Firmesse, O., Levenez, F., Guimarăes, V., Sokol, H., Doré, J., Corthier, G., and Furet, J.-P. (2009). The Firmicutes/Bacteroidetes ratio of the human microbiota changes with age. BMC Microbiol. *9*, 123. https://doi.org/10.1186/1471-2180-9-123.

59. Heeney, D.D., Gareau, M.G., and Marco, M.L. (2018). Intestinal Lactobacillus in health and disease, a driver or just along for the ride? Curr. Opin. Biotechnol. *49*, 140–147. https://doi.org/10.1016/j.copbio.2017.08.004.

60. Martín, R., Miquel, S., Ulmer, J., Kechaou, N., Langella, P., and Bermúdez-Humarán, L.G. (2013). Role of commensal and probiotic bacteria in human health: a focus on inflammatory bowel disease. Microb. Cell Factories *12*, 71. https://doi.org/10.1186/1475-2859-12-71.

61. Wang, L., Li, S., Jiang, Y., Zhao, Z., Shen, Y., Zhang, J., and Zhao, L. (2021). Neuroprotective effect of Lactobacillus plantarum DP189 on MPTP-induced Parkinson's disease model mice. J. Funct.Foods *85*, 104635. https://doi.org/10.1016/j.jff.2021.104635.

62. Blackwood, B.P., Yuan, C.Y., Wood, D.R., Nicolas, J.D., Grothaus, J.S., and Hunter, C.J. (2017). Probiotic Lactobacillus Species Strengthen Intestinal Barrier Function and Tight Junction Integrity in Experimental Necrotizing Enterocolitis. J. Probiotics Health *5*, 159. https://doi.org/10.4172/2329-8901.1000159.

63. Stadlbauer, V., Engertsberger, L., Komarova, I., Feldbacher, N., Leber, B., Pichler, G., Fink, N., Scarpatetti, M., Schippinger, W., Schmidt, R., and Horvath, A. (2020). Dysbiosis, gut barrier dysfunction and inflammation in dementia: a pilot study. BMC Geriatr. *20*, 248. https://doi.org/10.1186/s12877-020-01644-2.

64. Plöger, S., Stumpff, F., Penner, G.B., Schulzke, J.-D., Gäbel, G., Martens, H., Shen, Z., Günzel, D., and Aschenbach, J.R. (2012). Microbial butyrate and its role for barrier function in the gastrointestinal tract. Ann. N. Y. Acad. Sci. *1258*, 52–59. https://doi.org/10.1111/j.1749-6632.2012.06553.x.

65. Glover, J.S., Ticer, T.D., and Engevik, M.A. (2022). Characterizing the mucin-degrading capacity of the human gut microbiota. Sci. Rep. *12*, 8456. https://doi.org/10.1038/s41598-022-11819-z.

66. Tailford, L.E., Crost, E.H., Kavanaugh, D., and Juge, N. (2015). Mucin glycan foraging in the human gut microbiome. Front. Genet. *6*, 81. https://doi.org/10.3389/fgene.2015.00081.

67. Chelakkot, C., Ghim, J., and Ryu, S.H. (2018). Mechanisms regulating intestinal barrier integrity and its pathological implications. Exp. Mol. Med. *50*, 1–9. https://doi.org/10.1038/s12276-018-0126-x.

68. Lee, B., Moon, K.M., and Kim, C.Y. (2018). Tight Junction in the Intestinal Epithelium: Its Association with Diseases and Regulation by Phytochemicals. J. Immunol. Res. *2018*, 2645465. https://doi.org/10.1155/2018/2645465.

69. Luissint, A.-C., Artus, C., Glacial, F., Ganeshamoorthy, K., and Couraud, P.-O. (2012). Tight junctions at the blood brain barrier: physiological architecture and disease-associated dysregulation. Fluids Barriers CNS *9*, 23. https://doi.org/10.1186/2045-8118-9-23.

70. Fasano, A. (2020). All disease begins in the (leaky) gut: role of zonulin-mediated gut permeability in the pathogenesis of some chronic inflammatory diseases. F1000Res. *9*, F1000 Faculty Rev-69. https://doi.org/10.12688/f1000research.20510.1.

71. Harsanyiova, J., Buday, T., and Kralova Trancikova, A. (2020). Parkinson's Disease and the Gut: Future Perspectives for Early Diagnosis. Front. Neurosci. *14*, 626. https://doi.org/10.3389/fnins.2020.00626.

72. Cummins, P.M. (2012). Occludin: One Protein, Many Forms. Mol. Cell Biol. *32*, 242–250. https://doi.org/10.1128/MCB.06029-11.

73. Mankertz, J., Waller, J.S., Hillenbrand, B., Tavalali, S., Florian, P., Schöneberg, T., Fromm, M., and Schulzke, J.D. (2002). Gene expression of the tight junction protein occludin includes differential splicing and alternative promoter usage. Biochem. Biophys. Res. Commun. *298*, 657–666. https://doi.org/10.1016/S0006-291X(02)02487-7.

74. McCaffrey, G., Willis, C.L., Staatz, W.D., Nametz, N., Quigley, C.A., Hom, S., Lochhead, J.J., and Davis, T.P. (2009). Occludin oligomeric assemblies at tight junctions of the blood-brain barrier are altered by hypoxia and reoxygenation stress. J. Neurochem. *110*, 58–71. https://doi.org/10.1111/j.1471-4159.2009.06113.x.

75. Del Tredici, K., and Braak, H. (2012). Lewy pathology and neurodegeneration in premotor Parkinson's disease. Mov. Disord. *27*, 597–607. https://doi.org/10.1002/mds.24921.

76. Del Tredici, K., and Duda, J.E. (2011). Peripheral Lewy body pathology in Parkinson's disease and incidental Lewy body disease: Four cases. J. Neurol. Sci. *310*, 100–106. https://doi.org/10.1016/j.jns.2011.06.003.

77. Vaikath, N.N., Hmila, I., Gupta, V., Erskine, D., Ingelsson, M., and El-Agnaf, O.M.A. (2019). Antibodies against alpha-synuclein: tools and therapies. J. Neurochem. *150*, 612–625. https://doi.org/10.1111/jnc.14713.

78. Shannon, K.M., Keshavarzian, A., Dodiya, H.B., Jakate, S., and Kordower, J.H. (2012). Is alpha-synuclein in the colon a biomarker for premotor Parkinson's disease? Evidence from 3 cases. Mov. Disord. *27*, 716–719. https://doi.org/10.1002/mds.25020.

79. Stokholm, M.G., Danielsen, E.H., Hamilton-Dutoit, S.J., and Borghammer, P. (2016). Pathological α-synuclein in gastrointestinal tissues from prodromal Parkinson disease patients. Ann. Neurol. *79*, 940–949. https://doi.org/10.1002/ana.24648.

80. Sidebotham, E.L., Woodward, M.N., Kenny, S.E., Lloyd, D.A., Vaillant, C.R., and Edgar, D.H. (2001). Assessment of protein gene product 9.5 as a marker of neural crest-derived precursor cells in the developing enteric nervous system. Pediatr. Surg. Int. *17*, 304–307. https://doi.org/10.1007/s003830100599.

81. Wang, Y., Pan, Y., Price, A., and Martin, L.J. (2011). Generation and characterization of transgenic mice expressing mitochondrial targeted red fluorescent protein selectively in neurons: modeling mitochondriopathy in excitotoxicity and amyotrophic lateral sclerosis. Mol. Neurodegener. *6*, 75. https://doi.org/10.1186/1750-1326-6-75.

82. Koss, D.J., Erskine, D., Porter, A., Palmoski, P., Menon, H., Todd, O.G.J., Leite, M., Attems, J., and Outeiro, T.F. (2022). Nuclear alpha-synuclein is present in the human brain and is modified in dementia with Lewy bodies.

Acta Neuropathol. Commun. *10*, 98. https://doi.org/10.1186/s40478-022-01403-x.

83. Lauwers, E., Debyser, Z., Van Dorpe, J., De Strooper, B., Nuttin, B., and Baekelandt, V. (2003). Neuropathology and Neurodegeneration in Rodent Brain Induced by Lentiviral Vectormediated Overexpression of α-Synuclein. Brain Pathol. *13*, 364–372. https://doi.org/10.1111/j.1750-3639.2003.tb00035.x.

84. Kouroupi, G., Taoufik, E., Vlachos, I.S., Tsioras, K., Antoniou, N., Papastefanaki, F., Chroni-Tzartou, D., Wrasidlo, W., Bohl, D., Stellas, D., et al. (2017). Defective synaptic connectivity and axonal neuropathology in a human iPSC-based model of familial Parkinson's disease. Proc. Natl. Acad. Sci. USA *114*, E3679–E3688. https://doi.org/10.1073/pnas.1617259114.

85. Del Tredici, K., Rüb, U., de Vos, R.A.I., Bohl, J.R.E., and Braak, H. (2002). Where Does Parkinson Disease Pathology Begin in the Brain? J. Neuropathol. Exp. Neurol. *61*, 413–426. https://doi.org/10.1093/jnen/61.5.413.

86. Garcia, P., Jürgens-Wemheuer, W., Uriarte Huarte, O., Michelucci, A., Masuch, A., Brioschi, S., Weihofen, A., Koncina, E., Coowar, D., Heurtaux, T., et al. (2022). Neurodegeneration and neuroinflammation are linked, but independent of alpha-synuclein inclusions, in a seeding/spreading mouse model of Parkinson's disease. Glia *70*, 935–960. https://doi.org/10.1002/glia.24149.

87. Soto, C., and Pritzkow, S. (2018). Protein misfolding, aggregation, and conformational strains in neurodegenerative diseases. Nat. Neurosci. *21*, 1332–1340. https://doi.org/10.1038/s41593-018-0235-9.

88. Tansey, M.G., Wallings, R.L., Houser, M.C., Herrick, M.K., Keating, C.E., and Joers, V. (2022). Inflammation and immune dysfunction in Parkinson disease. Nat. Rev. Immunol. *22*, 657–673. https://doi.org/10.1038/s41577-022-00684-6.

89. Lehmann, F.S., Burri, E., and Beglinger, C. (2015). The role and utility of faecal markers in inflammatory bowel disease. Therap. Adv. Gastroenterol. *8*, 23–36. https://doi.org/10.1177/1756283X14553384.

90. Walsham, N.E., and Sherwood, R.A. (2016). Fecal calprotectin in inflammatory bowel disease. Clin. Exp. Gastroenterol. *9*, 21–29. https://doi.org/10.2147/CEG.S51902.

91. Schwiertz, A., Spiegel, J., Dillmann, U., Grundmann, D., Bürmann, J., Faßbender, K., Schäfer, K.H., and Unger, M.M. (2018). Fecal markers of intestinal inflammation and intestinal permeability are elevated in Parkinson's disease. Parkinsonism Relat. Disorders *50*, 104–107. https://doi.org/10.1016/j.parkreldis.2018.02.022.

92. Amara, J., Saliba, Y., Hajal, J., Smayra, V., Bakhos, J.-J., Sayegh, R., and Fares, N. (2019). Circadian Rhythm Disruption Aggravates DSS-Induced Colitis in Mice with Fecal Calprotectin as a Marker of Colitis Severity. Dig. Dis. Sci. *64*, 3122–3133. https://doi.org/10.1007/s10620-019-05675-7.

93. Bradley, J.E., Ramirez, G., and Hagood, J.S. (2009). Roles and regulation of Thy-1, a context-dependent modulator of cell phenotype. Biofactors *35*, 258–265. https://doi.org/10.1002/biof.41.

94. Gardai, S.J., Mao, W., Schüle, B., Babcock, M., Schoebel, S., Lorenzana, C., Alexander, J., Kim, S., Glick, H., Hilton, K., et al. (2013). Elevated alpha-synuclein impairs innate immune cell function and provides a potential peripheral biomarker for Parkinson's disease. PLoS One *8*, e71634. https://doi.org/10.1371/journal.pone.0071634.

95. Bellou, V., Belbasis, L., Tzoulaki, I., Evangelou, E., and Ioannidis, J.P.A. (2016). Environmental risk factors and Parkinson's disease: An umbrella review of meta-analyses. Parkinsonism Relat. Disorders *23*, 1–9. https://doi.org/10.1016/j.parkreldis.2015.12.008.

96. Nalls, M.A., Blauwendraat, C., Vallerga, C.L., Heilbron, K., Bandres-Ciga, S., Chang, D., Tan, M., Kia, D.A., Noyce, A.J., Xue, A., et al. (2019). Identification of novel risk loci, causal insights, and heritable risk for Parkinson's disease: a meta-analysis of genome-wide association studies. Lancet Neurol. *18*, 1091–1102. https://doi.org/10.1016/S1474-4422(19)30320-5.

97. Neumann, M., Steimle, A., Grant, E.T., Wolter, M., Parrish, A., Willieme, S., Brenner, D., Martens, E.C., and Desai, M.S. (2021). Deprivation of dietary fiber in specific-pathogen-free mice promotes susceptibility to the intestinal mucosal pathogen Citrobacter rodentium. Gut Microb. *13*, 1966263. https://doi.org/10.1080/19490976.2021.1966263.

98. Riva, A., Kuzyk, O., Forsberg, E., Siuzdak, G., Pfann, C., Herbold, C., Daims, H., Loy, A., Warth, B., and Berry, D. (2019). A fiber-deprived diet disturbs the fine-scale spatial architecture of the murine colon microbiome. Nat. Commun. *10*, 4366. https://doi.org/10.1038/s41467-019-12413-0.

99. Martens, E.C., Chiang, H.C., and Gordon, J.I. (2008). Mucosal Glycan Foraging Enhances Fitness and Transmission of a Saccharolytic Human Gut Bacterial Symbiont. Cell Host Microbe *4*, 447–457. https://doi.org/10.1016/j.chom.2008.09.007.

100. Lin, R., Sun, Y., Mu, P., Zheng, T., Mu, H., Deng, F., Deng, Y., and Wen, J. (2020). Lactobacillus rhamnosus GG supplementation modulates the gut microbiota to promote butyrate production, protecting against deoxynivalenol exposure in nude mice. Biochem. Pharmacol. *175*, 113868. https://doi.org/10.1016/j.bcp.2020.113868.

101. Rivière, A., Selak, M., Lantin, D., Leroy, F., and De Vuyst, L. (2016). Bifidobacteria and Butyrate-Producing Colon Bacteria: Importance and Strategies for Their Stimulation in the Human Gut. Front. Microbiol. *7*, 979. https://doi.org/10.3389/fmicb.2016.00979.

102. Clairembault, T., Leclair-Visonneau, L., Coron, E., Bourreille, A., Le Dily, S., Vavasseur, F., Heymann, M.-F., Neunlist, M., and Derkinderen, P. (2015). Structural alterations of the intestinal epithelial barrier in Parkinson's disease. Acta Neuropathol. Commun. *3*, 12. https://doi.org/10.1186/s40478-015-0196-0.

103. Barichella, M., Severgnini, M., Cilia, R., Cassani, E., Bolliri, C., Caronni, S., Ferri, V., Cancello, R., Ceccarani, C., Faierman, S., et al. (2019). Unraveling gut microbiota in Parkinson's disease and atypical parkinsonism. Mov. Disord. *34*, 396–405. https://doi.org/10.1002/mds.27581.

104. Li, W., Wu, X., Hu, X., Wang, T., Liang, S., Duan, Y., Jin, F., and Qin, B. (2017). Structural changes of gut microbiota in Parkinson's disease and its correlation with clinical features. Sci. China Life Sci. *60*, 1223–1233. https://doi.org/10.1007/s11427-016-9001-4.

105. Wang, C., Lau, C.Y., Ma, F., and Zheng, C. (2021). Genome-wide screen identifies curli amyloid fibril as a bacterial component promoting host neurodegeneration. Proc. Natl. Acad. Sci. USA *118*, e2106504118. https://doi.org/10.1073/pnas.2106504118.

106. Hoffman, L.R., D'Argenio, D.A., MacCoss, M.J., Zhang, Z., Jones, R.A., and Miller, S.I. (2005). Aminoglycoside antibiotics induce bacterial biofilm formation. Nature *436*, 1171–1175. https://doi.org/10.1038/nature03912.

107. de Nies, L., Busi, S.B., Tsenkova, M., Halder, R., Letellier, E., and Wilmes, P. (2022). Evolution of the murine gut resistome following broad-spectrum antibiotic treatment. Nat. Commun. *13*, 2296. https://doi.org/10.1038/s41467-022-29919-9.

108. Mertsalmi, T.H., Pekkonen, E., and Scheperjans, F. (2020). Antibiotic exposure and risk of Parkinson's disease in Finland: A nationwide case-control study. Mov. Disord. *35*, 431–442. https://doi.org/10.1002/mds.27924.

109. Ternák, G., Kuti, D., and Kovács, K.J. (2020). Dysbiosis in Parkinson's disease might be triggered by certain antibiotics. Med. Hypotheses *137*, 109564. https://doi.org/10.1016/j.mehy.2020.109564.

110. Braak, H., Del Tredici, K., Rüb, U., de Vos, R.A.I., Jansen Steur, E.N.H., and Braak, E. (2003). Staging of brain pathology related to sporadic Parkinson's disease. Neurobiol. Aging *24*, 197–211. https://doi.org/10.1016/S0197-4580(02)00065-9.

111. Albani, G., Albani, S., and Keshavarzian, A. (2020). Editorial: Role of Diet, Physical Activity and Immune System in Parkinson's Disease. Front. Neurol. *11*, 611349. https://doi.org/10.3389/fneur.2020.611349.

112. Sperens, M., Georgiev, D., Eriksson Domellöf, M., Forsgren, L., Hamberg, K., and Hariz, G.-M. (2020). Activities of daily living in Parkinson's disease: Time/gender perspective. Acta Neurol. Scand. *141*, 168–176. https://doi.org/10.1111/ane.13189.

113. Smati, M., Clermont, O., Le Gal, F., Schichmanoff, O., Jauréguy, F., Eddi, A., Denamur, E., and Picard, B.; Coliville Group (2013). Real-Time PCR for Quantitative Analysis of Human Commensal Escherichia coli Populations Reveals a High Frequency of Subdominant Phylogroups. Appl. Environ. Microbiol. *79*, 5005–5012. https://doi.org/10.1128/AEM.01423-13.

114. Bacchetti De Gregoris, T., Aldred, N., Clare, A.S., and Burgess, J.G. (2011). Improvement of phylum- and class-specific primers for real-time PCR quantification of bacterial taxa. J. Microbiol. Methods *86*, 351–356. https://doi.org/10.1016/j.mimet.2011.06.010.

115. Schneider, C.A., Rasband, W.S., and Eliceiri, K.W. (2012). NIH Image to ImageJ: 25 years of image analysis. Nat. Methods *9*, 671–675. https://doi.org/10.1038/nmeth.2089.

116. Kahle, P.J., Neumann, M., Ozmen, L., Müller, V., Jacobsen, H., Schindzielorz, A., Okochi, M., Leimer, U., van Der Putten, H., Probst, A., et al. (2000). Subcellular Localization of Wild-Type and Parkinson's Disease-Associated Mutant α-Synuclein in Human and Transgenic Mouse Brain. J. Neurosci. *20*, 6365–6373. https://doi.org/10.1523/JNEUROSCI.20-17-06365.2000.

117. Yadav, N., Thakur, A.K., Shekhar, N., and Ayushi. (2021). Potential of Antibiotics for the Treatment and Management of Parkinson's Disease: An Overview. Curr. Drug Res. Rev. *13*, 166–171.

118. Garcia, P., Youssef, I., Utvik, J.K., Florent-Béchard, S., Barthélémy, V., Malaplate-Armand, C., Kriem, B., Stenger, C., Koziel, V., Olivier, J.-L., et al. (2010). Ciliary Neurotrophic Factor Cell-Based Delivery Prevents Synaptic Impairment and Improves Memory in Mouse Models of Alzheimer's Disease. J. Neurosci. *30*, 7516–7527. https://doi.org/10.1523/JNEUROSCI.4182-09.2010.

119. Keller, J.N., Mark, R.J., Bruce, A.J., Blanc, E., Rothstein, J.D., Uchida, K., Waeg, G., and Mattson, M.P. (1997). 4-Hydroxynonenal, an aldehydic product of membrane lipid peroxidation, impairs glutamate transport and mitochondrial function in synaptosomes. Neuroscience *80*, 685–696. https://doi.org/10.1016/S0306-4522(97)00065-1.

120. Dore, J., Ehrlich, S.D., Levenez, F., Pellecchia, M.T., Alberti, A., Bertrand, L., Bork, P., Costea, P.I., Sunagawa, S., Guarner, F., et al. (2015). IHMS_SOP 07 V1: Standard Operating Procedure for Fecal Samples DNA Extraction (Protocol H. International Human Microbiome Standards). http://www.microbiome-standards.org.

121. Callahan, B.J., McMurdie, P.J., Rosen, M.J., Han, A.W., Johnson, A.J.A., and Holmes, S.P. (2016). DADA2: High-resolution sample inference from Illumina amplicon data. Nat. Methods *13*, 581–583. https://doi.org/10.1038/nmeth.3869.

122. Quast, C., Pruesse, E., Yilmaz, P., Gerken, J., Schweer, T., Yarza, P., Peplies, J., and Glöckner, F.O. (2013). The SILVA ribosomal RNA gene database project: improved data processing and web-based tools. Nucleic Acids Res. *41*, D590–D596. https://doi.org/10.1093/nar/gks1219.

123. McMurdie, P.J., and Holmes, S. (2013). phyloseq: An R Package for Reproducible Interactive Analysis and Graphics of Microbiome Census Data. PLoS One *8*, e61217. https://doi.org/10.1371/journal.pone.0061217.

124. Oksanen, J., Blanchet, F.G., Friendly, M., Kindt, R., Legendre, P., McGlinn, D., Minchin, P.R., O'Hara, R.B., Simpson, G.L., Solymos, P., et al. (2020). Vegan: Community Ecology Package. https://cran.r-project.org/web/packages/vegan/index.html.

125. Guyenet, S.J., Furrer, S.A., Damian, V.M., Baughan, T.D., La Spada, A.R., and Garden, G.A. (2010). A Simple Composite Phenotype Scoring System for Evaluating Mouse Models of Cerebellar Ataxia. J. Vis. Exp. https://doi.org/10.3791/1787.

126. Mao, X., Ou, M.T., Karuppagounder, S.S., Kam, T.-I., Yin, X., Xiong, Y., Ge, P., Umanah, G.E., Brahmachari, S., Shin, J.-H., et al. (2016). Pathological α-synuclein transmission initiated by binding lymphocyte-activation gene 3. Science *353*, aah3374. https://doi.org/10.1126/science.aah3374.

127. Tillerson, J.L., Caudle, W.M., Reverón, M.E., and Miller, G.W. (2002). Detection of Behavioral Impairments Correlated to Neurochemical Deficits in Mice Treated with Moderate Doses of 1-Methyl-4-phenyl-1,2,3,6-tetrahydropyridine. Exp. Neurol. *178*, 80–90. https://doi.org/10.1006/exnr.2002.8021.

128. Tillerson, J.L., and Miller, G.W. (2003). Grid performance test to measure behavioral impairment in the MPTP-treated-mouse model of parkinsonism. J. Neurosci. Methods *123*, 189–200. https://doi.org/10.1016/S0165-0270(02)00360-6.

129. Bouet, V., Boulouard, M., Toutain, J., Divoux, D., Bernaudin, M., Schumann-Bard, P., and Freret, T. (2009). The adhesive removal test: a sensitive method to assess sensorimotor deficits in mice. Nat. Protoc. *4*, 1560–1564. https://doi.org/10.1038/nprot.2009.125.

130. Ashrafi, A., Garcia, P., Kollmus, H., Schughart, K., Del Sol, A., Buttini, M., and Glaab, E. (2017). Absence of regulator of G-protein signaling 4 does not protect against dopamine neuron dysfunction and injury in the mouse 6-hydroxydopamine lesion model of Parkinson's disease. Neurobiol. Aging *58*, 30–33. https://doi.org/10.1016/j.neurobiolaging.2017.06.008.

131. Bussière, T., Bard, F., Barbour, R., Grajeda, H., Guido, T., Khan, K., Schenk, D., Games, D., Seubert, P., and Buttini, M. (2004). Morphological characterization of Thioflavin-S-positive amyloid plaques in transgenic Alzheimer mice and effect of passive Abeta immunotherapy on their clearance. Am. J. Pathol. *165*, 987–995. https://doi.org/10.1016/s0002-9440(10)63360-3.

132. Schindowski, K., Bretteville, A., Leroy, K., Bégard, S., Brion, J.-P., Hamdane, M., and Buée, L. (2006). Alzheimer's disease-like tau neuropathology leads to memory deficits and loss of functional synapses in a novel mutated tau transgenic mouse without any motor deficits. Am. J. Pathol. *169*, 599–616. https://doi.org/10.2353/ajpath.2006.060002.

STAR★METHODS

KEY RESOURCES TABLE

REAGENT or RESOURCE	SOURCE	IDENTIFIER
Antibodies		
Chicken Polyclonal anti-Protein Gene Product 9.5	Abcam	Cat# ab72910; RRID:AB_1269734
Chicken Polyclonal anti-tyrosine hydroxylase	Abcam	Cat# ab76442; RRID:
Mouse Monoclonal anti-Occludin	Thermo Fisher SCientific	Cat# 33–1500; RRID:AB_87033
Mouse Monoclonal anti-α-Synuclein, clone Syn211	Sigma-Aldrich	Cat# S5566; RRID:AB_261518
Mouse Monoclonal anti-α-Tubulin antibody [DM1A] - Loading Control	Abcam	Cat# ab7291; RRID:AB_2241126
Mouse Monoclonal anti-Amyloid Beta (N) (82E1) Aβ Anti-Human	IBL-America	Cat# 10323; RRID:AB_10707424
Mouse Monoclonal anti-Phospho-Tau (Ser202, Thr205) - AT8	Thermo Fisher Scientific	Cat# MN1020; RRID:AB_223647
Mouse Monoclonal anti-phospho Ser129 alpha-Synuclein	Prothena	Cat# 11A5
Rabbit Monoclonal anti-Alpha-synuclein (phospho S129) [EP1536Y]	Abcam	Cat# ab51253; RRID:AB_869973
Rabbit Monoclonal anti-Gapdh	Cell Signaling	Cat# 5174S; RRID:AB_10622025
Rabbit Polyclonal anti-Ionized calcium-binding molecule 1	FUJIFILM Wako Shibayagi	Cat# 019-19741; RRID:AB_839504
Rabbit Polyclonal anti-tyrosine hydroxylase	Millipore	Cat# AB152; RRID:AB_390204
Rabbit Polyclonal anti-Zonula Occludens-1	Thermo Fisher Scientific	Cat# 40–2200; RRID:AB_2533456
Rabbit Polyclonal anti-β-Actin	Abcam	Cat# ab8227; RRID:AB_2305186
Rabbit Polyclonal anti-α-Synuclein	Sigma-Aldrich	Cat# S3062; RRID:AB_477506
Rat Polyclonal anti-dopamine transporter	Millipore	Cat# MAB369; RRID:AB_2190413
Donkey anti-Goat IgG (H + L) Cross-Adsorbed Secondary Antibody, Alexa Fluor™ 647	Thermo Fisher Scientific	Cat# A-21447; RRID:AB_2535864
Donkey Anti-Goat IgG H&L (Alexa Fluor® 488) preadsorbed	Abcam	Cat# ab150133; RRID:AB_2832252
Goat anti-Chicken IgY (H + L) Secondary Antibody, Alexa Fluor™ 488	Thermo Fisher Scientific	Cat# A-11039; RRID:AB_2534096
Goat anti-Rabbit IgG (H + L) Highly Cross-Adsorbed Secondary Antibody, Alexa Fluor™ 488	Thermo Fisher Scientific	Cat# A-11034; RRID:AB_2576217
Goat anti-Mouse IgG (H + L) Highly Cross-Adsorbed Secondary Antibody, Alexa Fluor™ 594	Thermo Fisher Scientific	Cat# A-11032; RRID:AB_2534091
Goat anti-Rat IgG (H + L) Cross-Adsorbed Secondary Antibody, Alexa Fluor™ 647	Thermo Fisher Scientific	Cat# A-21247; RRID:AB_141778
Horse Anti-Mouse IgG Antibody (H + L), Biotinylated	Vector Laboratories	Cat# BP-2000; RRID:AB_2687893
IRDye® 680LT Goat anti-Rabbit IgG Secondary Antibody	Li-Cor Biosciences	Cat# 926-68021; RRID:AB_10706309
IRDye® 800CW Goat anti-Mouse IgG Secondary Antibody	Li-Cor Biosciences	Cat# 926–32210; RRID:AB_621842
Bacterial strains		
E. coli C600	UMich (M. Chapman)	N/A
E. coli LSR6 (C600:Δ*csgDEFG*; Δ*csgBA*)	UMich (M. Chapman)	N/A
Chemicals, peptides, and recombinant proteins		
SuperScript™ III Reverse Transcriptase	Invitrogen	18080093
iQ™ SYBR® Green Supermix	BioRad	1708880
Thioflavine S	Sigma-Aldrich	T1892

(*Continued on next page*)

Continued		
REAGENT or RESOURCE	SOURCE	IDENTIFIER
Critical commercial assays		
Qiagen RNeasy Plus Universal Mini Kit	Qiagen	73404
S100A8/S100A9 (Calprotectin) ELISA kit	Immundiagnostik AG	K6936
Proteome Profiler Mouse Cytokine Array Kit, Panel A	R&D Systems	ARY006
Deposited data		
16S rRNA gene amplicon sequencing raw data	This paper	PRJEB51988
Experimental models: Organisms/strains		
Mouse: B6.D2-Tg(Thy1-SNCA)14Pjk	Roche Pharma (through MTA)	N/A
Oligonucleotides		
csgA forward primer:: F 5′-GCG-TGA-CAC-AAC-GTT-AAT-TTC-CA-3′	N/A	N/A
csgA reverse primer: R 5′-CAT-ATT-CTT-CTC-CCG-AAA-AAA-AAC-AG-3′	N/A	N/A
csgB forward primer: F 5′-CCA-TCG-GAT-TGA-TTT-AAA-AGT-CGA-AT-3′	N/A	N/A
csgB reverse primer: R 5′-AAT-TTC-TTA-AAT-GTA-CGA-CCA-GGT-CC-3′	N/A	N/A
Snca forward primer: F 5′-GAT-CCT-GGC-AGT-GAG-GCT-TA-3′	N/A	N/A
Snca reverse primer: R 5′-CT-TCA-GGC-TCA-TAG-TCT-TGG-3′	N/A	N/A
SNCA forward primer: F 5′-AAG-AGG-GTG-TTC-TCT-ATG-TAG-GC-3′	N/A	N/A
SNCA reverse primer: R 5′-GCT-CCT-CCA-ACA-TTT-GTC-ACT-T-3′	N/A	N/A
Gapdh forward primer: F 5′-TGC-GAC-TTC-AAC-AGC-AAC-TC-3′	N/A	N/A
Gapdh reverse primer: R 5′-CTT-GCT-CAG-TGT-CCT-TGC-TG-3′	N/A	N/A
E. coli forward primer: F 5′-CAT-GCC-GCG-TGT-ATG-AAG-AA-3′	Smati et al.[113]	N/A
E. coli reverse primer: R 5′-CGG-GTA-ACG-TCA-ATG-AGC-AAA-3′	Smati et al.[113]	N/A
E. coli Taqman probe: FAM-TAT-TAA-CTT-TAC-TCC-CTT-CCT-CCC-CGC-TGA-A	Smati et al.[113]	N/A
E.coli gBlock: GGGCGCAAGCCTGATGCAGCCATGCCGCGTGTATGA AGAAGGCCTTCGGGTTGTAAAGTACTTTCAGCGGGGA GGAAGGGAGTAAAGTTAATACCTTTGCTCATTGACG TTACCCGCAGAAGAAGCACCGGCTAAC	Integrated DNA technology (IDT)	234995349
16S rRNA Universal Prokaryotes forward primer: F 5′-CGG-TGA-ATA-CGT-TCY-CGG-3′,	Bacchetti De Gregoris et al.[114]	N/A
16S rRNA Universal Prokaryotes reverse primer: R 5′-AAG-GAG-GTG-ATC-CRG-CCG-CA-3′	Bacchetti De Gregoris et al.[114]	N/A
16S rRNA Universal Prokaryotes Taqman probe: Cy5-CTT-GTA-CAC-ACC-GCC-CGT-C	Bacchetti De Gregoris et al.[114]	N/A
16S rRNA Universal Prokaryotes gBlock: AGTAATCGTGGATCAGAATGCCACGGTGAATAC GTTCCCGGGCCTTGTACACACCGCCCGTCACA CCATGGGAGTGGGTTGCAAAAGAAGTAGGTAG CTTAACCTTCGGGAGGGCGCTTACCACTTTGTG ATTCATGACTGGGGTGAAGTCGTAACAAGGTAA CCGTAGGGGAACCTGCGGTTGGATCACCTCCT TACCTTAAAGAAGCGTACTTTGCAGTG	Integrated DNA technology (IDT)	234995351

(*Continued on next page*)

Continued		
REAGENT or RESOURCE	SOURCE	IDENTIFIER
Software		
ImageJ	Schneider et al.[115]	https://ImageJ.nih.gov/ij/
R/Bioconductor	R Core Team	https://www.r-project.org/
Image Lab software	BioRad	https://www.bio-rad.com/en-lu/product/image-lab-software?ID=KRE6P5E8Z
Other		
Teklad Costum Diet (Fiber deprived diet)	Envigo	TD.130343 (MD.130434)
Reusable Feeding Needles, 20G	Fine Science Tools	18060–20
EDTA K3 coated blood collection tubes	Sarstedt	41.1504.005
Potter-Elvehjem PTFE pestle and glass tube	Sigma-Aldrich	P7734

RESOURCE AVAILABILITY

Lead contact

Further information and requests for resources and reagents should be directed to and will be fulfilled by the lead contact, Paul Wilmes (paul.wilmes@uni.lu).

Materials availability

In this study no new materials have been generated.

Data and code availability

The 16S rRNA gene amplicon sequencing raw data can be accessed at https://www.ebi.ac.uk/ena/browser/home under the accession number **PRJEB51988**. This paper does not report original code. Access to all other information can be granted upon reasonable request with the lead contact under paul.wilmes@uni.lu.

EXPERIMENTAL MODEL AND SUBJECT DETAILS

Ethical Approval

All animal experiments were approved by the Animal Experimentation Ethics Committee of the University of Luxembourg and the appropriate Luxembourg governmental agencies (Ministry of Health and Ministry of Agriculture) and registered under LUPA 2020/25. Additionally, all experiments were planned and executed following the 3R guidelines (https://www.nc3rs.org.uk/the-3rs) and the European Union directive 2010/63/EU.

Mice

The transgenic line B6.D2-Tg(Thy1-SNCA)14Pjk[34,116] was used, referred to as Thy1-Syn14 or TG from here on forth. This line overexpresses wild-type human αSyn under the transcriptional regulation of the neuron specific Thy1 promoter. As control animals, wild-type (WT) littermates were used, and all TG were heterozygous for the transgene. All mice used were male, since we observed an unexplained attrition of around 25% of female transgenic mice (untreated) between 4 and 5 months of age. For the characterization of the line, different cohorts were used. For the experimental challenge cohort, 72 male animals, 36 TG and 36 WT littermates, were used. They were singly-caged to avoid coprophagy, had access *ad libitum* to food and water and were exposed to a regular 12h-day-night cycle. Animals were monitored twice a week. According to welfare guidelines, humane endpoints were set based on different physical parameters, e.g., weight loss/gain, body temperature and coat condition. At the end of the in-life phase, the mice were anesthetized with a mix of 150 mg/kg ketamine + 1 mg/kg medetomidine and subsequently transcardially flush-perfused with 1X PBS. Prior to perfusion, blood was collected from the right atrium.

During the in-life phase of the study, 10 mice were either found dead in their home cage or reached a humane endpoint (Figure S9A). This measure was in line with the animal welfare guidelines.

METHOD DETAILS

Experimental design

For the challenge study, 9-months old male mice were randomly assigned to 10 different treatment and respective control groups (Figure S9A) and treated for a total of 9 weeks; 1-week dietary priming of the colon and additional 8 weeks combined diet and bacterial challenges (Figure S9B). Food, which was isocaloric (fiber-rich diet: 3.59 kCal/g; fiber-deprived diet: 4 kCal/g), was replaced

every other week and the mice were gavaged with the respective bacteria or sham solution weekly. Body weight and overall health was checked twice a week. Stool samples for microbiome analysis and monitoring of basis gross motor functions via hindlimb clasping and grip strength was also performed weekly (Figure S9B). After euthanasia, blood, brains and colons for molecular biology and histology were collected.

Bacterial solution preparation and gavage

The *E. coli* strains used for treatment were C600 (EC) and its isogenic curli-operon knock-out C600:ΔcsgDEF; ΔcsgBA (ΔEC).[25,27] Both strains were a kind gift from Matthew Chapman, University of Michigan. Expression/absence of curli operon was tested via PCR using the following primer pairs: *csgA*_F-5′-GCG-TGA-CAC-AAC-GTT-AAT-TTC-CA-3′, *csgA*_R-5′-CAT-ATT-CTT-CTC-CCG-AAA-AAA-AAC-AG-3’; *csgB*_F-5′-CCA-TCG-GAT-TGA-TTT-AAA-AGT-CGA-AT-3′, *csgB*_R-5′-AAT-TTC-TTA-AAT-GTA-CGA-CCA-GGT-CC-3’. Additionally, curli protein expression was confirmed by Congo red staining (not shown). Both strains were grown in Lennox broth under aerobic (5% CO_2) conditions at 37°C agitating at 300rpm. Bacteria were resuspended in sterile PBS for oral administration at 10^{10} CFU/mL

They were gavaged with 100μL of bacterial solution, or PBS for the gavage control groups, at a total bacterial load of 10^9CFUs. Reusable stainless steel 20G feeding needles (Fine Science Tools, 18060-20) were used. Prior and in-between gavages, the feeding needle was washed with filtered 70% ethanol and rinsed with sterile PBS. One group of feeding needles per treatment was used to avoid cross-contamination. Importantly, the mice were not pre-treated with an antibiotic mix because 1) antibiotics have been shown to prevent αSyn aggregation and be neuroprotective,[117] and 2) measuring the impact of the fiber deprivation on a native microbiome was an essential readout.

Tissue collection and preparation

Prior to the transcardial perfusion, up to 400μL of venous blood from the right atrium were collected in EDTA K3 coated collection tubes (41.1504.005, Sarstedt), for plasma cytokine measurements. The tubes were gently inverted and then kept on ice. Plasma was collected after centrifugation at 2000 x g for 10 min, transferred to RNase-free tubes and stored at −80°C.

After perfusion, the brain was placed on ice, and split along the longitudinal fissure into two hemibrains. For molecular biology analyzes, one hemibrain was dissected into different regions of interest (striatum and ventral midbrain). The dissected regions were then put on dry ice and stored at −80°C. The other hemibrains were fixed for immunohistochemistry in 4% PBS-buffered paraformaldehyde (PFA) for 48 h at 4°C and then stored in PBS-azide (0.02%) at 4°C. Then, they were cut to generate 50μm thick free-floating sections using the Leica vibratome VT1000 (Wetzlar, Germany), and sections were stored in a 1% (w/v) PVPP +1:1 (v/v) PBS/ethylene glycol anti-freeze mix at −20°C until staining.

Colon samples for mucus measurements and histopathology were fixed in methacarn (60% absolute methanol: 30% chloroform: 10% glacial acetic acid) solution for 2–4 h, then transferred to 90% ethanol and kept at 4°C. Next, whole colon samples were first transversally cut by hand with a microtome blade into 4-5mm long sections. Those pre-cut sections were then put into a histology cassette while respecting the proximal to distal order. They were held in place in an ethanol-soaked perforated sponge. After 24h post-fixation in 10% formalin, the samples were processed in a vacuum infiltration processor. Finally, all samples were embedded in paraffin, and cut at 3μm on a microtome. If not processed immediately, the slides were stored at 4°C before being stained.

RNA extraction and RT-qPCR for midbrain and striatal αSyn characterization

From a separate cohort of 9-months old Thy1-Syn14 mice and corresponding WT littermates, we extracted RNA from dissected colon and different brain regions using the Qiagen RNeasy Plus Universal Mini Kit (Qiagen, 73404). Briefly, 900μL QIAzol lysis buffer (Qiagen, 79306) and three cold 5mm steel balls were added to each sample (previously stored at −80°C). In ice cooled racks, samples were homogenized at 20Hz for 2mins using the Retsch Mixer Mill MM400. Homogenates were transferred to new RNase clean 2mL tubes and left to rest for 5 mins at room temperature (RT). 100μL gDNA eliminator solution was added and the tubes were shaken vigorously for 15 s. Then 180μL of chloroform was added and another strong shake was applied for 15 s. Homogenates were left to incubate for 3 mins at RT. Samples were then centrifuged at 12000 x *g* for 15 min at 4°C. Five hundred μL of the upper aqueous phase was collected and transferred to new 2mL RNase free tubes. Five hundred μL of ethanol was added to the supernatant and mixed by inverting the tubes back and forth. RNeasy mini spin columns were then loaded with 500μL of the mix, centrifuged at 8000 x *g* for 30 s at RT, followed by discarding the flow-through from the collection tube. This step was repeated once more. The spin columns were then washed with two different buffers in three steps: one time with 700μL of RWT buffer and twice with 500μL of RPE buffer. At each washing step, the columns were centrifuged at 8000 x *g* for 30 s at RT, and the flow-through was discarded. Columns were then transferred to new collection tubes and spun at maximum speed for 1min. Finally, columns were transferred to an RNase free 1.5mL Eppendorf tubes. Fifty μL of RNase-free water was added to the columns to elute total RNA. RNA purity and quantity were checked by spectrophotometry using the NanoDrop 2000 (ThermoFisher Scientific) and the Agilent 2100 Bioanalyzer, respectively. Finally, RNA samples were stored at −80°C.

The model used in this study, Thy1-Syn14, carries a transgene for wild-type human αSyn (*SNCA*). To determine the levels of transcript expression in comparison to endogenous murine αSyn (*Snca*), quantitative RT-PCR was performed on a separate untreated age-matched male cohort (N = 15), using the following primer pairs: Snca F 5′-GAT-CCT-GGC-AGT-GAG-GCT-TA-3′, R 5′-CT-TCA-GGC-TCA-TAG-TCT-TGG-3′, SNCA F 5′-AAG-AGG-GTG-TTC-TCT-ATG-TAG-GC-3′, R 5′-GCT-CCT-CCA-ACA-TTT-GTC-ACT-T-3′

and reference gene *Gapdh* F 5′-TGC-GAC-TTC-AAC-AGC-AAC-TC-3′, R 5′-CTT-GCT-CAG-TGT-CCT-TGC-TG-3’. For the reverse transcription of RNA to cDNA the SuperScript III RT reverse transcriptase from Invitrogen was used. Briefly, 1μL of oligo (dT) 20 (50μM) and 1μL of 10mM dNTP mix was added to 1μg of total RNA. If needed, nuclease free water was added to obtain the final reaction volume of 13μL. The mixture was briefly centrifuged for 2-3s, heated at 65°C for 5 min, and again chilled on ice for at least 1 min. Another mixture of 4μL 5× first strand buffer, 1μL RNaseOUT (RNase inhibitor), 1μL of 0.1M DTT, and 1μL of Superscript reverse transcriptase (200 U/μL), was added. The final mixture was briefly centrifuged and incubated at 50°C for 1h followed by 15 mins at 70°C for enzyme deactivation. 80μL of RNase free water were added to the reaction mixture. The obtained cDNA was then placed on ice for immediate use, or stored at −20°C for future use.

The qPCR reaction mix contained 2μL of cDNA, 10μM forward and reverse primers, 1X iQ SYBR Green Supermix (Bio-Rad) and PCR grade water up to a volume of 20μL. Each qPCR reaction was run in duplicates on a LightCycler 480 II (Roche). The thermo cycling profile included an initial denaturation of 3 min at 95°C, followed by 40 cycles at 95°C for 30 s, 62°C (annealing) for 30 s and 72°C (elongation) for 30 s, with fluorescent data collection during the annealing step. Data acquisition was performed by LightCycler 480 Software (version 1.5.0.39).

Protein extraction and western blot

For the mouse line characterization, where we used a separate cohort of 9-months old Thy1-Syn14 mice and corresponding WT littermates, total protein was extracted as described before[118,119] with minor adaptations. Briefly, fresh frozen (stored at −80°C) dissected dorsal striatal and midbrain tissue was homogenized in a lysis buffer solution using a potter (Potter-Elvehjem PTFE pestle and glass tube, Sigma-Aldrich, P7734). The lysis buffer volumes were adapted based on sample size (midbrain: 400μL; dorsal striatum: 800μL). For protein extractions from colon samples of our treatment cohort mice, we used fresh-frozen samples and mechanically disrupted them with a potter (Potter-Elvehjem PTFE pestle and glass tube, Sigma-Aldrich, P7734) and homogenized for 30s with an electric pestle in 200μL of 0.5% SDS in PBS. Samples were spun at 750G for 15minutes to remove cellular debris.

From the characterization cohort, midbrain and striatal samples at 20μg of total protein and 5μL molecular weight standard (Biorad, Dual Color Precision plus (1610374)) were separated on 12% acryl SDS gels and transferred to a Nitrocellulose 0.45μm membrane (Biorad, Trans-Blot Turbo Transfer, built-in Mixed molecular weight setting). For total αSyn, the polyclonal rabbit anti-pan-αSyn antibody (S3062, Sigma-Aldrich, 1:1000) was used, and, as internal reference, a monoclonal mouse anti-α-tubulin antibody (clone [DM1A], ab7291, Abcam, 1:1000), followed by goat anti-mouse IRDyeR 800 CW (Li-Cor Biosciences, 926–32210, 1:10000) and goat anti-rabbit IRDyeR 680LT donkey anti-rabbit (Li-Cor Biosciences, 926–68021, 1:10000) incubated for 1 h at RT incubation.

For the immunoblot detection of zonula occludens-1 and occludin, 10μg of proteins, and 5μL molecular weight standard (Biorad, Dual Color Precision plus (1610374)) were separated on 12% acryl SDS gels and transferred to a Nitrocellulose 0.45μm membrane (Biorad, Trans-Blot Turbo Transfer, built-in Mixed molecular weight setting). Membranes were washed 3 × 5 min in PBS-Tween 0.1% and blocked for 2 h RT in 5% non-fat milk diluted in the washing buffer. Membranes were then incubated overnight under gentle agitation with primary antibodies monoclonal mouse anti-occludin (33–1500, Invitrogen, 1:2000) and the loading control polyclonal rabbit anti-β-actin (ab8227, Abcam, 1:1000), or with the polyclonal rabbit anti-zonula occludens-1 (1:1000; Invitrogen 40–2200) and its specific loading control monoclonal rabbit anti-Gapdh (5174S, Cell signaling, 1:1000) diluted in the blocking solution. After being rinsed 3 × 10 min, membranes were incubated with anti-mouse 800CW (926–32210, Li-Cor Biosciences, 1:10000) and anti-rabbit 680LT (Li-Cor Biosciences, 926–68021, 1:10000) diluted in the blocking solution and washed twice for 5 min.

All protein supernatants were dosed using Bradford’s method assay (Biorad, 5000006). All images were captured using the LI-COR Bioscience C-Digit Chemoluminescence scanner. Subsequent analysis was performed on ImageJ.

Microbial DNA extraction and 16S rRNA gene amplicon sequencing

For microbial DNA extraction from single fecal pellets, an adapted version of the IHMS protocol H[120] was used. Fecal samples were preserved in 200μL of a glycerol (20%) in PBS solution and stored at −80°C. Prior to the extraction, the samples were slightly thawed and added 250μL guanidine thiocyanate and 40μL N-lauryl sarcosine (10%). The samples were then left at RT to fully thaw. Then, 500μL N-lauryl sarcosine (5%) were added before the fecal pellet was scattered and vortexed to homogeneity. Samples were then shortly spun down and incubated at 70°C for 1h. Seven hundred fifty μL pasteurized zirconium beads were added to the tubes, then put in pre-cooled racks and horizontally shaken for 7.5 mins at 25Hz in a Retsch mixer mill MM400. Fifteen mg polyvinylpyrrolidone (PVPP) was added and vortexed until dissolved. Then the samples were centrifuged at 20814 x g for 3mins. The supernatants were transferred to new 2mL tubes and kept on ice. The pellet was washed with 500μLTENP (Tris, EDTA, NaCl and PVPP) and centrifuged at 20814 x g for 3mins. This step was repeated three times in total, and each supernatant was added to the previously new 2mL tube. To minimize carryover, the tubes were centrifuged again at 20814 x g for 5mins, and the supernatant was split equally in two new 2mL tubes. One mL isopropanol (Merck) was added to each tube, and mixed in by inverting the tubes. After a 10min incubation at RT, the samples were centrifuged at 20814 x g for 15mins. The supernatant was discarded, and the remaining pellet air dried under the fume hood for 10mins. The pellet was then resuspended in 450μL phosphate buffer and 50μL potassium acetate by pipetting up and down, before the duplicates were pooled and incubated on ice for 90mins. Then, the sample was centrifuged (20814 x g) at 4°C for 35mins, the supernatant transferred into a new tube. Next, 2μL of RNase (10 mg/ml) were added. Then, the tube was vortexed, briefly centrifuged, and finally incubated at 37°C for 30mins. Then, 50μL of sodium acetate, and 1mL of ice-cold 100% ethanol (Merck) were added, and mixed in by inverting the tube several times. The sample was again incubated at RT for 5mins, and

centrifuged at 20814 x g for 7.5min. The supernatant was discarded, and the newly formed pellet was subsequently washed three times with 70% ethanol (Merck), and centrifuged at 20814 x g for 5mins. The supernatant was discarded each time. Finally, the clean pellet was dried at 37°C for 15 min, then resuspended in 100 μL TE Buffer, and homogenized by pipetting. After incubation at 4°C overnight, DNA quality and quantity were checked by Nanodrop 2000/2000c and Qubit 2.0 fluorometer (Thermo Fischer Scientific). Samples were stored at −80°C until sequencing.

Five ng of isolated gDNA was used for PCR amplification using primers (515F (GTGBCAGCMGCCGCGGTAA) and 805R (GACTACHVGGGTATCTAATCC)) specific to the V4 region of the 16S rRNA gene. For the first round of PCR, samples were amplified for 15 cycles to avoid over-amplification. Six additional PCR amplification cycles were performed in the second round to introduce sample specific barcode information. All samples were pooled in equimolar concentration for sequencing. Sample preparation and sequencing were performed at the Luxembourg Center for Systems Biomedicine (LCSB) Sequencing platform using v3 2x300 nucleotide paired end sequencing kit for MiSeq.

Sample sizes per week:

	WT					TG				
Week	FD PBS	FR ΔEC	FR EC	FD ΔEC	FD EC	FD PBS	FR ΔEC	FR EC	FD ΔEC	FD EC
0	4	8	7	7	8	4	8	9	8	8
1	3	6	7	6	6	3	8	8	8	8
2	4	8	6	8	7	3	7	8	7	8
5	4	8	7	5	7	4	6	7	8	8
6	4	8	7	6	7	4	6	7	8	8
9	4	8	7	5	5	3	5	6	6	6

16S rRNA gene amplicon sequence analysis

Sequence analysis

Amplicon Sequence Variants (ASVs) were inferred from 16S rRNA gene amplicon reads using the dada2 package[121] following the paired-end big data workflow (https://benjjneb.github.io/dada2/bigdata_paired.html, accessed: September, 2020), with the following parameters: truncLen = 280 for forward, 250 for reverse reads, maxEE = 3, truncQ = 7, and trimLeft = 23 for forward, 21 for reverse reads. The reference used for taxonomic assignment was version 138 of the SILVA database (https://www.arb-silva.de).[122]

Microbial diversity and related statistics

Microbiome count data was managed using the phyloseq R package.[123] This package was also used to calculate the Shannon index for alpha diversity and the Bray-Curtis dissimilarity based non-metric multidimensional scaling (NMDS) ordination for beta diversity. Statistical significances of alpha diversity differences were evaluated with the Kruskal-Wallis test (overall comparison between all groups) and the Wilcoxon Rank-Sum Test with false discovery rate correction for multiple comparisons (pairwise contrasts). For beta diversity comparisons, the adonis PERMANOVA test from the R package vegan (Oksanen et al., 2020)[124] was used. All diversity comparisons were performed using ASV count data rarefied to the lowest number of sequences in a sample. Taxon-specific plots (genus and family level) were made using relative abundances (% of taxa out of total).

E. coli quantitative Real-Time PCR (qRT-PCR)

DNA extracted from the mice at weeks 0 (Baseline), 1, 2, 5, 6 and 9, were subjected to qRT-PCR to determine the presence and abundance of *E. coli*. To achieve this, specific primer-probe sets were designed based on *a priori* knowledge of target sequences for the gavaged *E. coli* wildtype strain and a universal 16S rRNA gene. The sequences and the respective primer-probe sets are listed in the key resource table. GBlocks from IDT were used as positive controls, along with DNA extracted from the *E. coli strain C600* that was used for gavaging the mice. Negative controls were established using DNA extracted from *Fusobacterium nucleatum* and *Clostridium sciendens*. No template controls were additionally used to assess any noise from primer-dimers. The levels of *E. coli* were obtained by normalised Cq value based on the universal primer-probe set to account for the total bacterial load within each mouse.

Calprotectin ELISA

We measured calprotectin using the S100A8/S100A9 ELISA kit (#K6936, Immundiagnostik AG, Germany). In brief, fecal samples, collected at week 1, 2, 6 and 9, as well as fecal matter from the proximal colon after the mice were sacrificed, were homogenized 1:10 (w (mg)/v (μL)) in extraction buffer by vortexing and subsequently centrifuged for 10 min at 3000 × g. We transferred the supernatant to a new microcentrifuge tube and used 100μL for ELISA assay. We then followed the protocol as per the provider. The data was analyzed using the 4-parameter algorithm.

Plasma cytokine measurements

Blood was collected as described above using K3 EDTA plasma tubes (41.1504.005, Sarstedt). After collection, tubes were inverted up to 4 times. Plasma was isolated by centrifugation at 2000 x g for 10 min. We then transferred supernatants to new microcentrifuge tubes and stored them at −80°C until further use.

For the cytokine measurements, we diluted the samples 1:10 in PBS. For the measurement we used a membrane-based antibody array (ARY006, R&D Systems) and followed the provider's protocol.

In brief, the membranes are first incubated for 1hr on a horizontal platform shaker in blocking buffer. During incubation we prepared the samples by adding our diluted (1:10) plasma to 0.5 mL of array buffer 4 and added 15 μL of the Mouse Cytokine Array Panel A Detection Antibody Cocktail to the samples. The incubation was kept for 1 h at RT. After this blocking step, we removed the blocking buffer, added the sample mix onto the membranes and maintained the incubation overnight at 4°C on a horizontal platform shaker. Finally, we washed the membranes twice and then added 2mL of a Streptavidin-HRP solution onto the membranes and incubated them for 30 min. We washed the membranes again as before, then put the membranes between a sheet where we added 1mL of Chemi Reagent Mix. All air bubbles were smoothed out and incubation was performed for 1 min. Excess buffer was removed, the cover was reapplied and finally proteins of interest were revealed by chemiluminescence on a ChemiDoc XRS+ System (Bio-Rad, Belgium). For the analysis of the signals, we used the Image Lab software (version 6.1.0 build 7) from Bio-Rad. We created a mask, which was applied on each membrane to cover the same surface for each dot/protein (area fixed at 3.34 mm^2). Pixels intensity (mean value) of each cytokine was then established per membrane.

Alcian blue staining and outer mucus thickness measurements

We performed a first Alcian blue staining on a separate study cohort, which we challenged with the FD diet for either 1 week or 3 weeks. This staining was performed at the University of Michigan and microphotographs were taken at the Laboratoire National de Santé (LNS) in Dudelange, Luxembourg. The Alcian blue stainings on the treatment cohort colon samples were performed at the National Center of Pathology (NCP) of the Laboratoire National de Santé in Dudelange (Luxembourg). The sections were stained for Alcian blue (Artisan Link Pro Special Staining System, Dako, Glostrup, Denmark) according to manufacturer's instructions.

Five-10 images per section at 20x magnification were collected for each mouse. This resulted in up to 24 images per animal. The criterion for the correct images was that the sections were cut at the correct plane level. This was determined by the orientation and definition of the colonic crypts, which had to be fully visible pointing toward the colonic lumen. Only the outer mucus areas which could clearly be distinguished from the inner mucus layer and the colonic content were measured. In ImageJ, the scale of each image was adapted using the imprinted scale bar as reference and an average of 6 measure points, spanning the outer mucus layer, per image were taken. The criteria as well as the analysis for the inner mucus measures were identical.

Immunofluorescent staining of colon sections

Sections were deparaffinized in xylene 3 × 5mins. Before proceeding to rehydration, the slides were checked for paraffin residues. If not, the sections were treated another round with xylene. A three-step rehydration with 100%, 70% and 50% ethanol each twice for 10 min followed deparaffinization. After washing with dH_2O, slides were treated with a citrate buffer (0.1M, pH6.0, +0.1% Tween 20) for antigen retrieval at 80°C for 30-35mins. After letting the sections cool down for 20mins, the slides were again washed with dH_2O 2 × 5mins. Next, endogenous peroxidase activity was quenched by incubating the slides in a 3% H_2O_2 methanol solution for 15 min, followed by tissue permeabilization in PBS +0.4% Triton X-100 (PBS-T) + 1% BSA (2 × 10mins). Finally, unspecific antigen binding was blocked by incubation with PBS-T + 5% BSA for 30–45 min. After washing again with dH_2O, the tissue was circled with a hydrophobic Dako pen (S2002, DAKO), and the primary antibodies were added. They were incubated at room temperature (RT) for 2hrs, and then transferred to 4°C for overnight incubation in a humidified chamber. The following day, slides were washed briefly with dH_2O, then washed with 1% BSA + PBS-T 2 × 5mins, and finally rinsed with dH_2O. Tissues were circled again with the hydrophobic pen and secondary antibodies were added. Slides were then incubated for 2 hrs at RT in the humidified chamber. Finally, they were washed 3 × 5mins with PBS-T, and rinsed with dH_2O. Excess water was removed by gentle tapping, and slides were coverslipped with DAPI Fluoromount-G (0100-20, SouthernBiotech).

To detect phosphorylated αSyn in the ENS, double staining using the following antibodies: polyclonal chicken anti-PGP9.5 (ab72910, Abcam; 1:1000), monoclonal rabbit anti-pS129-αSyn (ab51253, Abcam; 1:500) was used.

For the localization of total and human αSyn in the colon, primary antibodies used were a polyclonal rabbit anti-pan-αSyn (S3062 Sigma-Aldrich, 1:1000) and a monoclonal mouse anti-human-αSyn (clone 211, S5566, Sigma-Aldrich, 1:1000). The protocol was the same except for the revelation steps. Briefly, after primary antibody incubation, slides were incubated with the appropriate secondary biotinylated antibody for 2 h. Next, they were incubated with an avidin-biotin-complex mix for 1 h. After washing 2 × 10mins in PBS-T, the revelation was done using 3,3′-diaminobenzidine (DAB) and H_2O_2 for 3mins. The slides were finally washed in twice ddH2O for 2mins each and again once in dH2O for 2mins. As counterstain, the slides were emersed 2 times in hematoxylin for 20s and washed in dH2O. A final rinse under tap water was done to intensify the colorization. All slides were again dehydrated (70% EtOH, 95% EtOH and Xylene) and coverslipped using Merck's Neo-Mount.

Behavior

Hindlimb clasping

The method was adapted from.[125] In brief, animals were taken by the tail near the base and suspended for 10 s. If both hindlimbs stayed stretched and did not touch the abdomen for more than 50% of the suspension time, the mouse was scored 0. A score of 1 or 2 was given if one respectively both hindlimbs were retracted for more than 50% of the suspension time. If they were retracted and touched the abdomen for the entire suspension time, a score of 3 was given. In the most severe cases, the animals twisted around the vertical body axis or even rolled up to a so-called bat position. These cases were given a score of 4. This test was repeated weekly.

Grip strength

The grip strength test[126] was performed using Bioseb's grip strength meter (Vitrolles, France). This test was chosen for the challenged cohort, since we expected the animals to vary in weight due to a potential diet impact. Weight fluctuations would have introduced an unwanted bias in the inverted grid hanging test (see below). Animals were gently placed on a grid, allowed to grab onto it with all four paws, and then gently pulled off in a continuous backwards motion by their tail. Technical triplicates were taken for each mouse. Values were normalized to the weight of the respective mouse. The test was repeated weekly.

For the characterization of the Thy1-Syn14 line in different aged cohorts (3, 6 and 13 months), we performed a simplified version of the inverted grid test to assess grip strength.[127,128] Therefore, we measured the hanging time to assess the simultaneous 4-limb grip strength, with a time cut-off of 30 s. Since for the same age and genotype, the mice did not vary in weight, no weight altering procedure influenced the weight and there was no external force involved, we did not have to normalize the hanging time values by the respective weights of the mice.

Adhesive removal

The test was adapted from Bouet and colleagues.[129] Briefly, animals were placed in a round transparent arena for 1 min as habituation. A piece of rectangular tape (3 × 5mm) was placed on each forepaw. The time was taken once the animals touched the bottom of the arena. Then, the time of first touch and first removal was taken. The test was performed in duplicates, and performed at baseline as well as at the end of the in-life phase. Replicates from mice that froze for at least 30 s, when we put them into the arena, were removed from the analysis.

Immunofluorescent staining on free-floating brain sections

Immunofluorescent stainings on free-floating sections were performed following a standard protocol[130] with minor adaptations. Briefly, sections were washed in PBS +0.1% Triton X-100 (T_{X100}) to rinse off the anti-freeze solution. Then, they were treated with a permeabilization/peroxidase inactivation solution (PBS +1.5% T_{X100} + 3% H_2O_2) for 30mins, followed by 2 × 5mins washing. To prevent unspecific antibody binding, the sections were incubated in 5% BSA +0.02% T_{X100} for 1 h. After a short washing step, sections were incubated with primary antibody(ies) diluted in antibody solution (PBS +2% BSA) over night at room temperature (RT) on an orbital shaker. The next day, sections were washed with PBS +0.1% TX100 to remove all excess first antibody. Sections were then incubated with secondary antibody (+ antibody solution) for 2 hrs at RT on an orbital shaker under a light trap. Finally, sections were washed with simple PBS (at least three times for 10mins) and then mounted on Superfrost (ThermoFisher Scientific) slides, left to dry for up to 12hrs, and cover-slipped using the Fluoromount-G (Invitrogen) mounting solution.

The following antibodies were used: monoclonal rabbit anti-pS129-αSyn (Abcam, ab51253; 1:1000), monoclonal mouse anti-pS129-αSyn (Prothena Biosciences Inc., 11A5; 1:1000), polyclonal chicken anti-tyrosine hydroxylase (Abcam, ab76442; 1:1000), polyclonal rabbit anti-tyrosine hydroxylase (Merck (Sigma-Aldrich), AB152; 1:1000), polyclonal rat anti-dopamine transporter (MAB369, Merck (Sigma-Aldrich); 1:1000), polyclonal rabbit pan αSyn (S3062, Sigma-Aldrich, 1:1000), monoclonal mouse human αSyn 211 clone (S5566, Sigma-Aldrich, 1:1000) and rabbit anti-ionized calcium-binding adapter molecule 1 (Iba1) (1919741, Wako, 1:1000).

Quantitative neuropathology

Sections were imaged using a Zeiss AxioImager Z1 upright microscope, equipped with a PRIOR motorized slide stage and coupled a "Colibri" LED system to generate fluorescence light of defined wavelengths, and a Zeiss Mrm3digital camera for image capture. The complete imaging system was controlled by Zeiss' Blue Vision software. All histological analyzes were performed blinded.

The quantification of TH-positive fibers and DAT-positive synaptic terminals was done as described before in.[86] Briefly, two doubly labeled (rabbit anti-TH and rat anti-DAT) sections were used. A total of 6 (3/section) 40x (223.8 × 167.7 μm^2) pictures of the dorsal striatum were acquired using the optical sectioning system Apotome.2 (Zeiss). The percent area occupied of TH and DAT by intensity thresholding was determined using ImageJ software and averaged for each mouse.

The quantification of TH-positive neurons in the SNpc has been described and the obtained results were shown to correlate with stereological cell counts (see supplementary information in Ashrafi et al., 2017). Briefly, to estimate TH-positive neurons in the SNpc, anatomically distinguishable levels were identified and applied to 7–12 fifty-micron sections/mouse. Then, 2x2 tiled pictures/section were taken at 10X objective and converted into single Tiff files for image analysis. Next, the region of interest (ROI) of the area occupied only by TH-positive neurons was outlined. After thresholding, the ROI occupied (in pixels) by TH-positive neurons was measured. For each anatomical levels of the SN, up to 2 sections/level were measured. Single and/or averaged values/level were finally summed up to one single representative value, the "cumulative SN surface" and converted to mm^2.

CellPress
OPEN ACCESS

To quantify pS129-αSyn in the dorsal striatum (Double label: area reference marker polyclonal rabbit anti-TH; pS129-αSyn marker monoclonal mouse 11A5), 40x images were converted to 8-bit and the threshold was automatically set to "MaxEntropy". Next, the images were appropriately scaled from pixel to μm. Greater non-synaptic particles were excluded by selecting them using the "Analyze Particles" tool (*Size (μm2): 30.00-Infinity; Circularity: 0.25-1)* and adding them to the ROI Manager. Before measuring the area occupied by pS129-aSyn-positive synapses, all images were again manually curated for wrongly selected areas. Finally, the ROIs to be excluded were selected using the "XOR" tool from the ROI manager. This resulted in a final curated ROI for quantification of the percent area occupied by pS129-αSyn⁻positive synaptic areas.

To estimate pS129-αSyn positive aggregates in the SNpc (monoclonal rabbit anti-pS129-αSyn), the same ROIs as chosen for TH quantification were used. In ImageJ, a virtual grid with a square area of 2500μm^2 was overlaid. Pictures were then manually analyzed for pS129-αSyn positive accumulations. Based on morphology, pS129-αSyn positive cell bodies (1 count = 1 cell body) and other pS129-αSyn positive particles (number of particles per square) of non-cell shapes were counted separately. Finally, counts were normalized per square (area of ROI/area of square) and summed up for all 4 zones (see TH quantification).

To quantify microglia, 3-4 sections for SNpc and 2 sections for the dorsal striatum, were doubly stained for TH and Iba1. For the SNpc, microphotographs were acquired as described above the TH quantification. Due to reduced availability of tissue, we could not acquire image for all different zones as described for TH quantification. We therefore pooled all images and focused on the ventral downward-pointing region of the SNpc which contained TH-positive neurons. In ImageJ, the microphotographs were then overlayed so that the orientation was the same for all images and after thresholding, the Iba1-positive relative area occupied (in %) of the ROI occupied (in pixels) by TH-positive neurons was measured.

Double IF and thioflavin-S staining on free-floating sagittal brain sections

For immuno- and Thioflavine S (Sigma-Aldrich, T1892) double staining, fluorescent immunostaining was performed first, microphotographs were taken, then Thio-S staining was performed as described.[131] Two brain sections/mouse of the following mice or mouse models were used: 2 mice of this study with the highest αSyn inclusion load (for illustration purposes) after FD and E.coli treatment, three 17-month-old transgenic mice overexpressing 4-repeat human tau mutated at G272V and P301S under the Thy1.2-promotor,[132] and three 20-month-old transgenic mice overexpressing mutated human amyloid protein precursor with two mutations linked to familial Alzheimer's disease (Swedish and Indiana mutations) under the PDGF-β promoter.[131] Antibodies used were all mouse monoclonals: 11A5 for pS129-αSyn (Prothena), AT8 for hyperphosphorylated tau (Invitrogen, MN1020), 82E1 for amyloid beta (IBL-America, 10323), and used at 1:200. Secondary antibody was goat anti-mouse AlexaFluor 594 (Invitrogen, A-11032), diluted 1:200.

For illustration of Thio-S signals, the same location (with the help of anatomical landmarks on brain sections) as chosen for antibody signals was chosen (with the help of anatomical landmarks on the sections) for a second microphotograph. Pictures were taken with the same microscope equipment as described above.

QUANTIFICATION AND STATISTICAL ANALYSIS

For the statistics of the 16S rRNA gene amplicon sequencing data see above. For all other measurements of the challenged mouse cohort, the permutational multivariate analysis of variance (PERMANOVA, non-parametric multivariate statistical permutation) test and the Kruskal-Wallis followed by the non-parametric Mann-Whitney U test for group comparison were performed. False discovery rate (FDR) was used to correct for multiple comparison. However, in many cases the uncorrected p values were used to illustrate noteworthy differences. We noted in figure legends and/or the main text, which test(s) was/were used and if, in case of the Mann-Whitney U test, a correction for multiple comparison was applied.

Cell Reports, Volume *42*

Supplemental information

Fiber deprivation and microbiome-borne curli shift gut bacterial populations and accelerate disease in a mouse model of Parkinson's disease

Kristopher J. Schmit, Pierre Garcia, Alessia Sciortino, Velma T.E. Aho, Beatriz Pardo Rodriguez, Mélanie H. Thomas, Jean-Jacques Gérardy, Irati Bastero Acha, Rashi Halder, Camille Cialini, Tony Heurtaux, Irina Ostahi, Susheel B. Busi, Léa Grandmougin, Tuesday Lowndes, Yogesh Singh, Eric C. Martens, Michel Mittelbronn, Manuel Buttini, and Paul Wilmes

SUPPLEMENTAL MATERIAL

Supplementary Figures and Legends

Figure S1 | Alpha-synuclein is expressed in the nigro-striatal pathway of Thy1-Syn14 and wild-type mice

(A) Representative microphotographs for total αSyn (pan-αSyn) in the dorsal striatum (top; scale bar: 50µm) and the substantia nigra pars compacta (SNpc; bottom; scale bar: 200µm) between WT (left) and TG (right) mice in TH-positive regions.

(B) Representative western blot gels targeting total αSyn from the dorsal striatum (top) and midbrain (bottom) in 9 months-old TG and WT animals. The band for αSyn was detected at approximatively 14kDa. For subsequent normalization we used here as reference protein α-tubulin, which was detected at approximatively 50kDa. The gels were loaded randomly with TG and WT total protein extracts.

WT, wild-type littermates; TG, Thy1-Syn14

Figure S2 | Alpha and beta diversity assessment for the different challenges

(A) Boxplots for alpha diversity of the different variables/challenges. Genotype and Diet challenges were both altering alpha diversity. No significant changes were induced by gavage alone. P-values were determined using the Kruskal-Wallis test and were corrected for FDR. Data points: Left | WT = 187, TG = 197; Middle | FR = 172, FD = 212; Right | PBS = 44, ΔEC = 168, EC = 172.

(B) Non-metric multi-dimensional scaling (NMDS) plots for beta diversity of the different variables/challenges. Here diet and gavage led to changes in beta diversity However, one has to consider that the PBS gavaged mice were exclusively FD challenged. The dissimilarities were genotype independent. P-values were determined using the adonis test. Data points: Left | WT = 187, TG = 197; Middle | FR = 172, FD = 212; Right | PBS = 44, ΔEC = 168, EC = 172.

WT, wild-type littermates; TG, Thy1-Syn14; FD, fiber deprived; FR, fiber rich; PBS, phosphate buffered saline solution; ΔEC, curli-KO *E. coli*; EC, wild-type curli expressing *E. coli*

Figure S3 | Fiber-deprivation induced increased Firmicutes to Bacteroidetes ratios

Boxplots illustrating the Firmicutes to Bacteroidetes ratios for each tested time point between FR and FD challenged mice. Both genotypes show significant increases in ratios at 9 weeks. Thy1-Syn14 mice do already show significant differences at week 6. P-values were determined using the Kruskal-Wallis test and were corrected for FDR.

Sample sizes:

Week	WT					TG				
	FD PBS	*FR ΔEC*	*FR EC*	*FD ΔEC*	*FD EC*	*FD PBS*	*FR ΔEC*	*FR EC*	*FD ΔEC*	*FD EC*
**0**	4	8	7	7	8	4	8	9	8	8
**1**	3	6	7	6	6	3	8	8	8	8
**2**	4	8	6	8	7	3	7	8	7	8
**5**	4	8	7	5	7	4	6	7	8	8
**6**	4	8	7	6	7	4	6	7	8	8
**9**	4	8	7	5	5	3	5	6	6	6

WT, wild-type littermates; TG, Thy1-Syn14; FD, fiber deprived; FR, fiber rich; PBS, phosphate buffered saline solution; ΔEC, curli-KO E. coli; EC, wild-type curli expressing E. coli

Figure S4 | Differential taxa abundance over time is diet regulated

(A) Complex heatmap visualizing the effect of different challenges on the relative abundance changes over time. The taxa were subdivided into three different groups according to their relative abundances: High, at least for one time point, there was an average relative abundance of 10% or higher for at least one treatment group; Mid, at least for one time point, there was an average relative abundance between at least 1 and maximum 10% for at least one treatment group; Low, less than 1%. All data was scaled and centered for each row. Main effect is seen between FR and FD challenges. Analogous to what we had seen before, the FD diet challenge was the main driver of microbial abundance changes FD challenged mice had especially higher levels in *Intestinimonas, Odoribacter, Colidextribacter, Rikenellaceae RC9 gut group* and *Akkermansia,* and decreased levels in *Lachnospiraceae NK4A136 group* and *Lactobacillus* over time (see also Figure 2C). *Alistipes, Faecalibaculum, Bacteroides, Lachnoclostridium* and *Lachnospiraceae UCG_006* on the other hand saw fluctuations over time. With the exception of *Faecalibaculum*, they first increased before dropping back down to initial levels in FD challenged mice. Inversely, levels of *Faecalibaculum* first dropped significantly in FD diet challenged mice, before even

surpassing its measured levels in FR diet groups. For some taxa, we observed also difference between genotypes.

(B-D) Boxplots illustrating the quantitative real time PCR results specifically targeting *E. coli.* For clarification: values are inversely related to the amount of *E. coli* in fecal samples.

(B) The FD diet (Kruskal-Wallis, p = 4.55E-12) challenge as well as (C) PBS gavage (Mann Whitney U, FDR = 0.027) leads overall to significantly higher levels of *E. coli* in feces. Data points: Diet | FR = 172, FD = 212; Gavages | PBS = 44, ΔEC = 168, EC = 172.

(D) Boxplots illustrating the different challenge groups for WT (top row) and TG (bottom row) mice separately at different time points (week 0, week 1, week 2, week 5, week 6, week 9). In WT mice we saw significant group differences at Week 1 (Kruskal-Wallis, p = 0.0157) and Week 5 (Kruskal-Wallis, p = 0.0366), whereas in TG animals we saw significant differences between the groups at Week 2 (Kruskal-Wallis, p = 0.00793) and Week 5 (Kruskal-Wallis, p = 0.0267).

Sample sizes:

Week	WT					TG				
	FD PBS	*FR ΔEC*	*FR EC*	*FD ΔEC*	*FD EC*	*FD PBS*	*FR ΔEC*	*FR EC*	*FD ΔEC*	*FD EC*
**0**	4	8	7	7	8	4	8	9	8	8
**1**	3	6	7	6	6	3	8	8	8	8
**2**	4	8	6	8	7	3	7	8	7	8
**5**	4	8	7	5	7	4	6	7	8	8
**6**	4	8	7	6	7	4	6	7	8	8
**9**	4	8	7	5	5	3	5	6	6	6

WT, wild-type littermates; TG, Thy1-Syn14; FD, fiber deprived; FR, fiber rich; PBS, phosphate buffered saline solution; ΔEC, curli-KO *E. coli*; EC, wild-type curli expressing *E. coli*

Figure S5 | Invariant inner mucus thickness and outer mucus to microbial diversity associations

(A) Barplot illustrating the combined mucus thickness from the pilot study in WT and TG mice after 1 week or 3 weeks of FD diet challenge.

(B) Inner mucus thickness does not vary significantly between the different groups. To note is however, the ticker inner mucus layer after 1 week in TG mice on the FD diet compared to its WT equivalent. This could indicate an early and greater compensatory/rescue mechanism in TG

animals. This is not observed anymore after 3 weeks. Sample sizes: 1-Week | WT – FR: n = 3, FD: n = 3; TG – FR: n = 2, FD: n = 3; 3-Week | WT – FR: n = 1, FD: n = 3; TG – FR: n = 2, FD: n = 2.

(C) Outer mucus thickness is, independent of genotype, significantly thinner due to the FD diet challenge after 1 week (Kruskal-Wallis, p = 0.004) and 3 weeks (Kruskal-Wallis, p = 0.036), respectively. Sample sizes: 1-Week | WT – FR: n = 3, FD: n = 3; TG – FR: n = 2, FD: n = 3; 3-Week | WT – FR: n = 1, FD: n = 3; TG – FR: n = 2, FD: n = 2.

(D) Barplot illustrating the combined mucus thickness in all different treatment groups.

(E) Inner mucus thickness does not vary significantly between the different treatment groups in the separate genotypes. To note is however, the significant ticker inner mucus layer in the TG FD EC group compared to its WT equivalent. This inferred a greater compensatory/rescue mechanism in TG animals. Sample sizes: WT FD PBS, n = 3; WT FR ΔEC, n = 5; WT FR EC, n = 4; WT FD ΔEC, n = 3; WT FD EC, n = 7; TG FD PBS, n = 2; TG FR ΔEC, n = 4; TG FR EC, n = 6; TG FD ΔEC, n = 6; TG FD EC, n = 7.

(F - G) Scatterplots of Spearman rank tests comparing alpha diversity (y-axis) and mucus thickness (x-axis) (C) overall, (D) for the different diet groups, and (D) combining genotype and diet groups

WT, wild-type littermates; TG, Thy1-Syn14; FD, fiber deprived; FR, fiber rich; PBS, phosphate buffered saline solution; ΔEC, curli-KO *E. coli*; EC, wild-type curli expressing *E. coli*

Figure S6 | Gross motor and sensorimotor functions are transgene driven

(A - B) Longitudinal gross motor function monitoring visualized in line plots indicating the median value changes for the different treatment groups. Alpha-synuclein overexpression was the driver of motor deficits in transgenic animals. (A) Hindlimb clasping scores increased within all transgenic groups (dotted lines), independent of their treatment. Differences between WT and TG animals were significant from baseline to week 9. All p-values were determined using the Mann-Whitney U test and FDR to correct for multiple comparison. (B) Grip strength results confirm that there is only a difference between the genotype and there was no observable difference due to any kind of treatment. Differences between WT and TG animals were significant from baseline to

week 9. All p-values were determined using the Mann-Whitney U test and FDR to correct for multiple comparisons.

(C) Boxplots illustrating the latency for touch in the different treatment groups (x-axis) for WT (red) and TG (blue) animals after 9 weeks. The time of touch refers to the sensory ability of the mice. Here we saw a clear significance between genotypes (Kruskal-Wallis, corrected for FDR; $p < 0.0001$). More specifically there are significant differences in the FR ΔEC, FR EC, and FD EC between genotypes. Sample size: WT FD PBS, n = 4; WT FR ΔEC, n = 8; WT FR EC, n = 6; WT FD ΔEC, n = 5; WT FD EC, n = 6; TG FD PBS, n = 4; TG FR ΔEC, n = 5; TG FR EC, n = 7; TG FD ΔEC, n = 8; TG FD EC, n = 7.

All p-values were determined using the Mann-Whitney U test.

WT, wild-type littermates; TG, Thy1-Syn14; FD, fiber deprived; FR, fiber rich; PBS, phosphate buffered saline solution; ΔEC, curli-KO *E. coli*; EC, wild-type curli expressing *E. coli*

Figure S7 | Not all immunopositive pathological protein inclusions are Thio-S positive in different models of neurodegeneration.

Left column shows immunostaining of different protein inclusions. Right column shows Thio-S staining of the same location. The two top rows show pS129-αSyn immunostaining and Thio-S staining of the dorsal striatum (top row) and SNpc (ssyond row) of a TG mouse after FD and EC challenges, used in this study. The third row shows a mouse 3 month after intrastriatal injection of αSyn preformed fibrils, as described in one of our previous studies [86]. None of the αSyn inclusions are Thio-S positive, but neurodegeneration was detected in both. The fourth row shows AT8 immunostaining for hyperphosphorylated tau and Thio-S staining of tau inclusions in the hippocampal CA1 of a 17-month-old hTau transgenic mice [134]. The fifth row shows 82E1 immunostaining and Thio-S staining of amyloid plaques in the hippocampal dentate gyrus of a 20-month-old mutated hAPP transgenic mouse [135]. Note that numerous immunopositive structures, in both models, are not positive for Thio-S. Scale bar: 60 μm.

Figure S8 | Minimal or no central and peripheral inflammation in Thy1-Syn14 mice after diet and *E. coli* challenges

(A) Neuroinflammation was assessed using the microglia marker Iba1. Quantification was performed by measuring the area occupied by Iba1-positive microglia in TH-positive regions of the SNpc and the dorsal striatum. No significant changes were observed. Sample size: WT FD PBS, n = 4; WT FR ΔEC, n = 8; WT FR EC, n = 6; WT FD ΔEC, n = 5; WT FD EC, n = 7; TG FD PBS, n = 4; TG FR ΔEC, n = 5; TG FR EC, n = 7; TG FD ΔEC, n = 8; TG FD EC, n = 8.

(B) Boxplots illustrating changes in calprotectin between FR and FD diet challenged animals at different time points. Fecal calprotectin levels were subtle but significantly higher in WT mice at weeks 2 (Kruskal-Wallis, p = 0.025), 6 (Kruskal-Wallis, p = 0.017) and in fecal matter from the proximal colon collected at post-mortem (PM) (Kruskal-Wallis, p = 0.002) and in TG mice at weeks 1 (Kruskal-Wallis, p = 0.02), 2 (Kruskal-Wallis, p = 0.003), 6 (Kruskal-Wallis, p = 0.042) and in fecal matter from the proximal colon collected at PM (Kruskal-Wallis, p = 0.059) fed the FD diet.

Sample sizes:

**Week**	**WT**					**TG**				
	FD PBS	*FR ΔEC*	*FR EC*	*FD ΔEC*	*FD EC*	*FD PBS*	*FR ΔEC*	*FR EC*	*FD ΔEC*	*FD EC*
**1**	2	7	5	3	3	3	4	6	7	6
**2**	2	7	5	4	4	3	4	6	7	7
**6**	1	4	3	0	2	2	3	2	3	4
**9**	4	8	6	5	3	4	5	7	8	8
**PM**	3	7	6	5	5	3	2	6	7	4

(C) At the end of the treatments, plasma levels of many cytokines/chemokines (Ccl1 (Kruskal-Wallis, p = 0.0305), Ccl11 (Kruskal-Wallis, p = 0.0305), Ccl2 (Kruskal-Wallis, p = 0.0388), Ccl3 (Kruskal-Wallis, p = 0.032), Ccl4 (Kruskal-Wallis, p = 0.0184), Ccl5 (Kruskal-Wallis, p = 0.0107), Cxcl1 (Kruskal-Wallis, p = 0.025), Cxcl9 (Kruskal-Wallis, p = 0.0388), G-Csf (Kruskal-Wallis, p = 0.00604), Il2 p70 (Kruskal-Wallis, p = 0.0184), Il16 (Kruskal-Wallis, p = 0.032), Il17 (Kruskal-Wallis, p = 0.0101), Il1a (Kruskal-Wallis, p = 0.0407), Il1b (Kruskal-Wallis, p = 0.0166), Il1ra (Kruskal-Wallis, p = 0.032), Il23 (Kruskal-Wallis, p = 0.032), Il5 (Kruskal-Wallis, p = 0.00173), Il7 (Kruskal-Wallis, p = 0.0446), Trem-1 (Kruskal-Wallis, p = 0.0276)) had overall significantly lower levels in TG than in WT mice. When we looked further into how diet, the gavages or their combination might have impacted the chemo-/cytokine levels, we saw that in mice fed the FD diet protein levels for Ccl3, Ccl5, Cxcl2, Il1ra, Il27 and Il6 were lower as compared to FR diet fed mice. When we further looked into how the challenges affected plasma in the individual genotypes, we

saw that in TG mice fed the FD diet levels for Ccl5 ($p = 0.009$), Cxcl2 ($p = 0.013$) and Il1ra ($p = 0.044$) were significantly lower (not shown in detail). The lowest levels for these proteins, especially for Ccl5 ($p = 0.009$) and Cxcl2 ($p = 0.015$), were observed when TG mice were doubly FD EC challenged. This unexpected finding may be due to the overexpression of human αSyn in immune cells of TG mice, which may lead to their immune suppression. See main text for more details. Sample size: WT FD PBS, n = 3; WT FR ΔEC, n = 5; WT FR EC, n = 6; WT FD ΔEC, n = 5; WT FD EC, n = 6; TG FD PBS, n = 3; TG FR ΔEC, n = 5; TG FR EC, n = 6; TG FD ΔEC, n = 5; TG FD EC, n = 6.

WT, wild-type littermates; TG, Thy1-Syn14; FD, fiber deprived; FR, fiber rich; PBS, phosphate buffered saline solution; ΔEC, curli-KO *E. coli*; EC, wild-type curli expressing *E. coli*; PM, post-mortem collected fecal matter from the proximal colon

Figure S9 | Experimental design and in-life experiments

(A) Hierarchy graphs illustrating the experimental design. See details in Material and Methods. The white on black background numbers refer to the numbers of animals we were left with at the end of the in-life phase.

(B) Graph showing which treatments were given or tests were performed during the in-life phase. Gross motor functions were tested weekly, while the adhesive removal test was only performed twice, at start and end of the in-life phase. Feces were checked weekly and afterwards six time points were chosen for analysis. Mice were gavaged weekly starting at week 2 for a total 8 times and their food was changed every second week

WT, wild-type littermates; TG, Thy1-Syn14; FD, fiber deprived; FR, fiber rich; PBS, phosphate buffered saline solution; ΔEC, curli-KO *E. coli*; EC, wild-type curli expressing *E. coli*

Supplementary Tables and Legends

Table S1 | Comparison of the 16S rRNA sequencing results to known taxa altered in stool samples of patients with PD (adapted from Boertien et al., 2019)

Table S2 | Abbreviations for the main figures

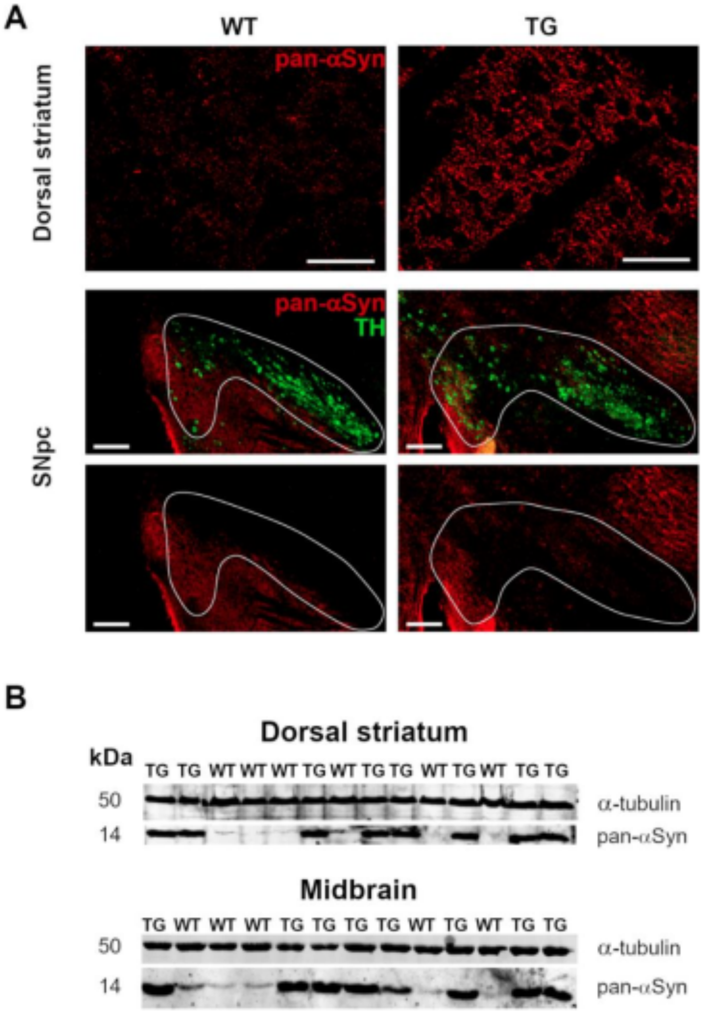

Figure S1 | Alpha-synuclein is expressed in the nigro-striatal pathway of Thy1-Syn14 and wild-type mice

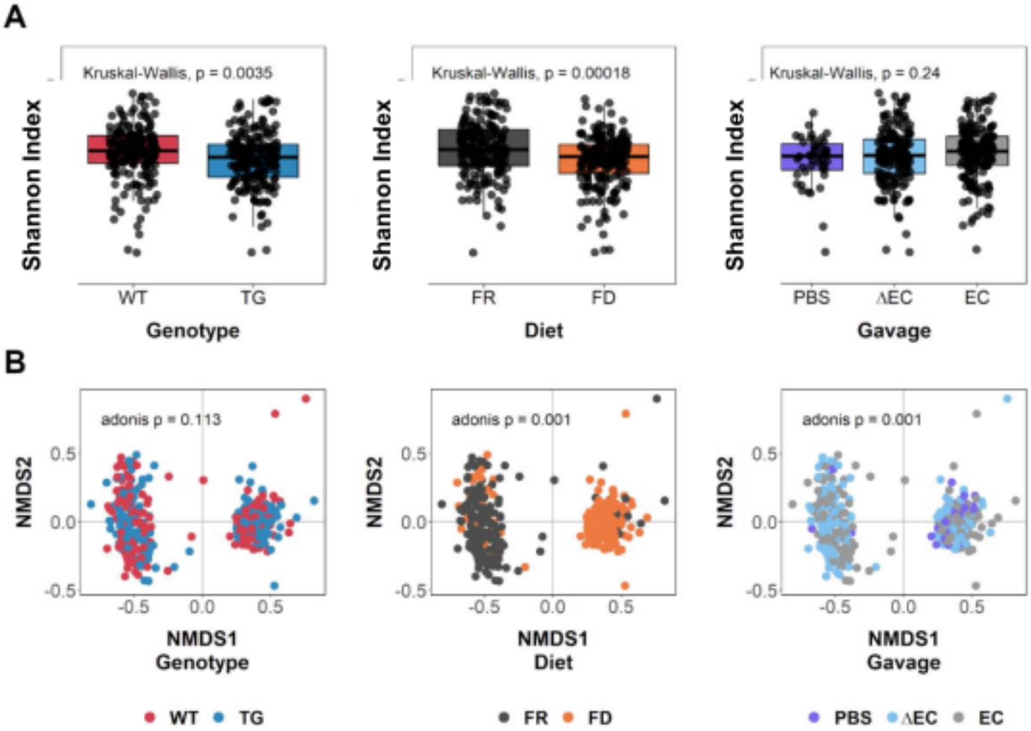

Figure S2 | Alpha and beta diversity assessment for the different challenges

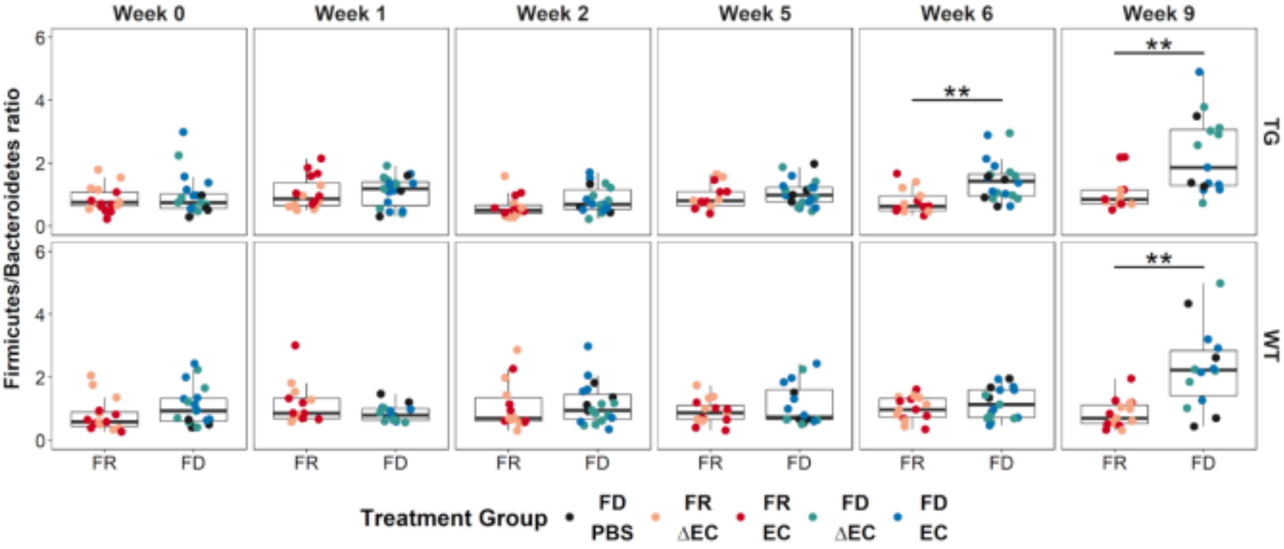

Figure S3 | Fiber-deprivation induced increased Firmicutes to Bacteroidetes ratios

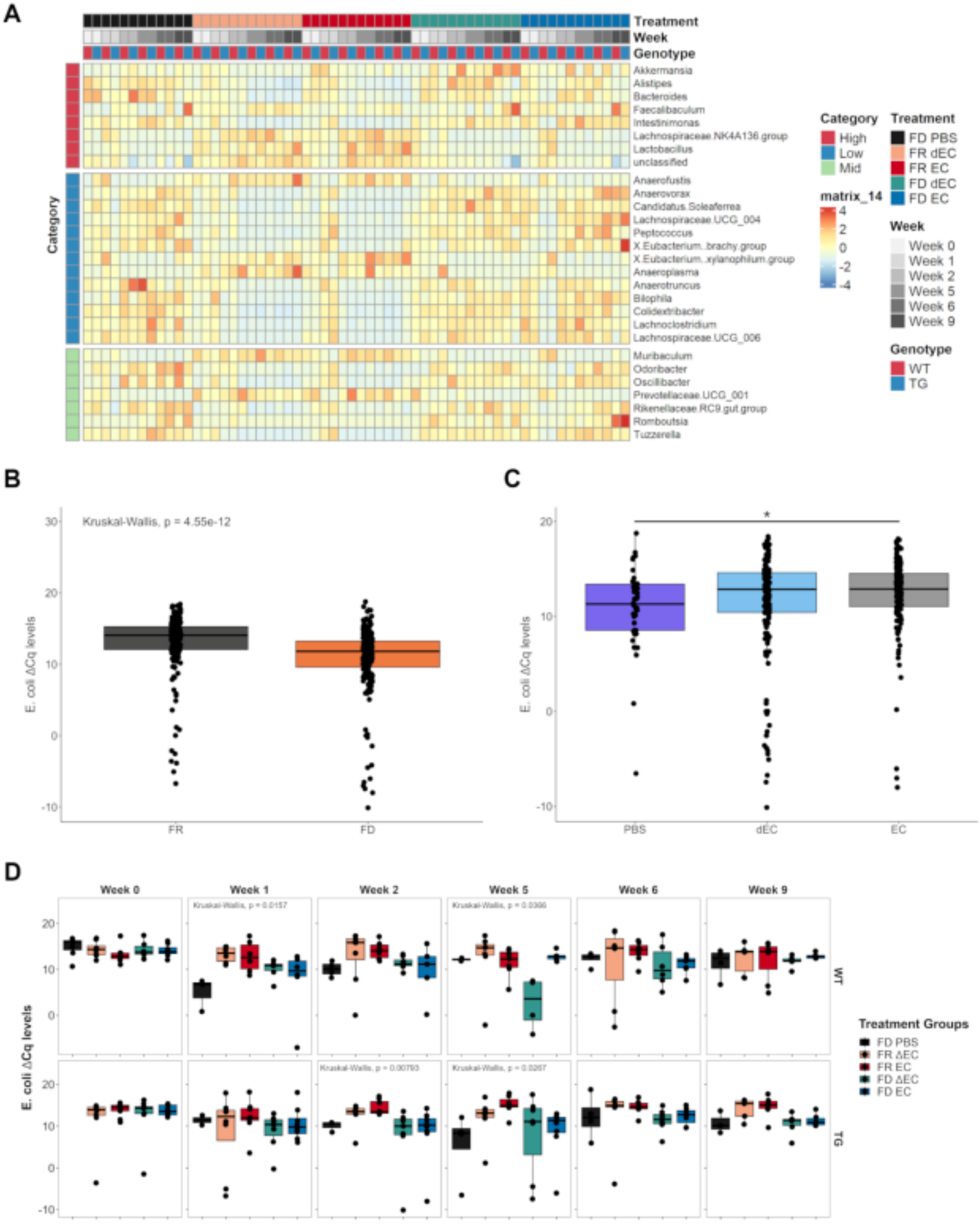

Figure S4 | Differential taxa abundance over time is diet regulated

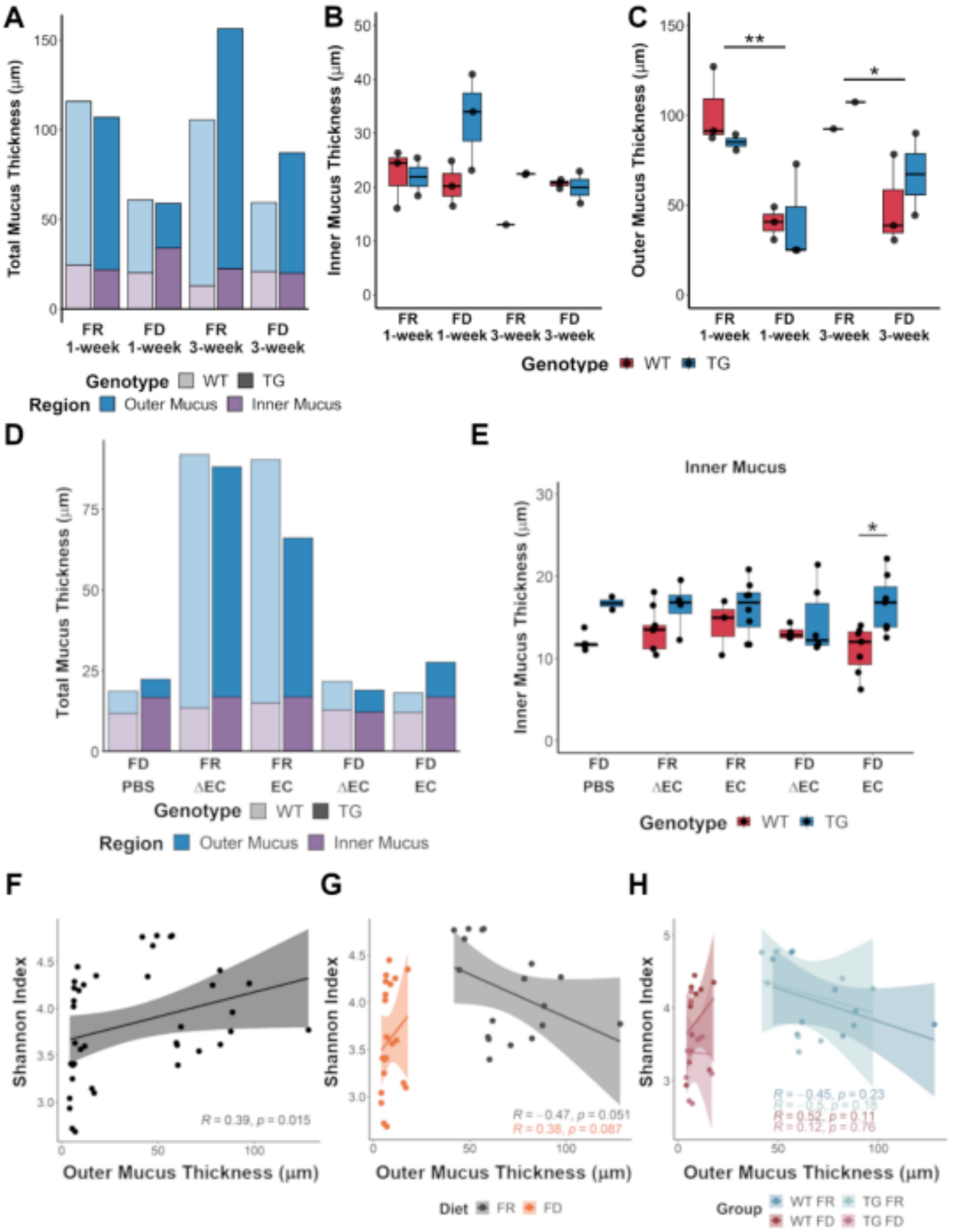

Figure S5 | Invariant inner mucus thickness and outer mucus to microbial diversity associations

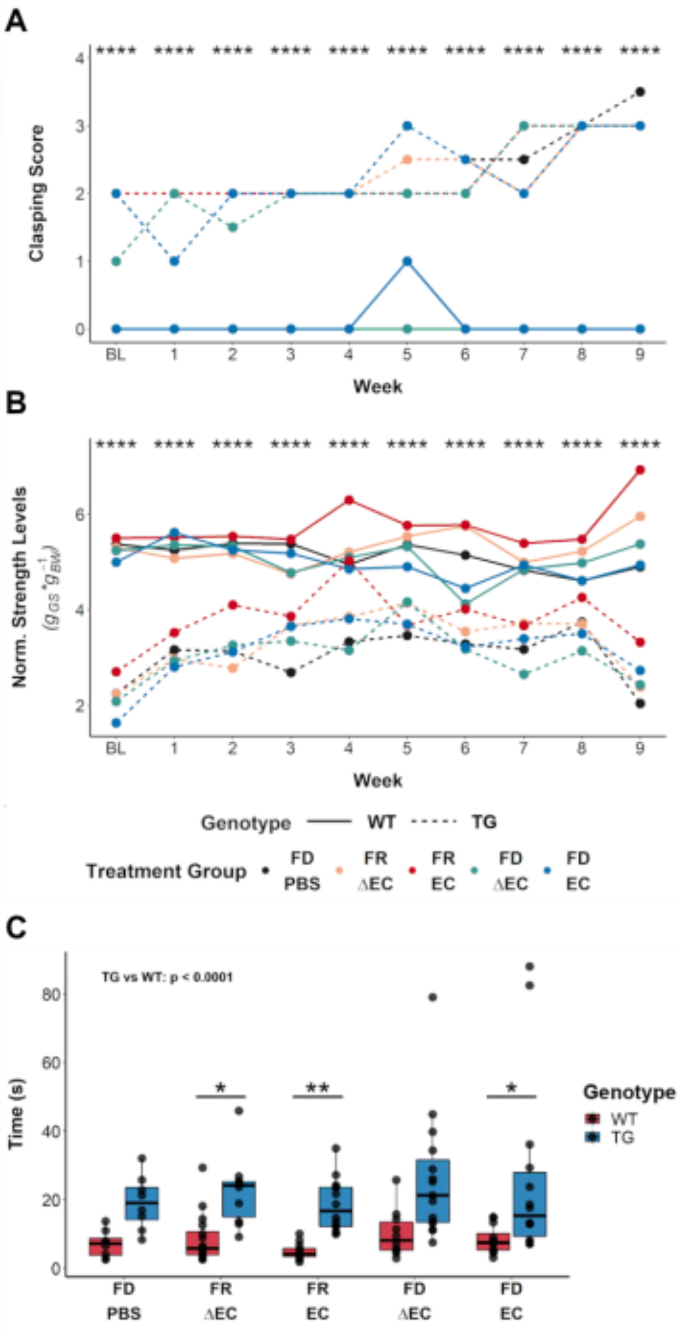

Figure S6 | Gross motor and sensorimotor functions are transgene driven

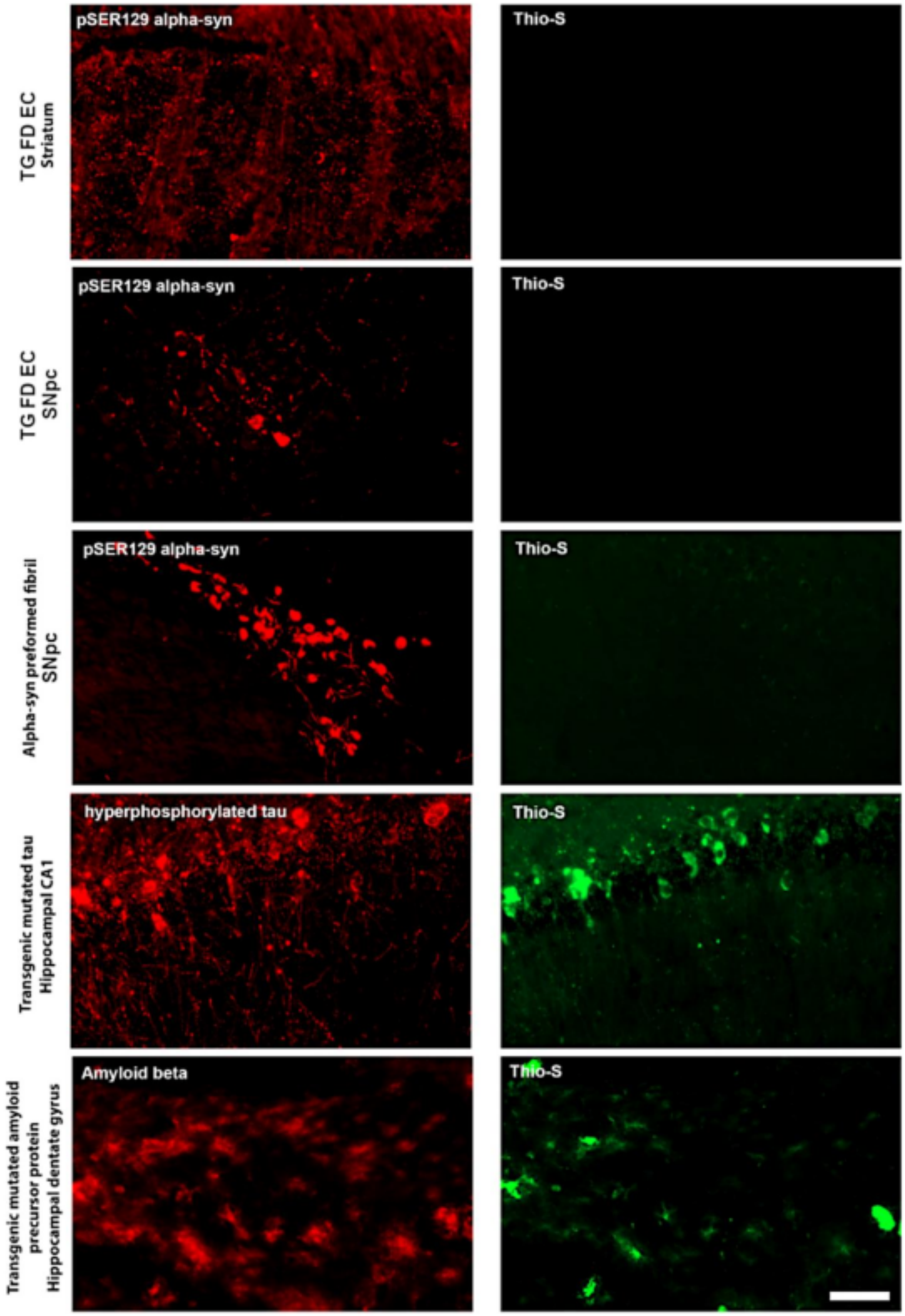

Figure S7 | Not all immunopositive pathological protein inclusions are Thio-S positive in different models of neurodegeneration.

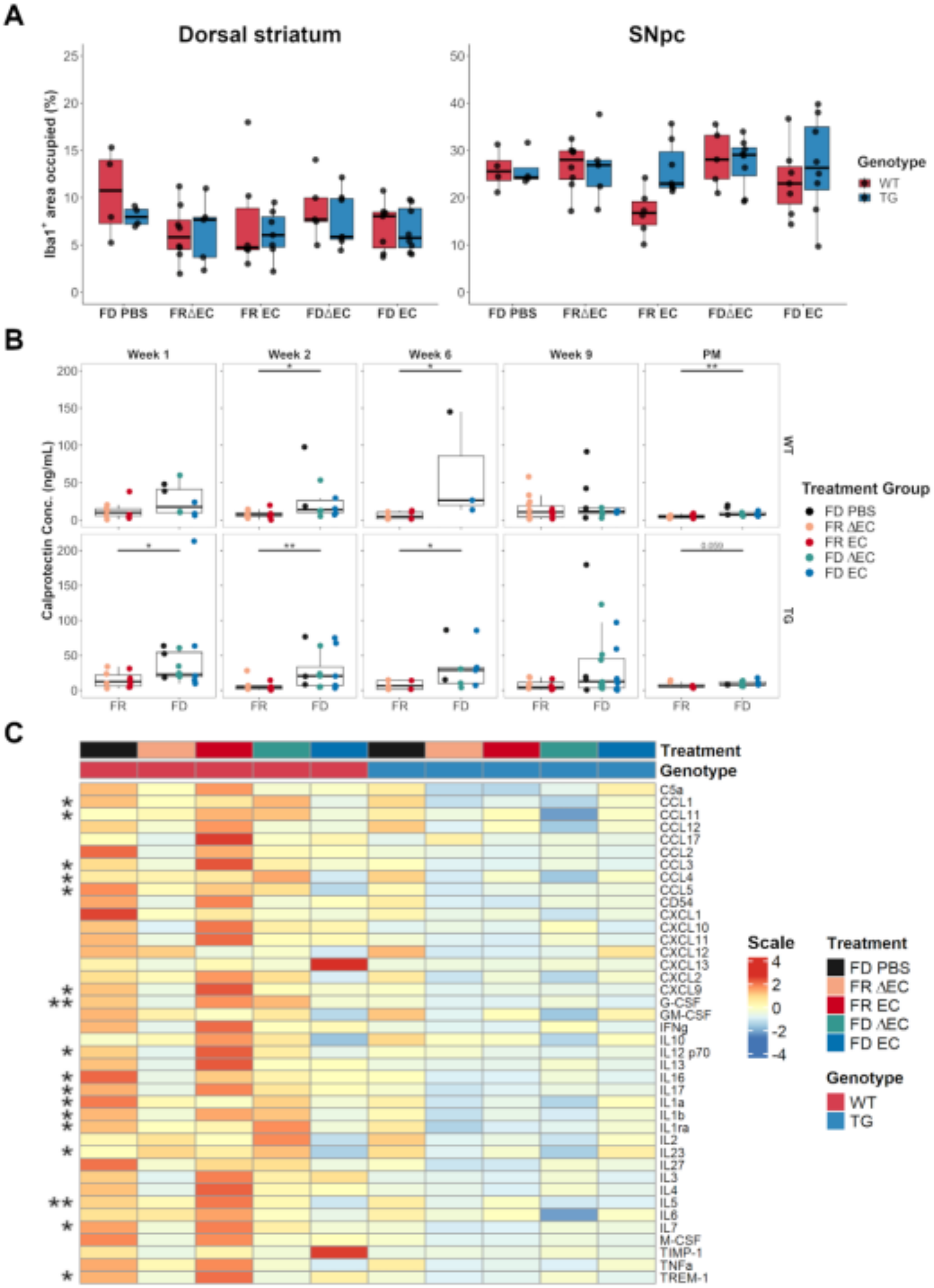

Figure S8 | Minimal or no central and peripheral inflammation in Thy1-Syn14 mice after diet and *E.coli* challenges

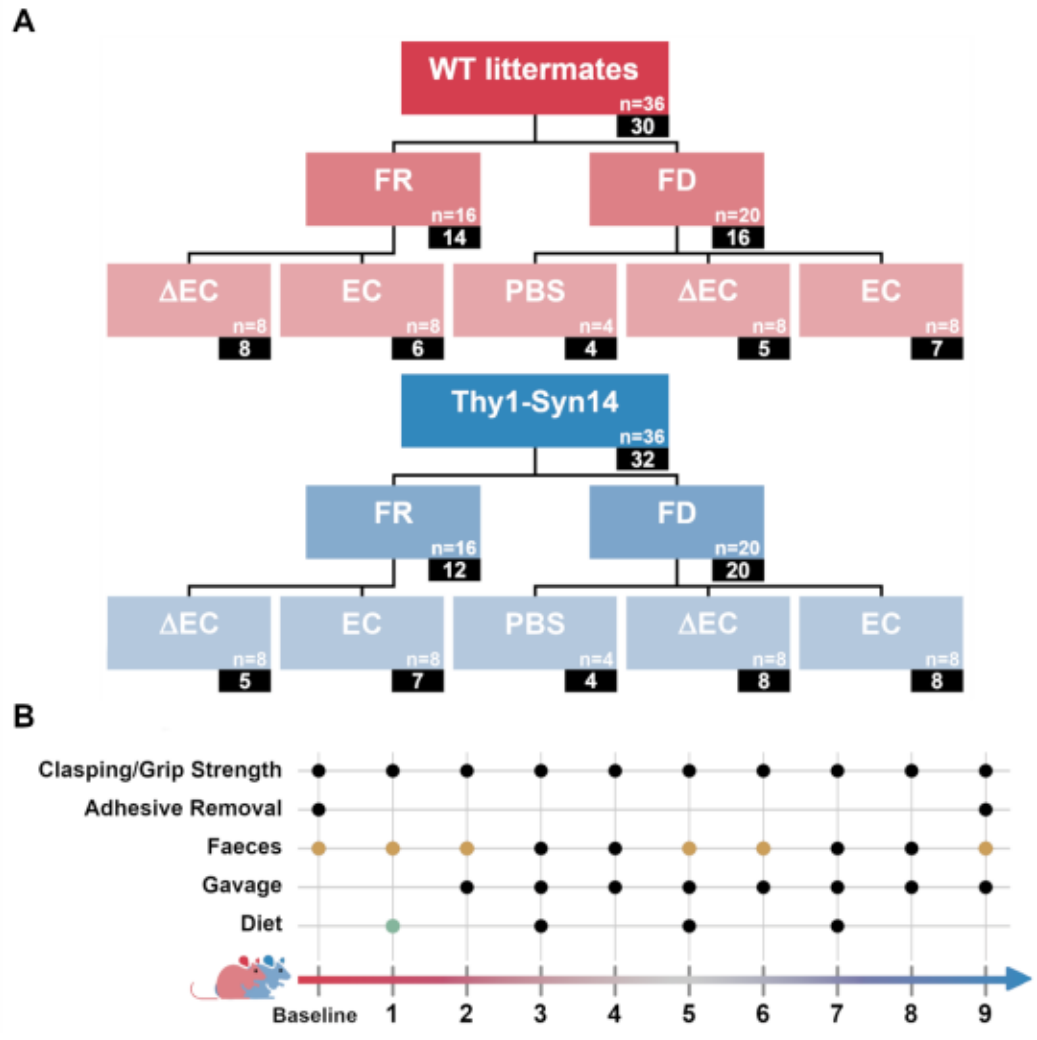

Figure S9 | Experimental design and in-life experiments

Supplementary Tables

Table S1 | Comparison of the 16S rRNA sequencing results to known taxa altered in stool samples of patients with PD (adapted from Boertien et al., 2019)

Treatment group	FR DEC						FR EC						FD DEC						FD EC					
Week	2		5		9		2		5		9		2		5		9		2		5		9	
Genotype	WT	TG	WT	TG	WT	TG	WT	TG	WT	TG	WT	TG	WT	TG	WT	TG	WT	TG	WT	TG	WT	TG	WT	TG
	↓	↓	-	↓	-	↓	-	↓	↓	↓	↓	↓	↑	↑	↑	↑	↑	↑	↓	↑	↓	↑	↓	↑
	-	↓	-	↓	-	↓	-	↓	-	↓	-	↓	↗	↑	↗	↗	↑	↑	↘	↑	↓	↑	↓	↗
	↓	↓	-	↓	-	↓	↗	↓	↓	↓	↓	↓	↗	↑	↗	↑	↑	↑	↓	↑	↓	↑	↓	↗
	↘	-	↘	↗	-	↑	↗	↑	↗	↑	↑	↑	↓	-	↓	-	↓	-	-	-	-	-	-	-
	↓	-	↘	↗	-	↑	↗	↑	↗	↑	↑	↑	↓	↘	↓	↓	↓	-	↓	-	↓	↓	↓	-
	N/A						N/A						N/A						N/A					
	N/A						N/A						N/A						N/A					
	N/A						N/A						N/A						N/A					
	N/A						N/A						N/A						N/A					
	↗	↓	↗	-	↘	-	↗	-	↗	↗	↗	↗	↘	-	↘	↗	↑	↑	-	↘	-	↘	↑	↗
	↗	↘	↗	-	↘	↘	↗	-	↗	↗	-	↗	↘	↘	↘	↘	-	↘	-	↘	↗	-	↓	-
	↗	↘	↗	↘	↗	↘	-	-	-	-	↑	↑	-	-	-	↑	↑	↑	↓	↘	↘	↘	↘	-
	-	↑	-	-	-	-	-	↗	-	-	-	↘	↓	-	↓		↓	↓	N/A					
	N/A						N/A						N/A						N/A					
	N/A						N/A						N/A						N/A					
	-	↗	-	-	-	-	↘	↗	-	-	↘	↘	-	-	-	-	↓	↓	-	↗	-	-	↓	↓
	↘	↘	↗	↗	↗	↗	↘	-	↘	-	↗	-	↘	↗	↓	↗	↑	↑	↘	↓	↘	↓	↑	↗
	↗	↘	-	↘	↘	↘	↗	↘	↘	↘	↘	-	↑	↘	↑	↘	↗	↓	-	-	↗	↗	↓	↓

Phylum	Family	Genus	FD PBS					
			2		5		9	
			WT	TG	WT	TG	WT	TG
Verrucomicrobia			↑	↑	↗	-	↑	↗
Verrucomicrobia	Akkermansiaceaea		↗	↑	↗	-	↗	↗
Verrucomicrobia	Akkermansiaceaea	Akkermansia	↑	↑	↗	-	↑	↑
Firmicutes	Lactobacillaceae		-	-	-	-	-	-
Firmicutes	Lactobacillaceae	Lactobacillus	↗	↘	↓	↓	-	-
Actinobacteria	Bifidobacteriaceae		N/A					
Actinobacteria	Bifidobacteriaceae	Bifidobacterium	N/A					
Proteobacteria	Enterobactericiaceae		N/A					
Firmicutes	Enterococcaceae		N/A					
Firmicutes			↑	-	↗	↑	↑	↑
Firmicutes	Lachnospiraceae		↗	-	-	↑	↑	-
Firmicutes	Lachnospiraceae	Roseburia	↗	-	↗	-	↗	-
Bacteriodetes	Prevotellaceae		↘	↘	↘	↘	↘	↘
Bacteriodetes	Prevotellaceae	Prevotella	N/A					
Firmicutes	Clostridiaceae	Faecalibacterium	N/A					
Bacteriodetes			↓	↘	↘	↘	↓	↓
Firmicutes	Erysipelotrichacceae		↗	↗	↘	-	↘	↑
Firmicutes	Ruminococcaceae		↗	↗	↑	↗	↗	-

Table S2 | Abbreviations for the main figures

Abbreviation	Definition
Snca	endogenous gene for aSyn
SNCA	human aSyn transgene
3M	3-month-old
6M	6-month-old
9M	9-month-old
13M	13-month-old
WT	wild-type littermates
TG	Thy1-Syn14 mice
FD	fiber-deprived
FR	fiber-rich
PBS	phosphate buffered saline solution
DEC	curli-KO E. coli
EC	wild-type curli expressing E. coli
CNS	central nervous system
ENS	enteric nervous system
SNpc	Substantia Nigra pars compacta
ZO-1	zonula occludens-1
